# Silica Nanoparticles Induced Epithelial–Mesenchymal Transition in BEAS-2B Cells via ER Stress and SIRT1/HSF1/HSPs Signaling Pathway

**DOI:** 10.3390/jox15050137

**Published:** 2025-08-23

**Authors:** Jinyan Pang, Liyan Xiao, Zhiqin Xiong, Kexin Zhang, Man Yang, Ji Wang, Yanbo Li, Yang Li

**Affiliations:** 1 School of Public Health, Capital Medical University, Beijing 100069, China; pangjinyan09@163.com (J.P.); xiaoliyan1661@163.com (L.X.); xzqdyx0718@163.com (Z.X.); kashin1029@126.com (K.Z.); many@ccmu.edu.cn (M.Y.); wangji@ccmu.edu.cn (J.W.); ybli@ccmu.edu.cn (Y.L.); 2 Beijing Key Laboratory of Environment and Aging and Laboratory for Clinical Medicine, Capital Medical University, Beijing 100069, China; 3 Tianjin Heping District Center for Disease Control and Prevention, Tianjin 300070, China

**Keywords:** amorphous silica nanoparticles, ER stress, SIRT1/HSF1/HSPs pathway, epithelial–mesenchymal transition, BEAS-2B cells

## Abstract

The extensive utilization of amorphous silica nanoparticles (SiNPs) has raised concerns regarding the potential health risks. Previous studies have indicated that SiNPs could trigger both the activation of heat shock proteins (HSPs) and epithelial–mesenchymal transition (EMT) in BEAS-2B cells; however, the underlying mechanisms require further elucidation. This study aimed to investigate how SiNPs activate the heat shock response (HSR) in BEAS-2B cells, which subsequently triggers EMT. Firstly, we observed that SiNPs were internalized by BEAS-2B cells and localized in the endoplasmic reticulum (ER), inducing ER stress. The ER stress led to the activation of SIRT1 by phosphorylation, which enhanced the nuclear transcriptional activity of HSF1 via deacetylation. HSF1 was found to upregulate the levels of HSP70 and HSP27 proteins, which further affected EMT-related genes and, ultimately, induced EMT. Additionally, 4-phenylbutyric acid (4-PBA) inhibited ER stress, which attenuated the SIRT1/HSF1 signaling pathway. The knockdown of SIRT1 and HSF1 using siRNA effectively suppressed the EMT progression. In summary, these results suggested that SiNPs activated the SIRT1/HSF1/HSPs pathway through ER stress, thereby triggering EMT in BEAS-2B cells. The present study identified a novel mechanism of SiNP-induced EMT, which has provided valuable insights for future toxicity studies and risk assessments of SiNPs.

## 1. Introduction

Amorphous silica nanoparticles (SiNPs) are widely used in various industries, including agriculture, cosmetics, food production, and biomedicine [1], owing to their specific physical and chemical properties. The mass production and use of SiNPs has significantly increased public exposure to SiNPs. In the OECD, SiNPs have been prioritized as materials requiring toxicological safety assessment. SiNPs are nanoscale particles composed of silicon dioxide (SiO_2_), with a particle size of less than 100 nm [2]. Recent studies have indicated that SiNPs could induce varying degrees of damage to the respiratory [3,4], cardiovascular [5,6], digestive [7,8], reproductive [9], and central nervous systems [10]. The primary target organ of SiNPs is the lung, and accumulating evidence indicated that SiNPs could result in the risk of developing various lung diseases [11]. SiNPs could trigger oxidative stress, ER stress, autophagy dysfunction, and inflammatory responses, leading to cytotoxic and genotoxic effects in human bronchial and alveolar epithelial cells [12,13]. Furthermore, the study has demonstrated that SiNPs could enhance the release of TGF-α, promoting the proliferation of BEAS-2B cells while inhibiting apoptosis and inducing epithelial–mesenchymal transition (EMT) [14]. Our research group has previously demonstrated that acute exposure to SiNPs induced EMT in vivo and in vitro [15]. The process of EMT is defined as the dissociation of polarized epithelial cells from their cell–cell adhesion, which is followed by the acquisition of mesenchymal characteristics, including increased cell motility and invasiveness [16]. Meanwhile, EMT is a critical process in cancer progression, including pulmonary fibrosis [17], tumorigenesis [18], and metastasis [19]. Our previous transcriptomics study has also demonstrated that SiNPs significantly upregulated non-small cell lung cancer-related signaling pathways in BEAS-2B cells and found that heat shock proteins (HSPs) played a key regulatory role in SiNP-induced cytotoxicity [20]. However, the mechanism by which HSPs trigger the process of EMT in SiNPs is currently unknown.

Under stress conditions, the endoplasmic reticulum (ER) becomes dysfunctional, causing the aggregation of unfolded or misfolded proteins and the activation of ER stress. Previous studies have indicated that SiNPs could induce ER stress, activating the unfolded protein response (UPR) through the EIF2AK3 and ATF6 pathways [21]. However, the heat shock response (HSR), which is activated by heat shock factor 1 (HSF1) in response to ER stress, is still incompletely understood. ER stress is a protective mechanism activated in response to intracellular proteostatic imbalances caused by stress. SIRT1, a deacetylase that is dependent on NAD+, has been shown to regulate multiple signaling pathways by modulating the acetylation status of target proteins. Studies have demonstrated that SIRT1 could be activated during ER stress, thereby mitigating cellular damage caused by excessive stress through the regulation of HSR and UPR [22,23]. Additionally, knocking down the SIRT1 gene or adding a SIRT1 inhibitor (EX527) significantly reduced the tolerance of cells to ER stress. Conversely, pretreatment with the SIRT1 activator STAC-3 effectively alleviated ER stress-induced cell death [23]. In response to ER stress, SIRT1 could inhibit the eEF2K/eEF2 pathway through deacetylation of eIF2α, preventing apoptosis induced by prolonged UPR activation [22]. On the other hand, SIRT1 could activate HSF1, which upregulated the expression of HSPs to enhance the cellular anti-stress and anti-apoptosis abilities [24].

Recent studies have demonstrated that the nuclear transcriptional activity of HSF1 was predominantly stimulated by modifications such as phosphorylation and deacetylation [25]. Deacetylation of lysine at the K80 site of the HSF1 DNA-binding domain by SIRT1 has been shown to enhance the binding of HSF1 to DNA, as well as the protein stability of HSF1 [26]. Conversely, transfection with SIRT1 small interfering RNA (siRNA) has been observed to induce excessive acetylation of the HSF1 protein within the cell [27]. The nuclear transcriptional activity of HSF1 is activated under stress, which can rapidly enter the nucleus and transiently synthesize a large number of HSPs to repair or degrade abnormal proteins and protect cells from damage. In addition, HSF1 promoted cell migration and EMT, which are critical steps in tumor cell invasion and metastasis [28]. Several studies have demonstrated that HSF1 facilitated metastatic progression in various tumor models, such as lung cancer [29], breast cancer [30], HCC [31], and melanoma [32]. In Hsf1+/+ mice expressing the HER2/NEU oncogene, the process of transforming growth factor-β (TGF-β)-stimulated EMT was observed to increase the expression of EMT-related markers, including Slug and vimentin [30]. In contrast, the induction of EMT by TGF-β was found to be compromised in Hsf1-/- cells. Furthermore, the knockdown of HSF1 suppressed the transcription of key EMT-inducing genes, including Slug, Snail, Twist1, and Zeb1, which have been identified as crucial factors in the suppression of TGF-β-induced EMT and migration in ovarian cancer cells [33].

Therefore, we hypothesized that SiNPs might induce ER stress and further activate SIRT1, which modulated the HSF1/HSPs pathway to promote EMT. In this study, we investigated the effects of SiNPs on the ER structure and stress responses, assessed the role of SIRT1 in ER stress activation, and used the ER stress inhibitor 4-PBA for reverse validation. The downstream HSF1/HSPs signaling pathway was then examined for its effect on SiNP-induced EMT. SIRT1 siRNA and HSF1 siRNA were used to confirm the relationship between the SIRT1/HSF1/HSPs signaling pathway and EMT induced by SiNPs.

## 2. Materials and Methods

### 2.1. Chemicals and Antibodies

Mouse anti-CHOP (#2895, Beverly, MA, USA), rabbit anti-BIP (#3177, Beverly, MA, USA), rabbit anti-SIRT1 (#9475, Beverly, MA, USA), rabbit anti-phospho-SIRT1 (ser47) (#2314, Beverly, MA, USA), rabbit anti-HSF1 (#12972, Beverly, MA, USA), rabbit anti-Acetylated-Lysine (#9441, Beverly, MA, USA), rabbit anti-HSP90 (#4874, Beverly, MA, USA), rabbit anti-HSP70 (#4872, Beverly, MA, USA), and rabbit anti-N-cadherin (#13116, Beverly, MA, USA) antibodies were purchased from Cell Signaling Technology. Mouse anti-E-cadherin (#sc-8426, Dallas, TX, USA) antibody was purchased from Santa Cruz Biotechnology. Rabbit anti-phospho-SIRT1 (ser27) (#YP1495, Immunoway, TX, USA) antibody was purchased from Immunoway. Mouse anti-GAPDH (#60004-1-Ig, Wuhan, China) antibody was purchased from Proteintech. Rabbit anti-Hsp27 (#ab5579, Cambridge, UK) antibody was obtained from Abcam. Goat anti-mouse IgG IRDye 680RD (#926-68070, Lincoln, NE, USA) and goat anti-rabbit IgG IRDye 800CW (#926-32211, Lincoln, NE, USA) antibodies were obtained from Li-Cor Biosciences. 4-phe-nylbutyric acid (4-PBA, #P21005, St. Louis, MO, USA) was purchased from Sigma Aldrich.

### 2.2. Preparation and Characterization of SiNPs

The experimental synthesis of SiNPs was performed using the Stöber method, as previously demonstrated in our study [34]. The mass concentration of SiNPs was 11 mg/mL, and the stock solution was stored at 4 °C. The stock solution was subsequently diluted to the requisite concentrations when BEAS-2B cells were exposed to SiNPs. The morphology of SiNPs was observed by transmission electron microscopy (TEM, JM-2100, Tokyo, Japan) following the drying of SiNPs dripped onto the copper mesh. The average particle size of SiNPs was then calculated using ImageJ 2.14.0/1.54f software. The purity of SiNPs and the content of impurity elements were analyzed by inductively coupled plasma-atomic emission spectrometry (ICP-AES, Thermo Fisher Scientific ARL 3520, Neuchâtel, Switzerland) to confirm the purity of the synthesized SiNPs.

### 2.3. Cell Culture, Treatment, and Transfection

The human bronchial epithelial cell line, BEAS-2B cells, was purchased from the Cell Resource Center of the Shanghai Institutes for Biological Sciences (SIBS, Shanghai, China). The BEAS-2B cells were cultured in Dulbecco’s Modified Eagle Medium (DMEM, Corning, NY, USA) supplemented with 10% fetal bovine serum (PAN, Aidenbach, Bavaria, Germany), 100 U/mL penicillin, and 100 µg/mL streptomycin. The cells were maintained in a humidified incubator at 37 °C, with the addition of 5% CO_2_. For experiments, the cells were plated in Corning cell culture dishes (100 mm × 20 mm) at a density of approximately 1 × 10^5^ cells/mL and incubated for 24 h before treatment with SiNPs. SiNPs were sonicated (160 W, 20 kHz, 5 min) by a sonicator (Bioruptor UDC-200, Liège, Belgium) to make the particles evenly dispersed and diluted in DMEM to different concentrations (5, 10, and 20 μg/mL) and then immediately added to BEAS-2B cells. BEAS-2B cells free of serum and SiNPs were used as the control group.

For SIRT1 and HSF1 knockdown, SIRT1 and HSF1 siRNAs were designed and synthesized by Oligobio (Beijing, China). BEAS-2B cells were transfected according to the manufacturer’s protocol of the Lipofectamine 3000 reagent (Invitrogen, #L3000015, Carlsbad, CA, USA). BEAS-2B cells were seeded into 6-well plates at a density of 2 × 10^5^ cells per well. After 48 h of transfection, the cells were washed with phosphate-buffered saline (PBS) three times, and trypsin digestion was performed to harvest the cells. The transfection efficiency of the siRNAs was then detected by Western blot. Non-targeting siRNA (sequence: TTCTCCGAACGTGTCACGT) was used as a negative control and incorporated into all siRNA experiments, with treatment conditions identical to those of the targeted siRNA group. Each group had at least three replicate wells.

### 2.4. Observation of Endoplasmic Reticulum by TEM

In this study, the internalization of SiNPs into cells and the effects on the ultrastructure of the ER were observed by TEM. BEAS-2B cells were treated with 10 µg/mL SiNPs for 24 h, followed by three washes with PBS to eliminate residual SiNPs. The cells were subjected to a centrifugation step at 1200 rpm for 10 min after digesting the cells with trypsin. Subsequently, the cells were fixed in 2.5% glutaraldehyde at 4 °C for 2 h and washed thrice with 0.1 M PB buffer. The cells were post-fixed in 1% osmium tetroxide for 1 h and rinsed with PB buffer. Additionally, the cells were subjected to a series of graded dehydration solutions, including 50%, 70%, 80%, 90%, and 100% ethanol, followed by two washes with 100% acetone, and then embedded in epoxy resin. Ultrathin sections (50–70 nm) were cut using an ultramicrotome (Ultracut UCT, Leica, Wetzlar, Germany). They were stained with uranyl acetate and lead citrate solutions. The samples were subsequently dried and imaged using TEM (JM-1400, Tokyo, Japan).

### 2.5. BEAS-2B Cells Viability Analysis

The viability of BEAS-2B cells exposed to SiNPs with different concentrations and exposure times was determined by a Cell Counting Kit-8 (CCK-8, Dojindo, Kumamoto, Japan) assay, according to the manufacturer’s instructions. BEAS-2B cells were seeded in 96-well plates at a density of 1 × 10^4^ cells per well and cultured for 24 h. Following sonication, SiNPs were first diluted from a concentration of 11 mg/mL to 80 μg/mL using DEME, followed by further dilution to different concentrations (2.5, 5, 10, 20, and 40 μg/mL) using a gradient dilution method. The solutions were then added to a 96-well plate and incubated for 12 and 24 h, respectively. Additionally, 10 μL of CCK-8 solution was added to each well, and the cells were incubated at 37 °C for another 1 h. The optical density at 450 nm was detected using a microplate reader (Thermo, Waltham, MA, USA).

### 2.6. Immunofluorescence Analysis

BEAS-2B cells were cultured in confocal dishes at a density of 1 × 10^5^ cells per dish and then exposed to various concentrations of SiNPs for 24 h. Following this, the cells were fixed using 4% paraformaldehyde (Solarbio, #P1110, Beijing, China) for 10 min, permeabilized with 0.1% Triton X-100 in PBS for 10 min, and blocked with 10% fetal bovine serum for 1 h in order to eliminate nonspecific binding sites. The cells were then incubated with the primary antibody HSF1 (1/200 dilution) at 4 °C overnight, followed by incubation with the donkey anti-rabbit fluorescent secondary antibody (Alexa Fluor 647) (1/400 dilution) for another 1 h away from light. Confocal dishes were washed three times with 0.01 M PBS for 5–10 min on a shaking platform each time when changing different reagents for the experiment. Subsequently, the cells were incubated with DAPI for 15 min at room temperature. The morphological examination was randomly conducted using a laser scanning confocal microscope (LSCM, Leica TCS SP8, Wetzlar, Germany). The nuclear-to-cytoplasmic fluorescence intensity ratio was then quantitatively analyzed on the acquired images using ImageJ software.

### 2.7. Western Blot Analysis

BEAS-2B cells were lysed using the radioimmunoprecipitation assay (RIPA, Solarbio, #BC3710, Beijing, China) lysis buffer to obtain the total cell proteins. The protein concentration was determined using a BCA assay kit (Ding Guo BioTECH, Beijing, China). Equivalent amounts of lysate proteins were subjected to separation by 10% sodium dodecyl sulfate-polyacrylamide gel electrophoresis (SDS-PAGE, New Saimei, Suzhou, China) and subsequently transferred onto nitrocellulose (NC) membranes. The membranes were then blocked with Rapid Blocking Solution (New Saimei, Suzhou, China) for 10 min at room temperature, following the manufacturer’s protocol. Following blocking, the membrane was incubated overnight at 4 °C with various primary antibodies, which had been diluted according to the manufacturer’s instructions. The next step was an incubation at room temperature with 0.1 μg/mL of IRDye-labeled secondary antibody for 1 h. NC membranes were washed three times with 0.01M PBS for 5–10 min each time on a shaking platform when changing different reagents for the experiment. The protein bands were subjected to visualization using the Li-COR Odyssey imaging system (LI-COR Biosciences, Lincoln, NE, USA) and quantified by densitometry analysis using ImageJ software. At least three independent experiments were conducted to ensure reproducibility and obtain representative results.

### 2.8. Immunoprecipitation Analysis

Total cell protein lysates were prepared by lysing cells in immunoprecipitation lysis buffer (Beyotime, #P0013, Shanghai, China), which was complemented with 1 mM PMSF (Beyotime, #ST507, Shanghai, China). The immunoprecipitation of the HSF1 protein was conducted through an incubation process with an anti-HSF1 antibody and µMACS Protein G MicroBeads (#130-071-101, Miltenyi Biotec, Bergisch Gladbach, Germany). The magnetic bead–antibody–HSF1 complexes were subsequently captured using a µ-column (#130-042-701, Miltenyi Biotec, Bergisch Gladbach, Germany)and a µMACS Separation Unit (#130-042-602, Miltenyi Biotec, Bergisch Gladbach, Germany). Subsequent to collection, the column was washed with lysis buffer, followed by a low-salt wash buffer, in order to remove non-specific binding. In the subsequent stage of the procedure, the 1× SDS gel loading buffer was preheated to 95 °C and then applied to the column matrix in order to elute the immunoprecipitates. The eluted samples were then subjected to analysis by Western blot using anti-HSF1 and anti-acetylation antibodies. The relative abundance of acetylated HSF1 was then quantified by calculating the ratio of acetylated HSF1 to total HSF1, thereby reflecting the changes in HSF1 acetylation levels.

### 2.9. RNA Extraction and Real-Time PCR

Total RNA samples were extracted using the RNAeasy^TM^ Animal RNA Extraction Kit (Biyuntian, Shanghai, China) according to the method provided by the manufacturer. Reverse transcription was performed in 10 μL with 500 μg of total RNA according to the kit (Prime Script TMRT reagent Kit, TaKaRa, Kyoto, Japan). All reverse-transcribed cDNA samples were used immediately or stored at −80 °C until the next use. Quantitative real-time PCR was performed to detect mRNA expression of EMT-associated proteins, including E-cadherin, Twist, MMP-9, and MMP-3. NovoStart SYBR qPCR SuperMix Plus (Novoprotein, Suzhou, China) was used in the real-time PCR system for fluorescence quantitative PCR. The system was added with 2 μL of cDNA stock solution to 20 μL of the reaction system, and the PCR reaction was performed on a 7900HT Fast RealTime PCR instrument (Life Technologies, Grand Island, NY, USA). Fold change of mRNA expression was analyzed using the 2 (−ΔΔCt) method, with GAPDH as an internal control. Each experiment was performed in triplicate. The primer sequences for the qPCR reaction are presented in Table 1.

### 2.10. Data Analysis

All data are expressed as mean ± S.D. All experiments were repeated at least 3 times. One-way analysis of variance (ANOVA), Dunnett, and Tukey’s post hoc tests were used to analyze the data from the respective experiments using GraphPad Prism 9.0.0. Statistical significance was defined as *p* < 0.05.

## 3. Results

### 3.1. Characterization and Cytotoxicity of SiNPs

**Figure 1:**
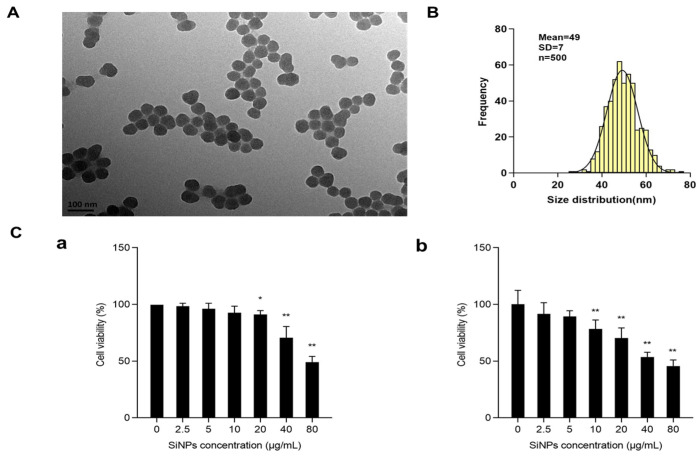
Characterization and cytotoxicity of SiNPs. (**A**) Representative TEM image of SiNPs. SiNPs showed a spherical shape and good monodispersity in distilled water. (**B**) Average size and size distribution of SiNPs. The average size of SiNPs was 49 ± 7 nm, as measured by ImageJ. (**C**) The viability of BEAS-2B cells after exposure to SiNPs with different concentrations (2.5, 5, 10, 20, 40, and 80 µg/mL) for 12 h and 24 h by the CCK-8 kit. (**a**) The cell viability of BEAS-2B cells after exposure to SiNPs for 12 h. (**b**) The cell viability of BEAS-2B cells after exposure to SiNPs for 24 h. *n* = 6, data are expressed as mean ± S.D. * *p* < 0.05 and ** *p* < 0.01, SiNP-treated group compared to the control group.

The morphology of SiNPs was characterized by TEM, and the results showed that SiNPs were nearly spherical and well dispersed (Figure 1A; File S1), with a calculated average particle size of about 49 ± 7 nm (Figure 1B). The purity of SiNPs detected by ICP-AES was higher than 99.9%. The above results indicated that the SiNPs possessed a uniform shape and fairly good monodispersity. The cytotoxicity of SiNPs was measured using a CCK-8 kit in BEAS-2B cells treated with different concentrations (2.5, 5, 10, 20, 40, and 80 μg/mL) of SiNPs. The cell viability decreased with the increasing concentrations of SiNPs in a dose-dependent manner (Figure 1C). The results in Figure 1C(a) showed that, after 12 h of SiNP exposure, the viability of BEAS-2B cells decreased in a dose-dependent manner. In the 20, 40, and 80 µg/mL SiNP-treated groups, the cell viability was significantly reduced (*p* < 0.05) compared to the control group. The results in Figure 1C(b) showed that, after BEAS-2B cells were exposed to SiNPs for 24 h, the cell viability decreased in a dose-dependent manner, and statistical differences were observed starting from the 10 μg/mL-treated group (*p* < 0.05) compared to the control group. Therefore, the above results indicated that an increased concentration of SiNPs led to a decline in cell survival proportion at 12 h and 24 h of exposure. Additionally, the concentrations of 5, 10, and 20 µg/mL of SiNPs were selected based on the 24-h cytotoxicity results, which indicated short-term cellular effects in BEAS-2B cells.

### 3.2. SiNPs Induced ER Stress and Activated the SIRT1/HSF1 Signaling Pathway

**Figure 2:**
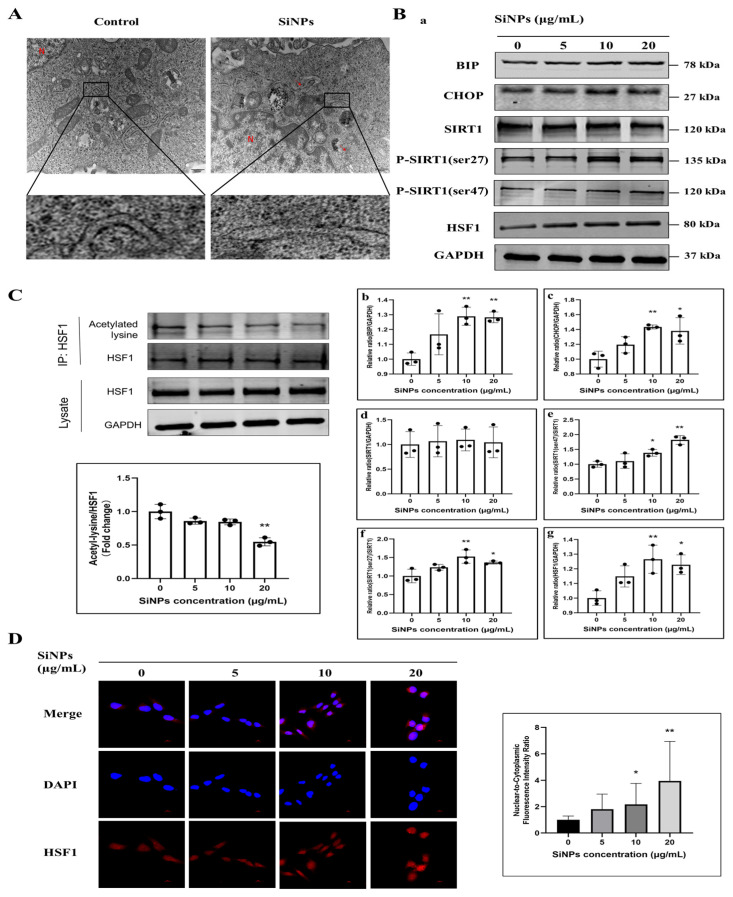
SiNPs caused ER damage, induced ER stress, and further activated the SIRT1/HSF1 signaling pathway. (**A**) The ER structure was observed by TEM after SiNPs treatment in BEAS-2B cells. Scale bar: 500 nm. Red arrow, ER. N, nucleus. (**B**) Measurement of ER stress and SIRT1/HSF1 signaling pathway-related proteins after 24 h of SiNPs treatment in BEAS-2B cells by Western blot. The GAPDH was used as an internal control to assess the equal loading of the samples. (**a**) Representative protein bands of BIP, CHOP, SIRT1, SIRT1 (ser47), SIRT1 (ser27), and HSF1 were detected by Western blot. (**b**–**g**) Grayscale values of the expression of ER stress and SIRT1/HSF1 signaling pathway-related proteins affected by SiNPs, as analyzed by ImageJ. *n* = 3. Data were expressed as mean ± S.D., * *p* < 0.05 and ** *p* < 0.01, SiNP-treated group compared with the control group. (**C**) The acetylated HSF1 was detected by immunoprecipitation after SiNPs treatment for 24 h. (**D**) The localization and quantitative analysis of HSF1 in the nucleus of BEAS-2B cells after SiNPs treatment, which was detected by IF. Blue: DAPI; red: HSF1, scale bar: 10 μm.

In order to explore whether SiNPs could induce ER damage and ER stress, the ultrastructure of the ER was observed by TEM, and the changes in ER stress protein markers were detected in BEAS-2B cells after SiNP exposure. As shown in Figure 2A and File S1, the TEM results showed that a large number of ribosomes were observed on the surface of the rough ER in the control group, but the ribosomes were significantly reduced and segregated in the ER of the SiNP-treated group. Meanwhile, the expression of ER stress markers, BIP and CHOP, was detected by Western blot. As shown in Figure 2B and File S1, the results demonstrated that the expression of BIP and CHOP proteins remarkably increased, and the differences were statistically significant in the 10 and 20 μg/mL SiNP-treated groups (*p* < 0.05). These findings indicated that exposure to SiNPs might induce ER stress in BEAS-2B cells.

Additionally, the protein expression levels of the phosphorylation of SIRT1, containing the ser27 and ser47 phosphorylation sites, were detected by Western blot to reflect SIRT1 activity. As shown in Figure 2B and File S1, it was observed that the phosphorylated SIRT1 protein expression tended to increase in a dose-dependent manner, which indicated that the deacetylase SIRT1 might be activated through phosphorylation. Similarly, the level of HSF1 protein exhibited an upward trend following SiNPs treatment and apparently increased the protein level of HSF1 at 10 and 20 µg/mL (*p* < 0.05). As SIRT1 acts as a protein deacetylase, the acetylation status of HSF1 was determined using immunoprecipitation. The acetylation state of HSF1 was gradually downregulated with the increase in the doses of SiNPs (Figure 2C; File S1). Meanwhile, the localization of HSF1 in the cytoplasm and nucleus was observed by immunofluorescence staining, and it was found that HSF1 gradually transferred into the nucleus with the increasing doses of SiNPs (Figure 2D; File S1), which indicated that SiNPs might promote the nuclear transcriptional activity of HSF1. Therefore, the above results suggested that exposure to SiNPs induced ER stress in BEAS-2B cells and then activated SIRT1 by phosphorylation, which further regulated the protein expression and nuclear transcriptional activity of HSF1.

### 3.3. 4-PBA Inhibitied the ER Stress and Further SIRT1/HSF1 Signaling Pathway Activation of BEAS-2B Cells Induced by SiNPs

**Figure 3:**
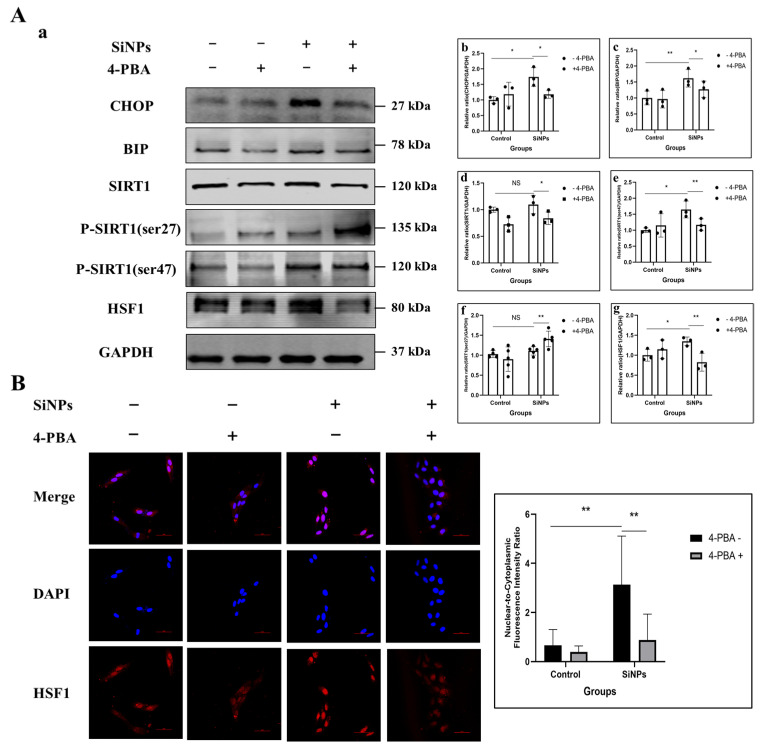
Effect of 4-PBA pretreatment on the SIRT1/HSF1 signaling pathway and HSF1 localization in the nucleus after SiNPs exposure. (**A**) 4-PBA pretreatment (5 mM) inhibited ER stress in BEAS-2B cells after SiNPs (10 μg/mL) exposure. (**a**) Representative protein bands of CHOP, BIP, SIRT1, SIRT1 (ser47), SIRT1 (ser27), and HSF1 were detected by Western blot. (**b**–**g**) Grayscale values of the SIRT1/HSF1 protein expression levels were analyzed by ImageJ. *n* = 3–5. Data were expressed as mean ± S.D., * *p* < 0.05 and ** *p* < 0.01, SiNP-treated group compared with the corresponding SiNPs plus 4-PBA group or SiNP-treated group without 4-PBA compared with the control group without 4-PBA. NS = not significant. (**B**) The localization and quantitative analysis of HSF1 in BEAS-2B cells pretreated with or without 4-PBA after SiNPs treatment. Blue: DAPI; red: HSF1, scale bar: 50 μm.

It has been demonstrated that 4-PBA has the potential to inhibit ER stress in BEAS-2B cells, as evidenced by previous studies [35,36]. To further identify whether SiNPs regulated the downstream SIRT1/HSF1 signaling pathway through the activation of ER stress, BEAS-2B cells were pretreated with 4-PBA (5 mM) for 2 h to inhibit ER stress before exposure to SiNPs. Subsequently, ER stress-related proteins were detected, as well as SIRT1/HSF1 expression levels and the nuclear transcriptional activity of HSF1. The experimental groups were set as the control group, 5 mM 4-PBA pretreated group, 10 µg/mL SiNPs treated group, and 10 µg/mL SiNPs plus 5 mM 4-PBA pretreated group. As shown in Figure 3A and File S1, the level of the deacetylase SIRT1 protein was found to be significantly reduced (*p* < 0.05) in the SiNPs plus 4-PBA pretreatment group. Additionally, there was a decrease in the phosphorylated ser47 site of SIRT1, while the phosphorylated ser27 site of SIRT1 exhibited a trend of upregulation. The above results indicated that 4-PBA could effectively decrease the protein expression of SIRT1 and its activity by inhibiting ER stress. However, the 4-PBA inhibitor upregulated the ser27 site of the SIRT1 protein, and the relationship between ER stress and SIRT1-related active sites needed to be further explored. In addition, the immunofluorescence analysis of the HSF1 protein in BEAS-2B cells showed that, in the SiNP-treated group, the HSF1 protein was colocalized extensively with the nucleus, but this phenomenon was mitigated in the SiNPs plus 4-PBA pretreated group (Figure 3B; File S1), suggesting that 4-PBA reduced the localization of HSF1 in the nucleus by inhibiting ER stress. The above results suggested that 4-PBA effectively suppressed ER stress and attenuated both the phosphorylation-mediated activation of SIRT1 and the expression and nuclear transcriptional activity of HSF1. These findings further elucidated that SiNPs activated the SIRT1/HSF1 signaling pathway through ER stress.

### 3.4. SiNPs Activated the HSF1/HSPs Signaling Pathway and Induced EMT in BEAS-2B Cells

**Figure 4:**
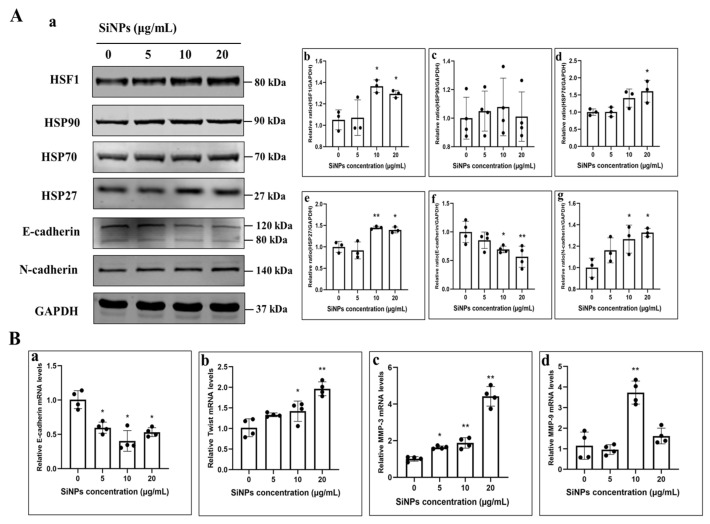
SiNPs induced EMT in BEAS-2B cells through the HSF1/HSPs signaling pathway. (**A**) Western blot results of EMT and HSF1/HSPs pathway-related proteins in BEAS-2B cells after 24 h of SiNPs exposure. (**a**) Representative images of Western blot. (**b**–**g**) Grayscale analysis of EMT-related proteins and the HSF1/HSPs signaling pathway affected by SiNPs using ImageJ, including HSF1, HSP90, HSP70, HSP27, E-cadherin, and N-cadherin, *n* = 3–4, and the data were expressed as mean ± S.D. (**B**) PCR results of EMT-related genes in BEAS-2B cells after 24 h of SiNPs exposure. (**a**–**d**) E-cadherin, Twist, MMP-3, and MMP-9. * *p* < 0.05 and ** *p* < 0.01, SiNP-treated group compared with the control group.

To further explore the mechanism of EMT in BEAS-2B cells induced by SiNPs, the expression levels of the HSF1/HSPs signaling pathway and EMT-related proteins were determined by Western blot in BEAS-2B cells treated with different concentrations of SiNPs for 24 h. As shown in Figure 4A and File S1, the protein expression of HSF1 was significantly upregulated at SiNPs concentrations of 10 μg/mL and 20 μg/mL (*p* < 0.05). HSF1, as a major regulator of the HSR, mediates the expression of stress proteins when cells are stimulated to protect themselves and survive the acute stress. In the study, the changes in HSP90, HSP70, and HSP27 protein levels were also detected in BEAS-2B cells. The results found that HSP27 considerably increased, with statistically significant differences in the 10 and 20 μg/mL SiNP-treated groups (*p* < 0.05). HSP70 protein was only statistically different in the 20 μg/mL SiNP-treated group (*p* < 0.05), but HSP90 seemed not to be influenced by SiNPs (Figure 4A; File S1). In addition, the cellular epidermal junction marker E-cadherin and the mesenchymal cell markers, N-cadherin proteins, were detected by Western blot. The results indicated a gradual decrease in E-cadherin protein levels, while the N-cadherin levels increased with increasing doses of SiNPs (Figure 4A; File S1). Subsequently, the changes in EMT-related genes were determined by PCR. The results found that the E-cadherin gene showed a decreasing trend with the increasing doses of SiNPs, while MMP-3 and Twist showed an increasing trend with the increasing doses (Figure 4B), suggesting that SiNPs might induce a decrease in intercellular adhesion and the subsequent acquisition of mesenchymal cell markers, leading to the development of EMT. The above experimental results suggested that SiNPs possibly induced the HSR by activating HSF1, further leading to the development of EMT in BEAS-2B cells.

### 3.5. SIRT1 siRNA Inhibited SIRT1 Phosphorylation and Further Suppressed HSF1 Protein Activity Induced by SiNPs

**Figure 5:**
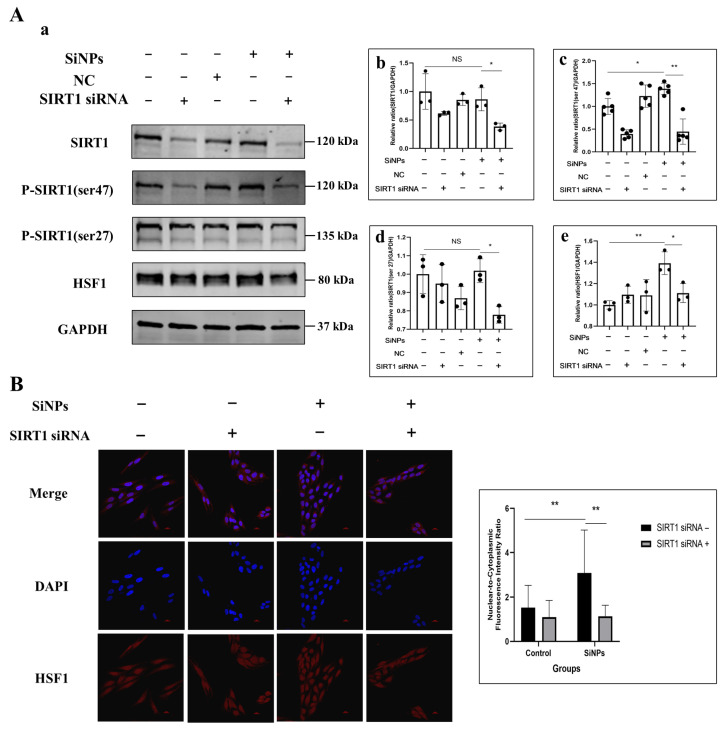
Effect of SiNPs on the SIRT1/HSF1 signaling pathway in BEAS-2B cells with SIRT1 knockdown through SIRT1 siRNA. (**A**) The BEAS-2B cells pretreated with or without SIRT1 siRNA were exposed to 10 µg/mL SiNPs. Non-targeting siRNA was used as a negative control (NC). (**a**) Representative protein bands of SIRT1, SIRT1 (ser47), SITR1 (ser27) and HSF1 were detected by Western blot. (**b**–**e**) Western blot analysis of SIRT1, SIRT1 (ser27), SIRT1 (ser47), and HSF1 protein expression levels and quantitative analysis. Data are expressed as mean ± S.D., *n* = 3–5, * *p* < 0.05 and ** *p* < 0.01. SiNPs treated group compared with corresponding SiNPs plus SIRT1 siRNA group or SiNP-treated group without SIRT1 compared with the control group without SIRT1 siRNA. (**B**) Effect of SIRT1 knockdown on the intracellular localization and quantitative analysis of HSF1 after SiNPs exposure. Blue: DAPI; red: HSF1. Scale bar: 20 μm.

To investigate the relationship between SIRT1/HSF1 signaling pathway activation and SiNP-induced EMT, SIRT1 was knocked down in BEAS-2B cells using SIRT1 siRNA transfection. BEAS-2B cells were pretreated with 50 µM SIRT1 siRNA to inhibit the activity of SIRT1 protein. Following this, changes in the level of SIRT1 and HSF1 proteins were determined by Western blot. The experimental groups were set as control group, 50 μM SIRT1 siRNA pretreated group, NC group, 10 μg/mL SiNPs treated group, and 10 μg/mL SiNPs plus 50 μM SIRT1 siRNA pretreated group. The protein level of SIRT1 in SIRT1 siRNA cells was significantly decreased, confirming the success of the construction of SIRT1 siRNA (Figure 5A; File S1). The levels of phosphorylated ser27 site and ser47 site of SIRT1 protein were significantly decreased in the SiNPs plus SIRT1 siRNA group compared to the SiNP-treated group (*p* < 0.05). Similarly, the transcription factor HSF1 showed a significant downregulation after SIRT1 knockdown (*p* < 0.05). In addition, the distribution of HSF1 in BEAS-2B cells observed by immunofluorescence showed that the HSF1 protein in the SiNPs plus SIRT1 siRNA group was more evenly dispersed in the cell cytoplasm compared to the SiNPs group, suggesting a decrease in the nuclear transcriptional activity of the transcription factor HSF1, and the experimental results are shown in Figure 5B and File S1. The above results indicated that knockdown of SIRT1 not only resulted in the downregulation of SIRT1 activity but also downregulated the HSF1 protein and nuclear transcriptional activity of HSF1.

### 3.6. HSF1 siRNA Alleviated EMT Induced by SiNPs in BEAS-2B Cells

**Figure 6:**
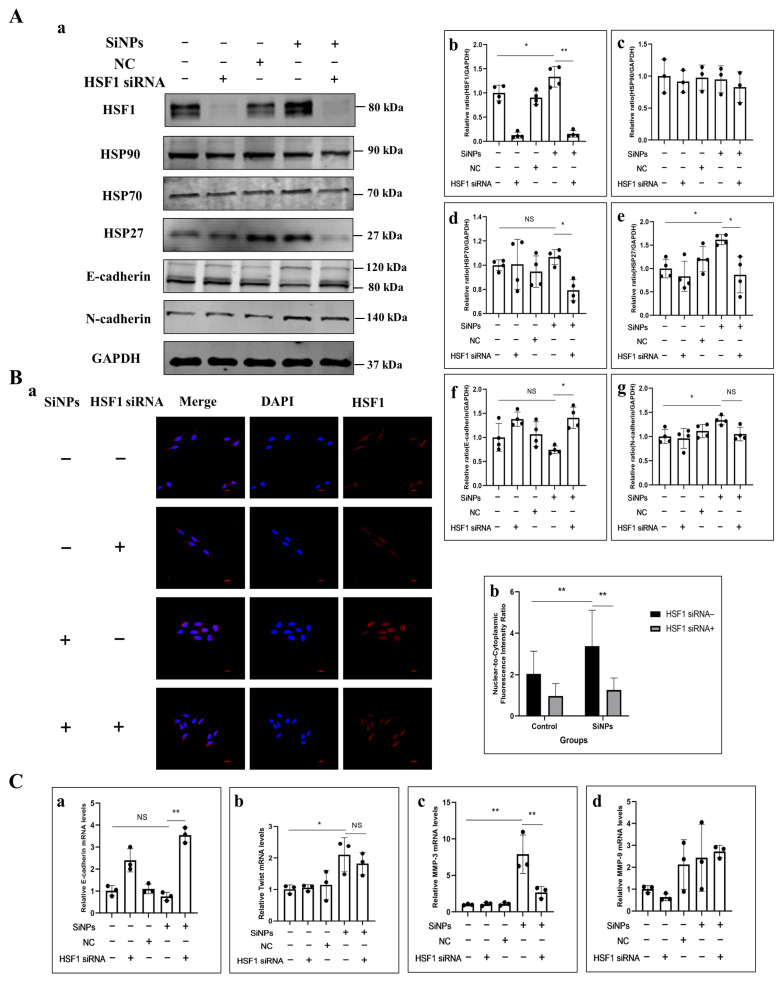
Effect of SiNPs on the HSF1/HSPs signaling pathway in BEAS-2B cells with HSF1 knockdown through HSF1 siRNA. (**A**) Effect of HSF1 knockdown on HSF1, HSP90, HSP70, HSP27, E-cadherin, and N-cadherin in BEAS-2B cells before SiNPs (10 μg/mL) exposure by Western blot. (**a**) Representative Western blot pictures. (**b**–**g**) Quantitative analysis of the grayscale values of the above proteins by ImageJ, *n* = 4, and the data were expressed as mean ± S.D. Non-targeting siRNA was used as a negative control (NC). (**B**) Effect of HSF1 knockdown on the distribution and quantitative analysis of HSF1 in BEAS-2B cells. (**a**) Representative immunofluorescence images of HSF1 proteins in BEAS-2B cells. (**b**) Quantitative analysis of the nuclear-cytoplasmic ratio of HSF1 protein in BEAS-2B cells. Blue: DAPI; red: HSF1, scale bar: 20 μm. (**C**) Effect of HSF1 knockdown on EMT-related genes after SiNPs treatment. (**a**–**d**) E-cadherin, Twist, MMP-3, and MMP-9. * *p* < 0.05 and ** *p* < 0.01. SiNP-treated group compared with corresponding SiNPs plus HSF1 siRNA group or SiNP-treated group without HSF1 compared with the control group without HSF1 siRNA.

To verify whether SiNPs induced EMT through the HSF1/HSPs signaling pathway, HSF1 was knocked down using 50 µM HSF1 siRNA in pretreatment, and then, the protein expression of the HSF1/HSPs signaling pathway was detected by Western blot. The experimental groups were set as control group, 50 μM HSF1 siRNA pretreated group, NC group, 10 μg/mL SiNPs treated group, and 10 μg/mL SiNPs plus 50 μM HSF1 siRNA pretreated group. As shown in Figure 6A and File S1, the protein expression of HSF1 in the SiNP-treated group increased significantly, whereas the protein content of HSF1 in the SiNPs plus HSF1 siRNA group decreased significantly (*p* < 0.05), which indicated that the knockdown of HSF1 siRNA in BEAS-2B cells was successfully constructed. Similarly, the expression of heat shock protein HSP27 was significantly upregulated in the SiNP-treated group (*p* < 0.05), but the protein expression of HSP70 and HSP27 was downregulated in the SiNPs plus HSF1 siRNA group. However, there was no statistically significant difference in the expression level of HSP90 in the comparison between the groups. These results indicated that HSF1 siRNA could effectively inhibit the expression of heat shock proteins HSP70 and HSP27 by inhibiting the transcription factor HSF1. Additionally, in the SiNPs plus HSF1 siRNA group, the level of E-cadherin protein increased while N-cadherin protein expression decreased compared to the SiNP-treated group. When the HSF1 expression was reduced, the change in EMT-related E-cadherin gene increased, and the MMP-3 gene decreased compared to the SiNP-treated group. In addition, the distribution of HSF1 in the cells was detected by immunofluorescence. As shown in Figure 6B and File S1, after SiNPs treatment, HSF1 was almost distributed in the nucleus, indicating the nuclear localization of HSF1. However, in the SiNP-treated group pretreated with HSF1 siRNA, a significant decrease of HSF1 in the nucleus was observed, which was almost restored to the state of the control group. The results indicated that HSF1 siRNA could affect HSF1 protein expression and reduce its nuclear transcriptional activity by decreasing the HSF1 mRNA levels. The above results demonstrated that SiNPs could further induce the expression of HSP27- and EMT-related genes by upregulating the transcription factor HSF1 and enhancing its transcriptional activity, thereby inducing EMT.

## 4. Discussion

SiNPs are an inorganic material with extensive industrial and biomedical applications, which have raised concerns about public health around the world. Previous studies have demonstrated that SiNPs induced ER stress in different cell lines [12,37,38] and EMT in bronchial epithelial cells [15]. However, the specific mechanisms remain to be fully understood. This study was designed to examine SiNP-induced EMT of bronchial epithelial cells and its regulatory mechanism. In the domain of nanotoxicology, it was a widely acknowledged fact that nanoparticle size was a pivotal factor in determining their biological effects [39]. In previous studies, SiNPs of different sizes were evaluated, including smaller sizes such as ~19 nm [40]. However, it was found that SiNPs of smaller sizes (especially those measuring less than 20 nm) exhibited aggregation on occasion. This might introduce further factors affecting cellular uptake and interaction patterns with the particles. These factors, in turn, influenced the interpretation of cellular responses to specific signaling pathways. Conversely, SiNPs in the 50–70 nm size range demonstrated exceptional monodispersity and stability under experimental conditions, while smaller sizes exhibited augmented toxic effects [20]. This provided a more reliable and consistent model system for in-depth investigation of their regulatory mechanisms on cellular signaling pathways. Consequently, this study selected SiNPs of a single size as the toxic substance for utilization in experimentation.

It was reported that ER stress was associated with the development and progression of lung cancer [41,42]. BEAS-2B cells are a cell line that has been extensively utilized in research involving lung epithelial cell biology, toxicology, and pharmacology [43,44,45,46]. Furthermore, the use of BEAS-2B cells in the malignant transformation in vitro provides an ideal model with which to investigate the effects of environmental pollutants on lung cancer and its pathogenesis [47]. Consequently, a comprehensive investigation into the effect of SiNP-induced ER stress on BEAS-2B cells migration is needed, which may prove crucial in elucidating the underlying mechanisms of lung diseases. In the present study, we used TEM to investigate the effect of SiNPs on the structure of the ER in BEAS-2B cells. The results demonstrated that internalized SiNPs accumulated in the ER, leading to rough ER degranulation and structural disruptions (Figure 2A; File S1), which affected the ultrastructure of the ER and the function of synthesized proteins. It has been demonstrated that, when SiNPs entered the ER, the ER could be structurally damaged by spacing expansion, ribosome detachment, and ER swelling and fracture in HUVECs [48]. In eukaryotic cells, the ER is responsible for the folding and maturation of approximately one-third of cellular proteins, which are subsequently transported to their respective locations to perform their function [42]. The ER is highly susceptible to various stressors, including metabolic disturbances, inflammatory responses, and oxidative stress. Disruption of ER homeostasis can lead to the induction of ER stress. In circumstances of ER stress, the UPR can be activated by three major classical ER stress membrane proteins, namely inositol-requiring enzyme 1α (IRE1α), protein kinase R-like ER kinase (PERK), and activating transcription factor 6 (ATF6) [49,50]. These three critical transmembrane proteins generally bind to the molecular chaperone glucose-regulated protein 78 (GRP78, also known as BIP). However, when cells undergo ER stress, these three transmembrane proteins dissociate from BIP and protect the cells by inducing BIP to promote protein folding and prevent the accumulation of misfolded proteins [51]. Moreover, PERK phosphorylates eukaryotic translation initiation factor 2α (eIF2α) in order to inhibit protein translation. Phosphorylated eIF2α (P-eIF2α) has been observed to induce the translation of ATF4, thereby promoting the expression of CHOP [52]. It has been demonstrated by previous studies that SiNPs selectively activate ER stress in hepatocytes by activating the EIF2AK3 and ATF6 pathways. Furthermore, studies have indicated that ROS induced by NPs can regulate ER stress through the IRE1-JNK pathway and PERK pathway under both in vivo and in vitro conditions [53]. In this study, the changes in ER stress-related proteins were evaluated after SiNPs treatment in BEAS-2B cells. The results demonstrated that the protein levels of BIP and CHOP were significantly elevated, suggesting the possibility of SiNPs entering cells and thereby inducing further ER stress. In order to verify the activation of the ER stress response by SiNPs, cells exposed to SiNPs were pretreated with the ER stress inhibitor 4-PBA for 2 h. It was evident that 4-PBA inhibited the protein expression levels of CHOP and BIP that had been induced by SiNPs. However, further investigation is required to elucidate the specific pathways involved in SiNP-induced ER stress.

Next, we tried to explore how the activation of ER stress regulates downstream signaling molecules. It has been shown that SIRT1 could act as a negative regulator of ER stress response and attenuate ER stress in vitro and in vivo [54,55]. However, the effect of ER stress on SIRT1 remains to be fully clarified. In this study, we demonstrated that ER stress under short-term exposure to SiNPs had no effect on the total SIRT1 protein levels (Figure 2B; File S1). SIRT1 expression is regulated through complex mechanisms, including transcription, post-transcription, and post-translation [56]. Regarding post-translation, SIRT1 levels and activity are regulated by sumoylation and phosphorylation [56,57]. It has been reported that more than 10 residues of SIRT1 undergo phosphorylation or dephosphorylation in various cell types under different conditions [58]. Among them, ser27 and ser47 in human SIRT1 have received the most extensive study, so we investigated whether the increased activation of ER stress by SiNPs is mediated by increased SIRT1 activity through phosphorylation of SIRT1 at the ser27 and ser47 sites. As shown in Figure 2B and File S1, SiNPs increased the phosphorylation of SIRT1 at the ser27 and ser47 sites. As a low molecular weight fatty acid, 4-PBA has been proven to effectively inhibit ER stress in experimental models. This study demonstrated that 4-PBA, at its most effective dose of 5 mM, further reversed the activation of the SIRT1 (ser47) and the HSF1 transcription factors by decreasing the protein expression levels of BIP and CHOP in BEAS-2B cells. Interestingly, 4-PBA treatment increased the phosphorylation of SIRT1 at the ser27 site. SIRT1 phosphorylation has been demonstrated to enhance the catalytic activity of SIRT1 [59,60]. The phosphorylation of SIRT1 at the Ser27 and Ser47 sites can be regulated by Ca^2+^/calmodulin-dependent protein kinase kinase (CaMKK)β, thereby increasing the stability and activity of SIRT1 [61]. Human SIRT1 is phosphorylated at Ser27, Ser47, and Thr530 by JNK1, and SIRT1 phosphorylation increases its nuclear localization and enzymatic activity [62]. JNK-mediated phosphorylation of SIRT1 at the Ser27 residue has been shown to induce its stability, followed by deacetylation-dependent nuclear translocation of Snail [63]. Furthermore, studies have demonstrated that ER stress-induced SIRT1 requires the PI3K-Akt-GSK3β signaling pathway [64]. The study indicated that SiNPs could induce ER stress and lead to phosphorylation of SIRT1 Ser27/47 sites. This finding further indicated that SiNPs might activate the JNK/AKT pathway through ER stress, thereby influencing the modification of SIRT1 phosphorylation sites. However, further research is needed to validate this hypothesis. With regards to the contradictory results regarding SIRT1 phosphorylation sites (a decrease in p-Ser47 vs. an increase in p-Ser27), it was observed that 4-PBA treatment led to a decrease in SIRT1 p-Ser47 levels, while the p-Ser27 levels showed an upward trend. This finding was of interest and should be explored further. At present, based on our existing experimental studies, we were unable to fully elucidate the exact molecular mechanisms underlying this discrepancy. Following a thorough review of the relevant literature, the following possibilities were identified. The regulation of SIRT1 phosphorylation is a complex process. SIRT1 activity is subject to rigorous regulation by phosphorylation at multiple sites, with each site potentially exhibiting a distinct function and, in some cases, an antagonistic effect. It is possible that Ser47 and Ser27 are regulated by different kinases/phosphatases and that their responses to ER stress inhibitors might differ. Furthermore, there may be feedback regulation or compensatory mechanisms in place. Inhibiting ER stress with 4-PBA may trigger feedback regulation or compensatory signaling pathways within cells, leading to differential regulation of SIRT1 at different sites by specific kinases/phosphatases. This result suggested that the post-translational modifications of SIRT1 (especially phosphorylation at different sites) in response to ER stress regulation warrant further investigation.

SIRT1, a member of the sirtuin family, is a stress-responsive protein deacetylase [65]. Activation of SIRT1 has been reported to promote the deacetylation of HSF1, a stress-inducible transcription factor that binds to the promoter of the HSP gene to regulate its transcription in response to a variety of stresses. Deacetylation of HSF1 by SIRT1 has been shown to enhance its binding to the promoter of the HSPs gene, thereby increasing HSPs expression [24,66]. In response to ER stress, SIRT1 activated HSF1 through deacetylation, thereby upregulating the expression of HSPs and enhancing cellular anti-stress and anti-apoptotic capabilities [24]. In the previous study, we found that SiNPs induced increased mRNA transcript levels of HSPs in BEAS-2B cells, which further impaired toxic effects in the lung. Consequently, we hypothesized that the SIRT1/HSF1/HSP signaling pathway might play an important role in SiNP-induced EMT. To further validate the deacetylase activity of SIRT1, we determined the acetylation level of the transcription factor HSF1 and showed that HSF1 acetylation decreased in a dose-dependent manner following SiNPs treatment. Previous studies have established that SIRT1 exerts its influence on HSF1 acetylation by deacetylating HSF1 at lysine, thereby delaying HSF1 decay and potentially leading to the gradual upregulation of the total HSF1 protein levels. Under stress conditions, HSF1 is activated, translocates to the nucleus, and binds to the promoter regions of HSP genes to repair or degrade aberrant proteins and protect cells from damage. In this study, we found that the nuclear transcriptional activity of HSF1 exhibited a SiNPs dose dependence (Figure 2D). To further strengthen the regulatory role of SIRT1 on HSF1, we knocked down SIRT1 using SIRT1 siRNA and found that SIRT1 phosphorylation appeared to be downregulated and further affected HSF1 protein and nuclear transcriptional activity (Figure 5).

Increasing evidence indicated that HSF1 is overexpressed in various types of human cancers, further enhancing cancer cell EMT, and is associated with cancer invasiveness [28,67,68]. The preceding study revealed that SiNPs significantly affect the expression of HSPs in BEAS-2B cells, thereby further regulating the lung toxicity induced by SiNPs [20]. HSF1 is recognized as the main transcription factor of HSR [69]. It regulates the expression of HSPs and further coordinates cell survival in response to different forms of cellular stress [70,71]. Among the heat shock proteins, HSP27, HSP70, and HSP90 are the most extensively studied stress-inducible HSPs [72], which are induced in response to a wide range of physiological and environmental insults, thereby enabling cells to survive lethal conditions. In our study, we observed that SiNPs mainly upregulated the expression of HSP70 and HSP27 molecular chaperones but did not affect the expression of HSP90, suggesting that HSP70 and HSP27 might play a critical role in the heat shock response induced by SiNPs (Figure 4A; File S1). Since we found that SiNPs induced EMT in BEAS-2B cells in the previous period, the function of HSPs in EMT in lung epithelial cells remains unknown. Subsequently, the expression levels of E-cadherin and N-cadherin, two typical EMT markers, were measured, and it was found that E-cadherin was downregulated in a dose-dependent manner, whereas N-cadherin was upregulated. In addition, the transcription factors that mediate the activation of EMT-related phenotypic transitions were examined, and it was found that the mRNA level of E-cadherin was decreased after treatment with SiNPs, whereas the mRNA expression of Twist, MMP-9, and MMP-3 was strongly increased. It has been shown that knockdown of Hsp70 decreased the cell migration ability, as well as the mRNA levels, of Slug, Snail, and Twist in EMT, and it was also found that inhibition of HSF1 decreased the level of HSP70, further inducing the development of EMT [73]. Downregulation of HSP70 expression has also been shown to increase the E-cadherin/N-cadherin ratio and inhibit the proliferation, migration, and invasion of gastric cancer cells [74]. Hsp70 silencing resulted in a severe loss of cell motility, an inability to form calmodulin-conjugated protein complexes, and stimulated their separation from neighboring cells [75]. In the present study, it was revealed that HSP70 might affect E-cadherin and N-cadherin, further leading to EMT in BEAS-2B cells. HSP27, a small ATP-independent chaperone, is overexpressed under conditions of cellular stress and protects proteins from unfolding, thereby promoting protein homeostasis and cell survival. Previous studies have shown that HSP27 regulates the STAT6/Twist signaling pathway to achieve IL-6-mediated EMT, and its attenuation reversed EMT and reduced cell migration, invasion, and matrix metalloproteinase activity [76]. It was also found that HSP27 induced regulation of the β-catenin/MMP-3 signaling cascade response, which is a key pathway for EMT [77]. Similar to our results, SiNPs upregulated the mRNA levels of Twist, MMP-9, and MMP-3 and induced the upregulation of EMT-related proteins. This finding is in accordance with our previous study [15]. In addition, knockdown of HSF1 in SiNP-treated cells only inhibited the mRNA expression of MMP-3, suggesting that the upregulation of HSP27 by HSF1 is likely to be involved in the HSP27-induced β-catenin/MMP-3 signaling cascade response in regulating EMT. This study is the first to explore the mechanism by which SiNPs regulated the HSF1/HSPs signaling pathway through ER stress, ultimately leading to EMT in BEAS-2B cells. However, it should be noted that the conclusions drawn from this in vitro model have certain limitations, especially in terms of simulating the complex microenvironment of lung tissue and systemic responses in vivo. In order to surmount these limitations and to more reliably validate the key findings of this study, long-term in vitro and in vivo research models would be established for further exploration.

## 5. Conclusions

**Figure 7:**
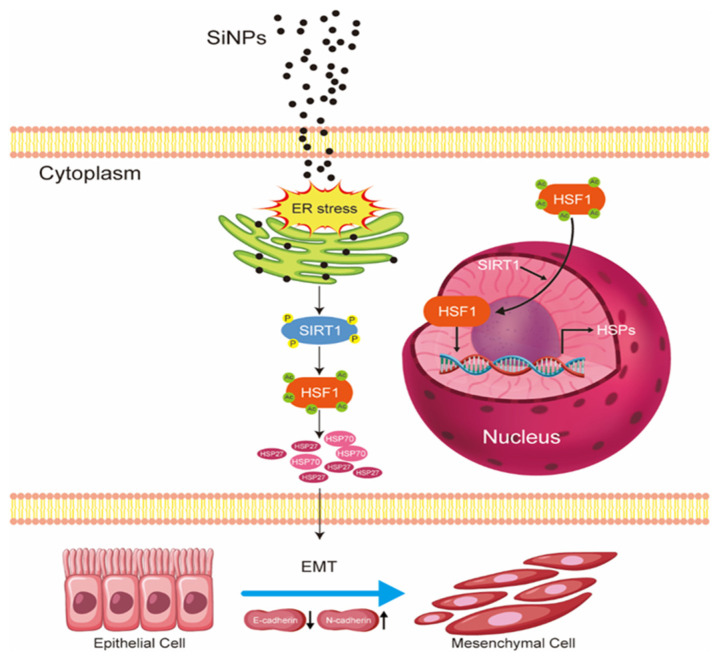
The schematic model illustrated the underlying mechanism of EMT induced by SiNPs. Following the internalization of SiNPs into BEAS-2B cells, SiNPs accumulated in the ER and further induced ER stress. This ER stress subsequently led to the phosphorylation and activation of SIRT1, which functioned as a deacetylase. SIRT1 deacetylation was found to modulate HSF1, resulting in its nuclear translocation and subsequent transcriptional upregulation of HSP70 and HSP27 expression. This series of events serves to enhance the HSR- and EMT-related genes, consequently inducing the onset of EMT.

In summary, the existing study has shown that exposure to SiNPs triggered ER stress, which further activated the phosphorylation of SIRT1, upregulating downstream HSF1 expression. Subsequently, HSF1 underwent nucleus-directed translocation and positively regulated the protein expressions of HSP70 and HSP27 as a transcription factor, which further influenced EMT-related genes and ultimately led to EMT in BEAS-2B cells (Figure 7). These findings reveal for the first time the important role of ER stress activation in further regulating the SIRT1/HSF1/HSPs signaling pathway in the SiNP-induced EMT process of BEAS-2B cells and provide new insights and molecular targets for future studies on SiNP-induced EMT and even lung cancer.

## Figures and Tables

**Table 1:** Table of primer sequences.

Gene Name	Forward Sequence (5′-3′)	Reverse Sequence (5′-3′)
MMP-3	TGGATTGGAGGTGACGGGGAAG	ATGCCAGGAAAGGTTCTGAAGTGAC
MMP-9	AGTCCACCCTTGTGCTCTTCCC	TCTCTGCCACCCGAGTGTAACC
Twist	CCTCGGACAAGCTGAGCAAGATTC	GTCGCTCTGGAGGACCTGGTAG
E-cadherin	AGTCACTGACACCAACGATAAT	ATCGTTGTTCACTGGATTTGTG
GAPDH	CAGGAGGCATTGCTGATGAT	GAAGGCTGGGGCTCATTT

## Data Availability

The original contributions presented in this study are included in the article/Supplementary Material. Further inquiries can be directed to the corresponding author.

## Supplementary Materials

The following supporting information can be downloaded at: https://www.mdpi.com/article/10.3390/jox15050137/s1, Supplementary File S1, list of original images.

## Abbreviations

The following abbreviations are used in this manuscript:

SiNPs	Amorphous silica nanoparticles
OECD	Organization for Economic Co-operation and Development
SiO_2_	Silicon dioxide
EMT	Epithelial–mesenchymal transition
HSPs	Heat shock proteins
ER	Endoplasmic reticulum
UPR	Unfolded protein response
HSF1	Heat shock factor 1
siRNA	small interfering RNA
TGF-β	Transforming growth factor-β
TEM	Transmission Electron Microscope
4-PBA	4-Phenylbutyric acid
