# Supplementary figures and images for: Silica Nanoparticles Induced Epithelial–Mesenchymal Transition in BEAS-2B Cells via ER Stress and SIRT1/HSF1/HSPs Signaling Pathway
Source: J Xenobiot. 2025 Aug 23;15(5):137. doi: 10.3390/jox15050137 (PMC12452771; doi:10.3390/jox15050137)

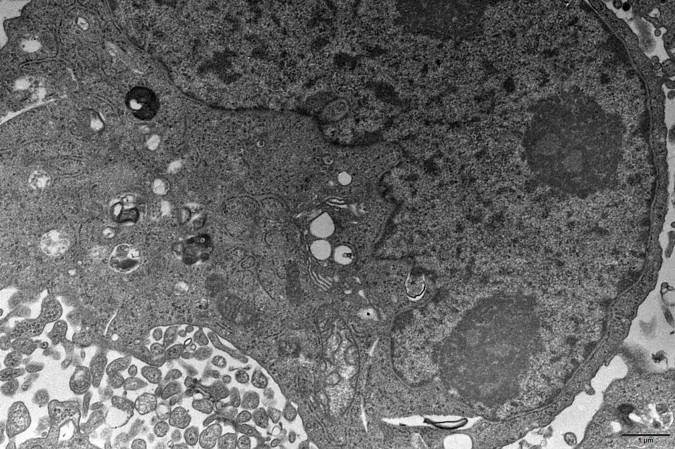

Supplement: Supplementary file 1 [file jox-15-00137-s001.zip › File S1/Figure 2 A) Control 2.jpg]

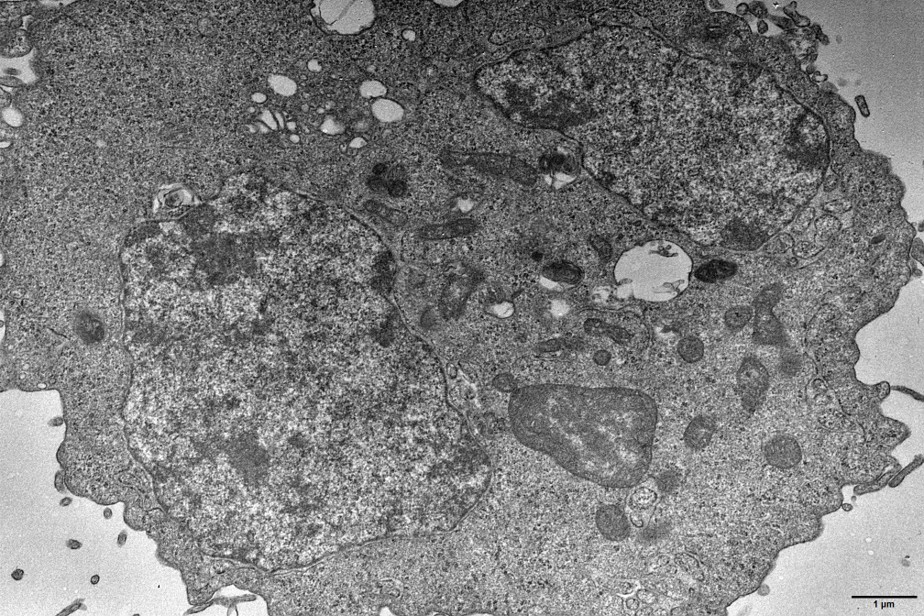

Supplement: Supplementary file 1 [file jox-15-00137-s001.zip › File S1/Figure 2 A) Control 3.jpg]

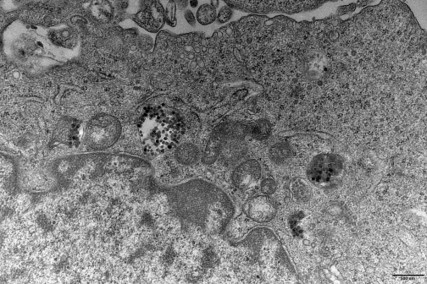

Supplement: Supplementary file 1 [file jox-15-00137-s001.zip › File S1/Figure 2 A) SiNPs 1.jpg]

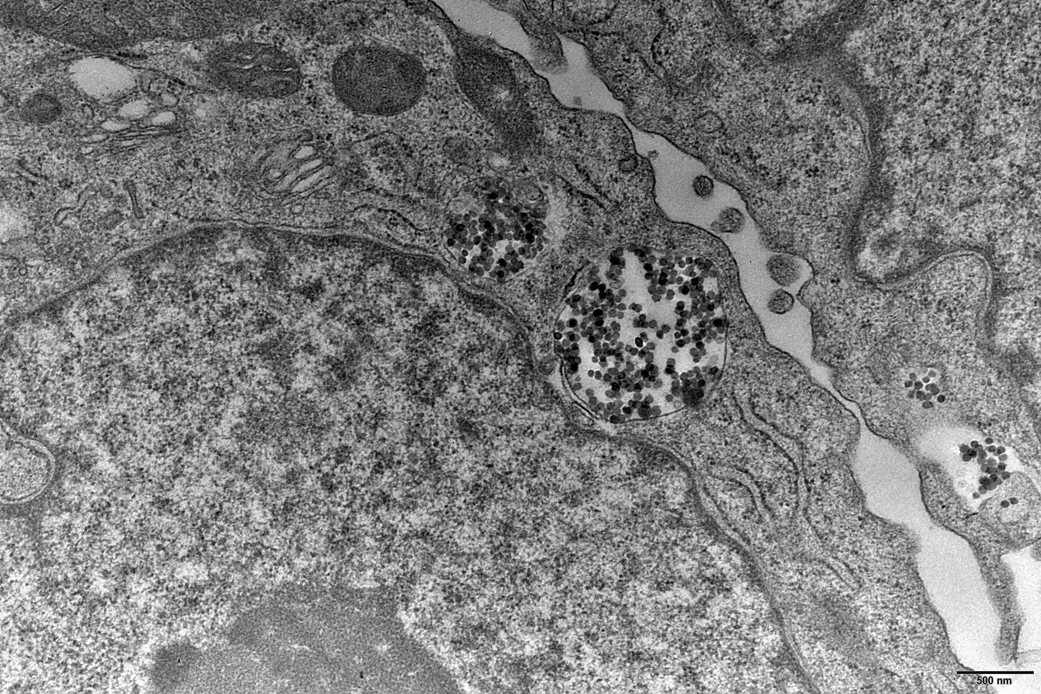

Supplement: Supplementary file 1 [file jox-15-00137-s001.zip › File S1/Figure 2 A) SiNPs 2.jpg]

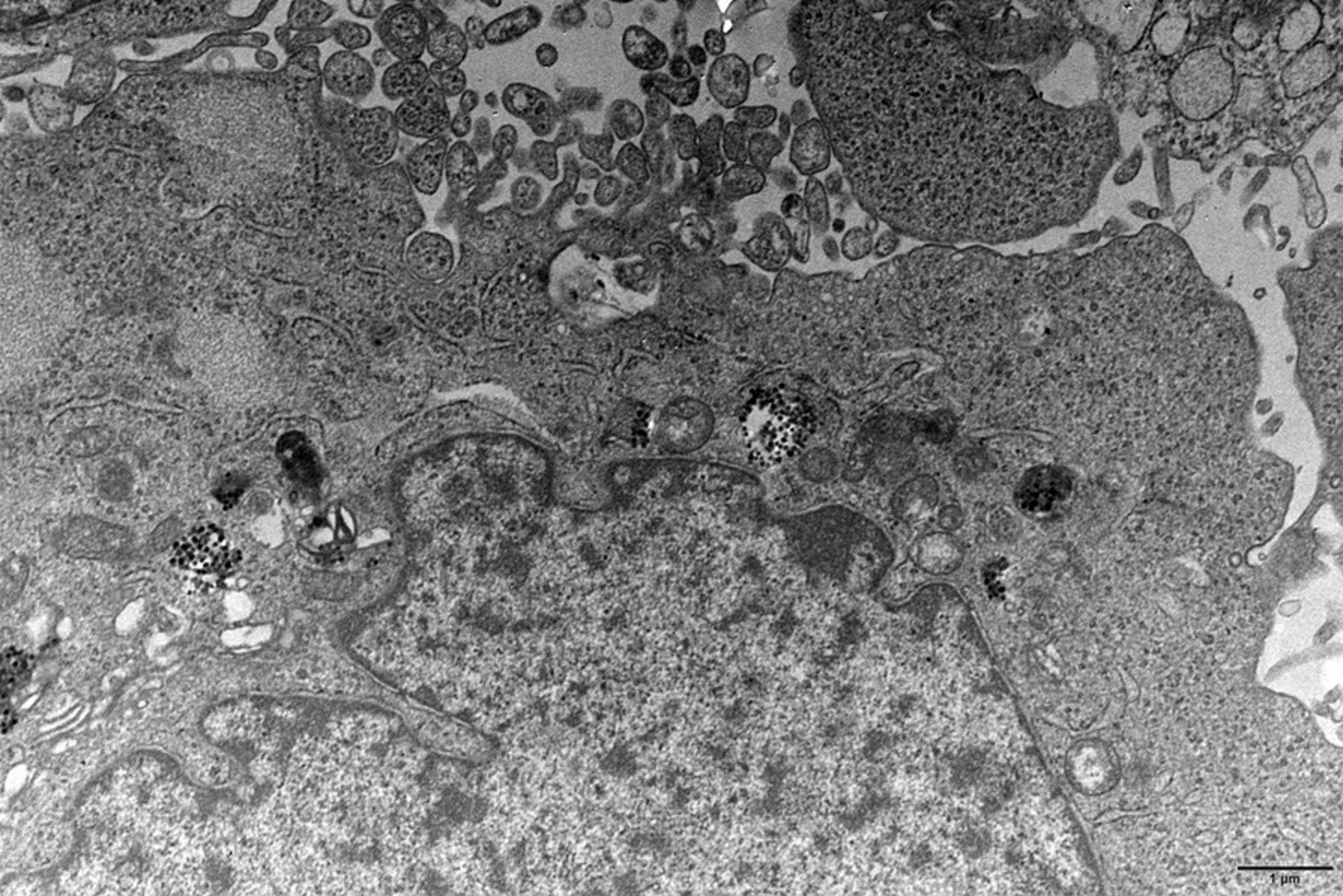

Supplement: Supplementary file 1 [file jox-15-00137-s001.zip › File S1/Figure 2 A) SiNPs 3.jpg]

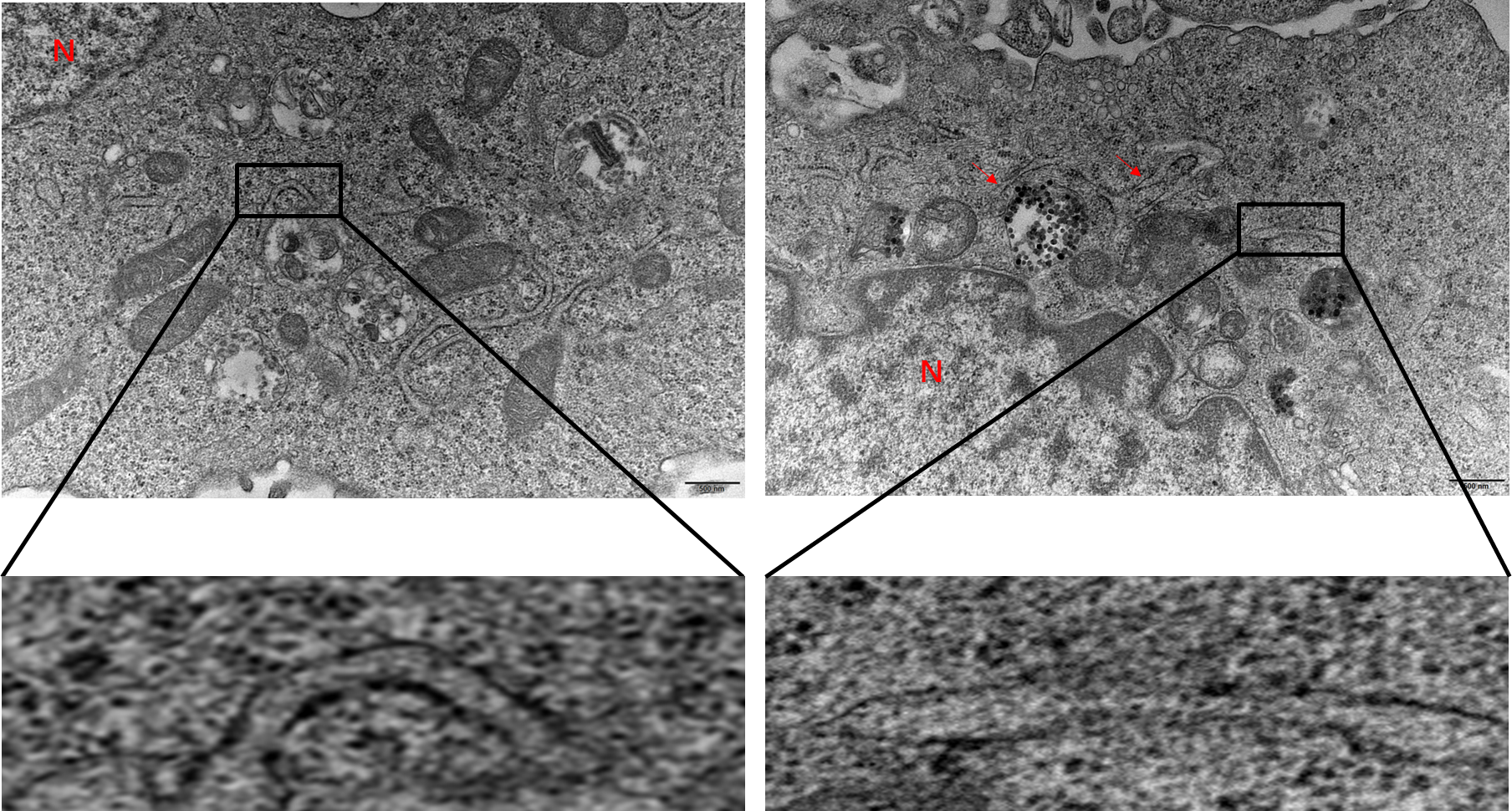

Supplement: Supplementary file 1 [file jox-15-00137-s001.zip › File S1/Figure 2 A).tif]

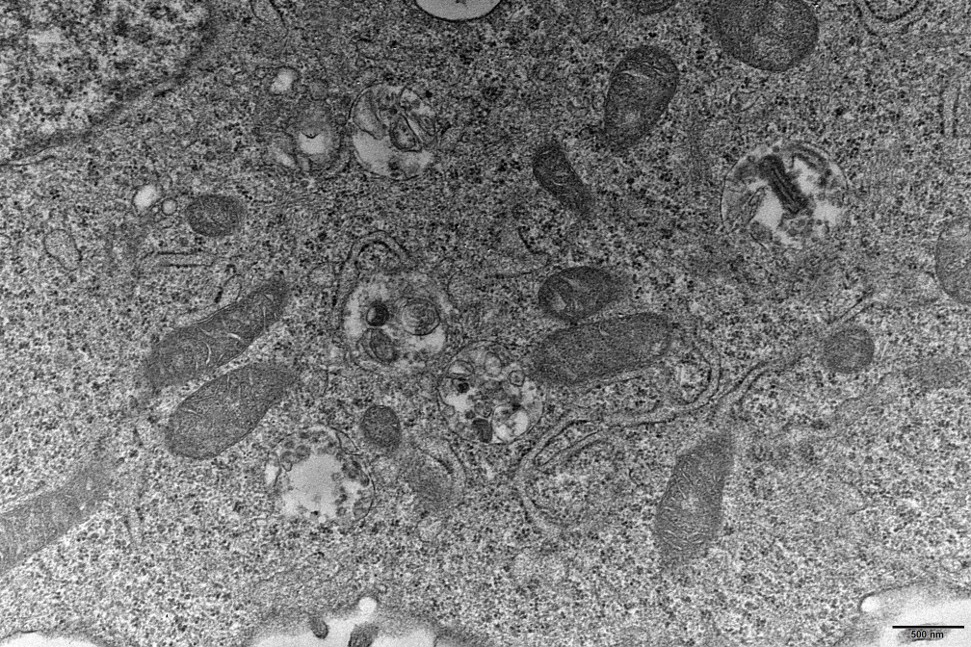

Supplement: Supplementary file 1 [file jox-15-00137-s001.zip › File S1/Figure 2 A) Control 1.jpg]

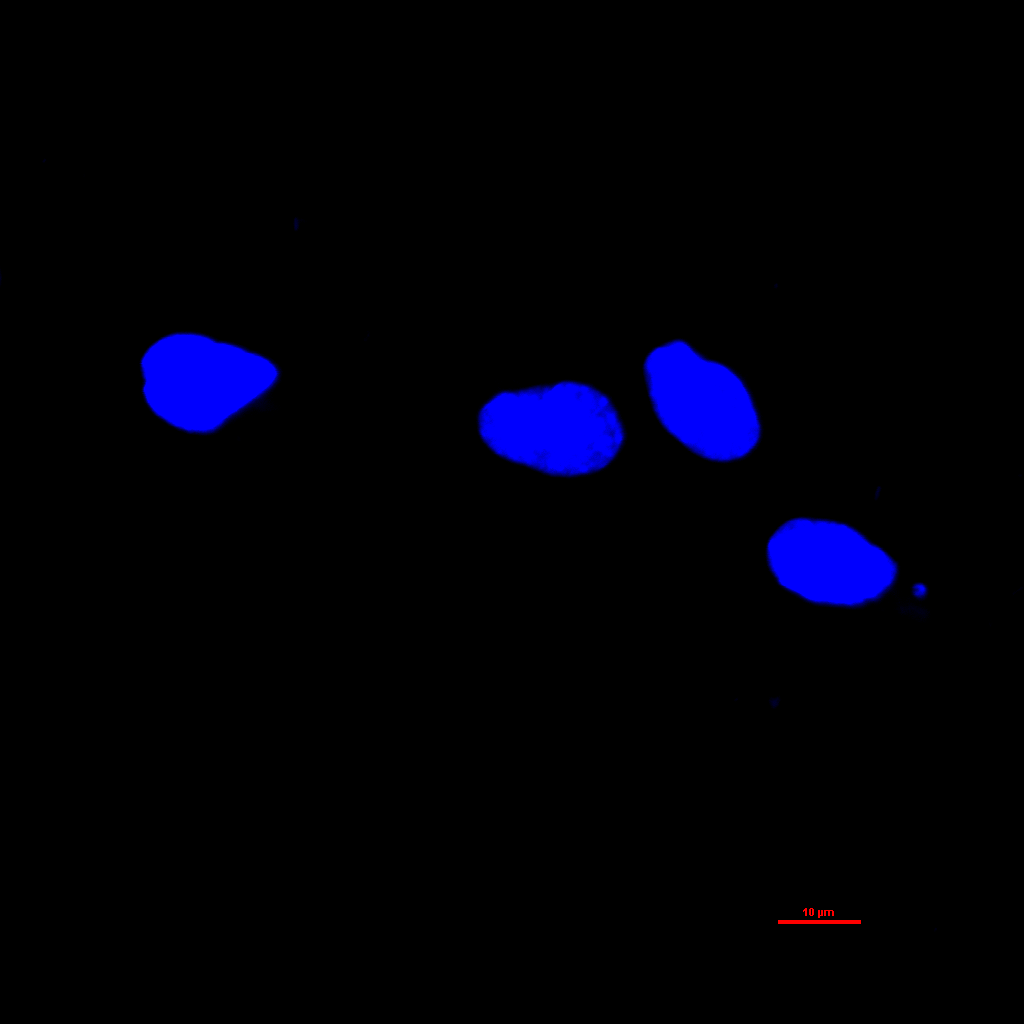

Supplement: Supplementary file 1 [file jox-15-00137-s001.zip › File S1/Figure 2 D) 0组 DAPI.tif]

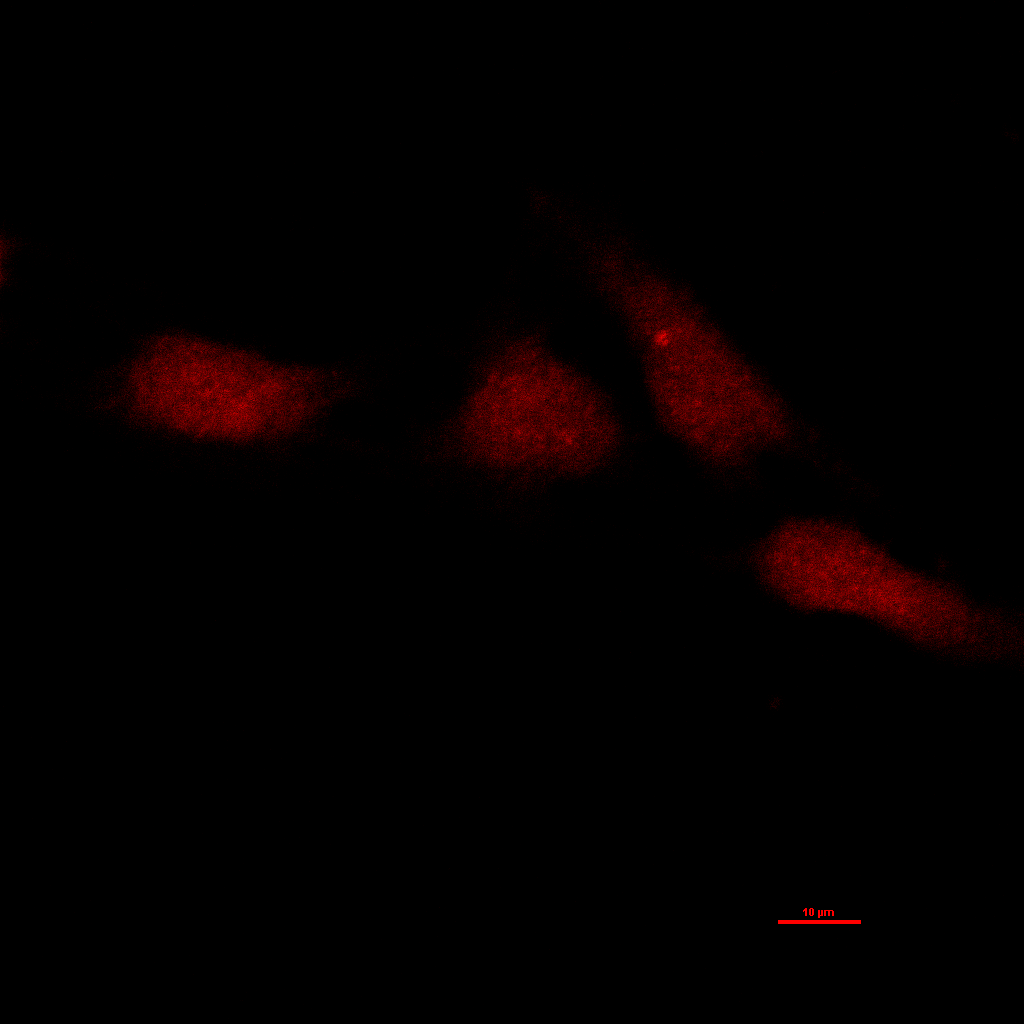

Supplement: Supplementary file 1 [file jox-15-00137-s001.zip › File S1/Figure 2 D) 0组 HSF1.tif]

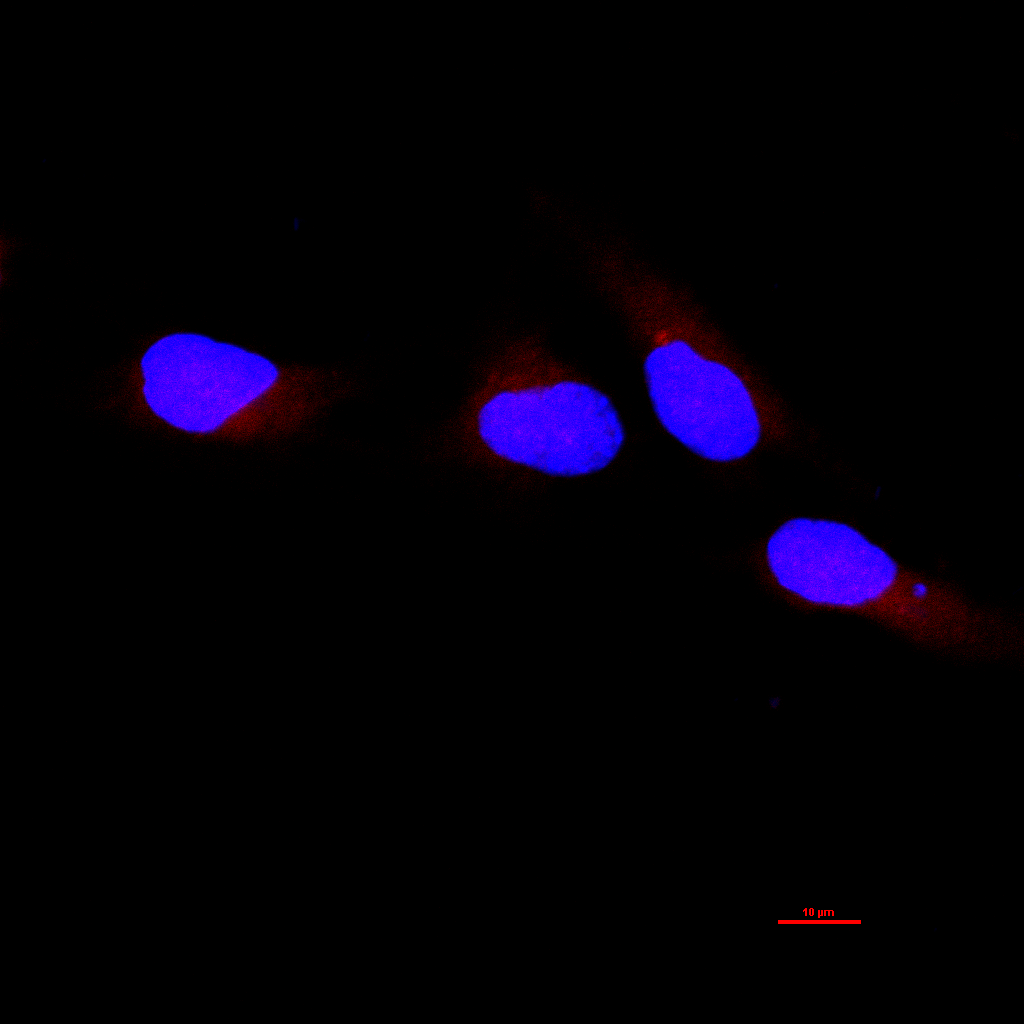

Supplement: Supplementary file 1 [file jox-15-00137-s001.zip › File S1/Figure 2 D) 0组 Merge.tif]

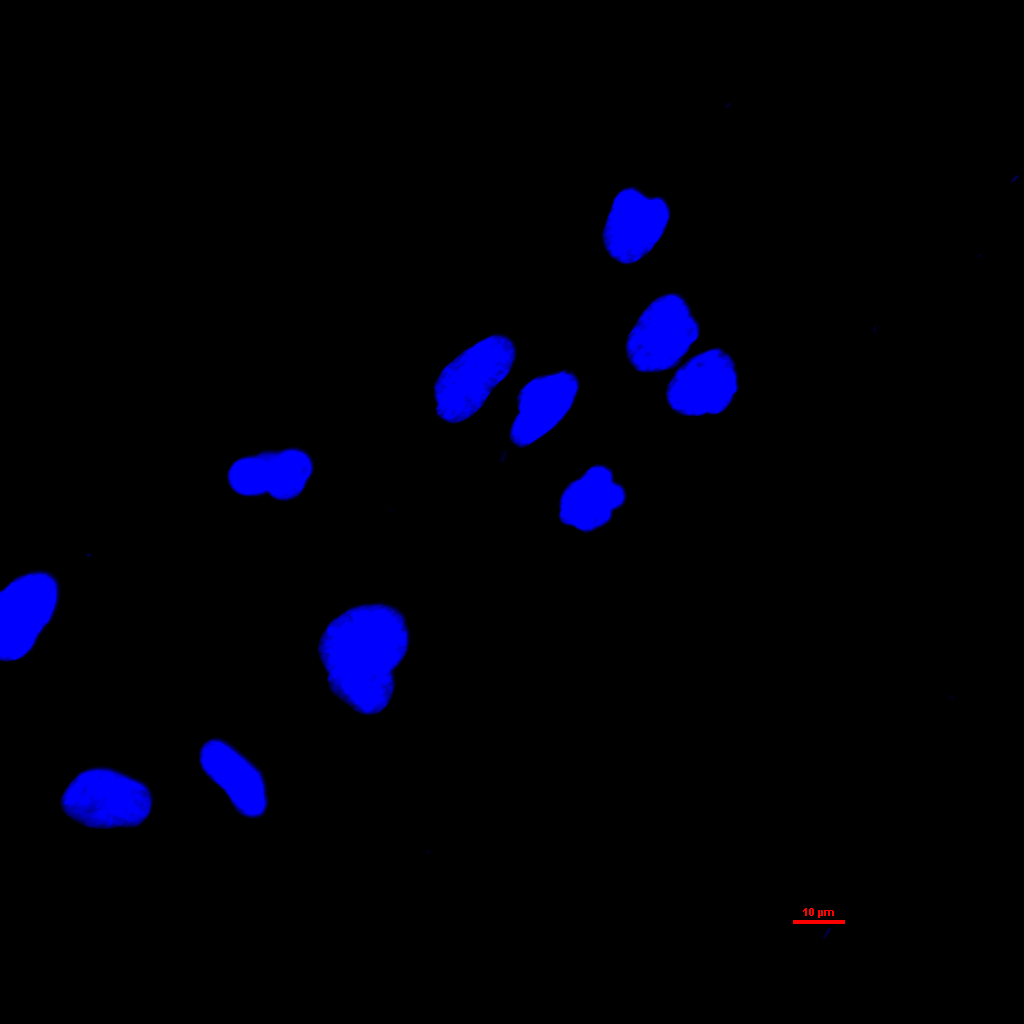

Supplement: Supplementary file 1 [file jox-15-00137-s001.zip › File S1/Figure 2 D) 10组 DAPI.tif]

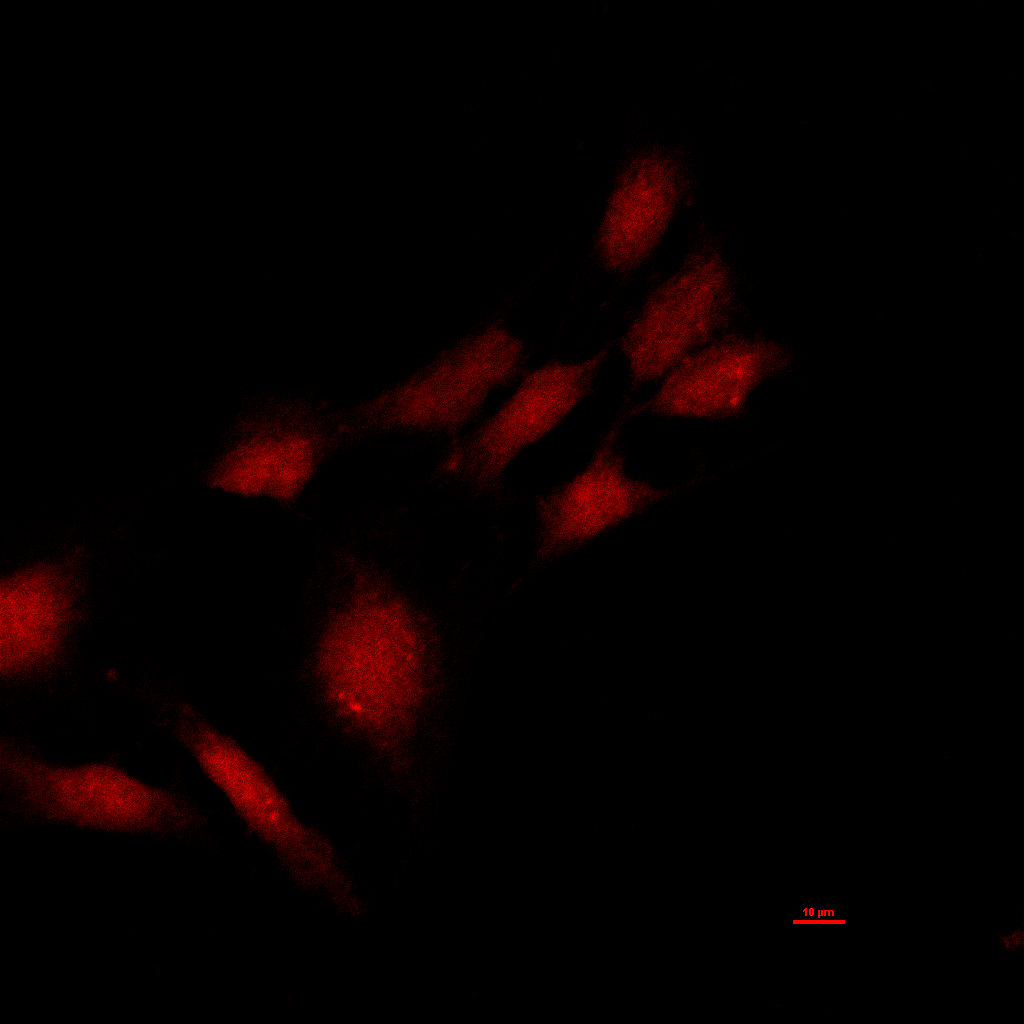

Supplement: Supplementary file 1 [file jox-15-00137-s001.zip › File S1/Figure 2 D) 10组 HSF1.tif]

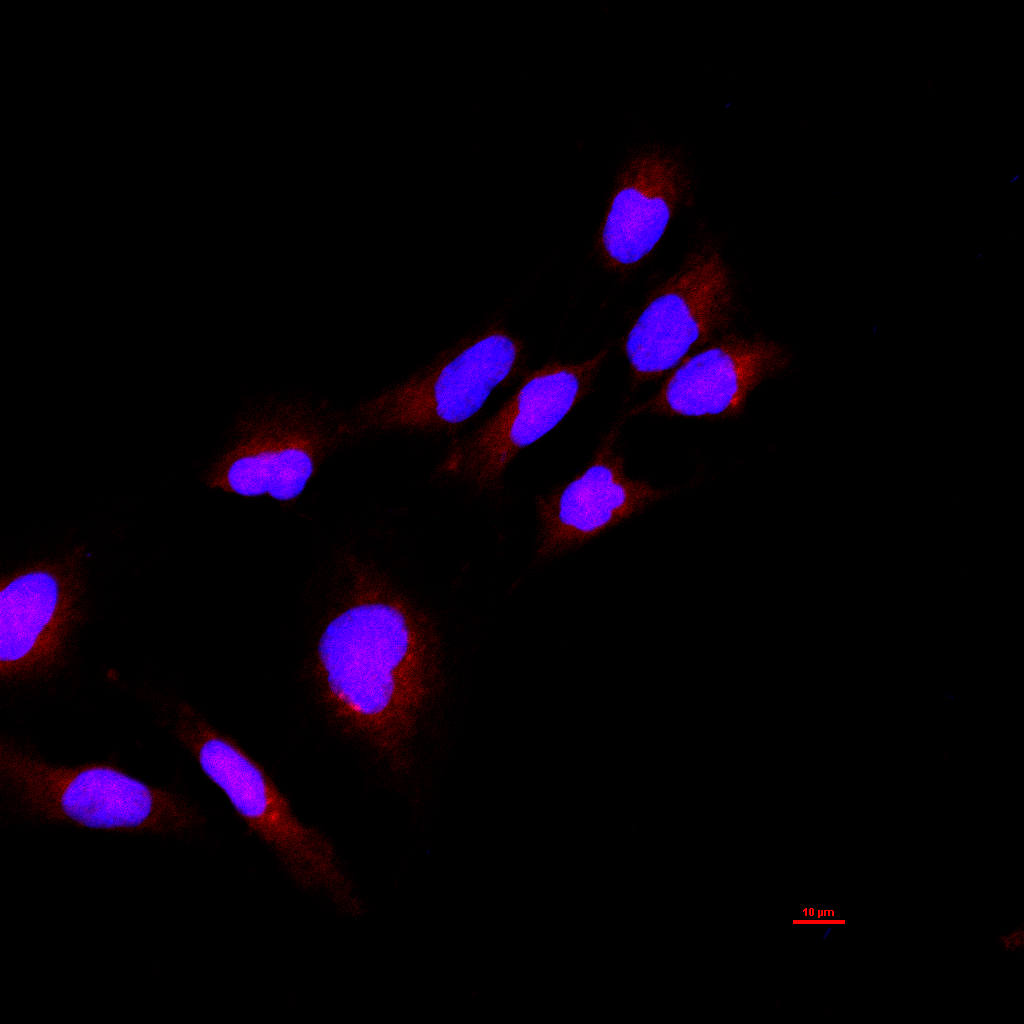

Supplement: Supplementary file 1 [file jox-15-00137-s001.zip › File S1/Figure 2 D) 10组 Merge.tif]

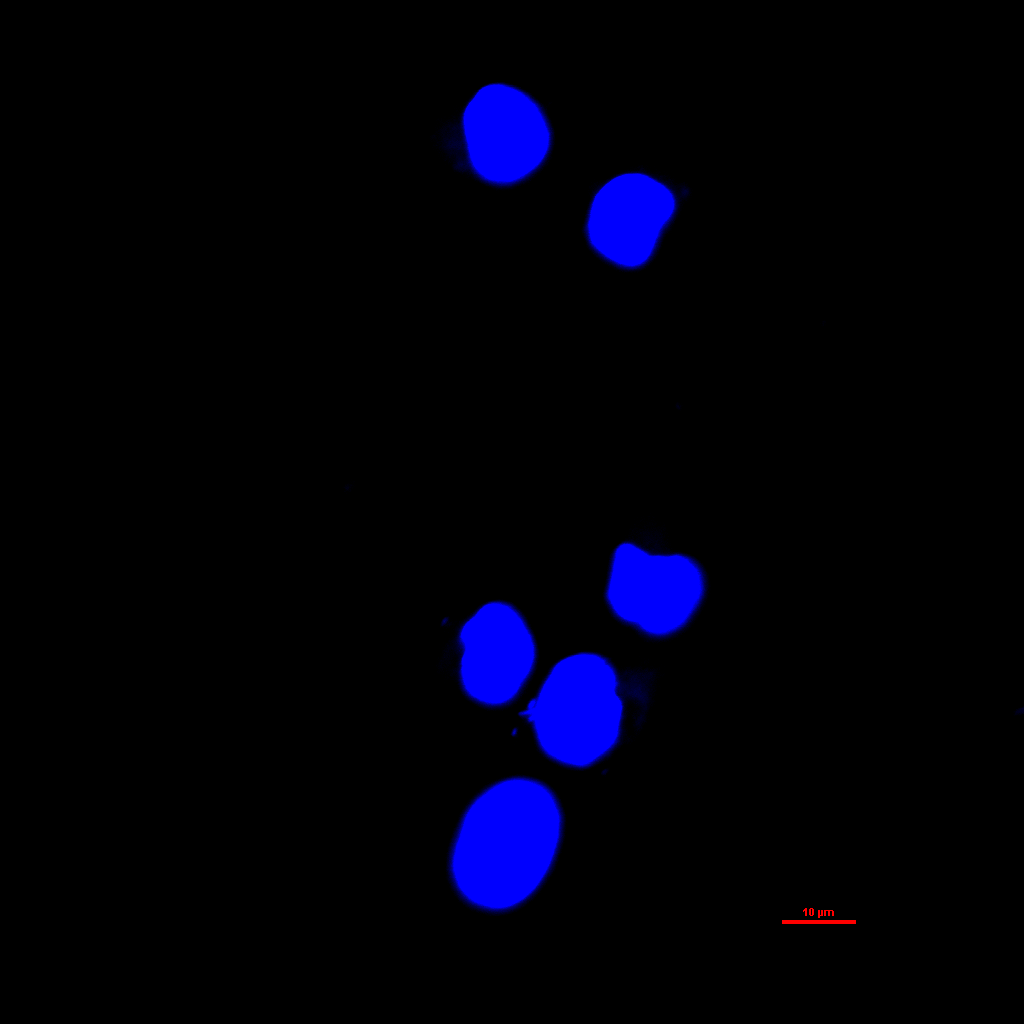

Supplement: Supplementary file 1 [file jox-15-00137-s001.zip › File S1/Figure 2 D) 20组 DAPI.tif]

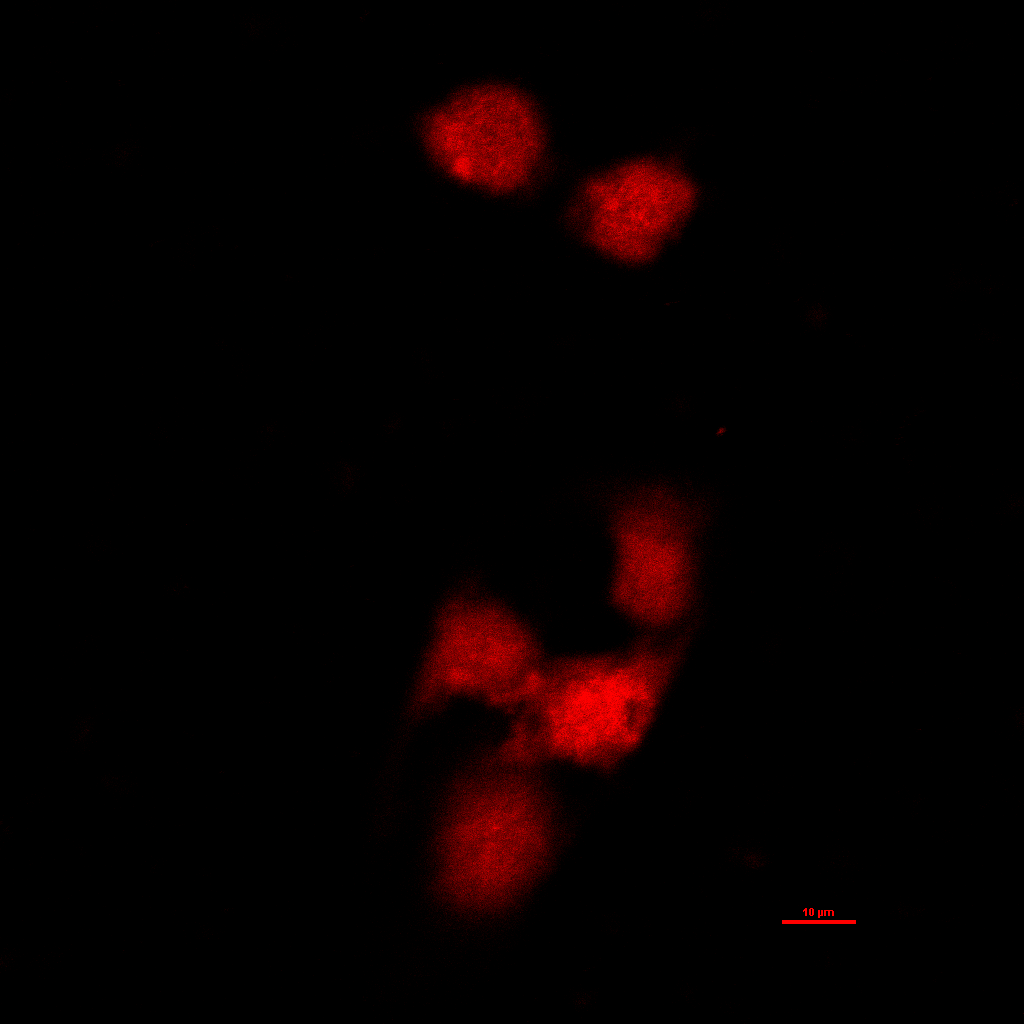

Supplement: Supplementary file 1 [file jox-15-00137-s001.zip › File S1/Figure 2 D) 20组 HSF1.tif]

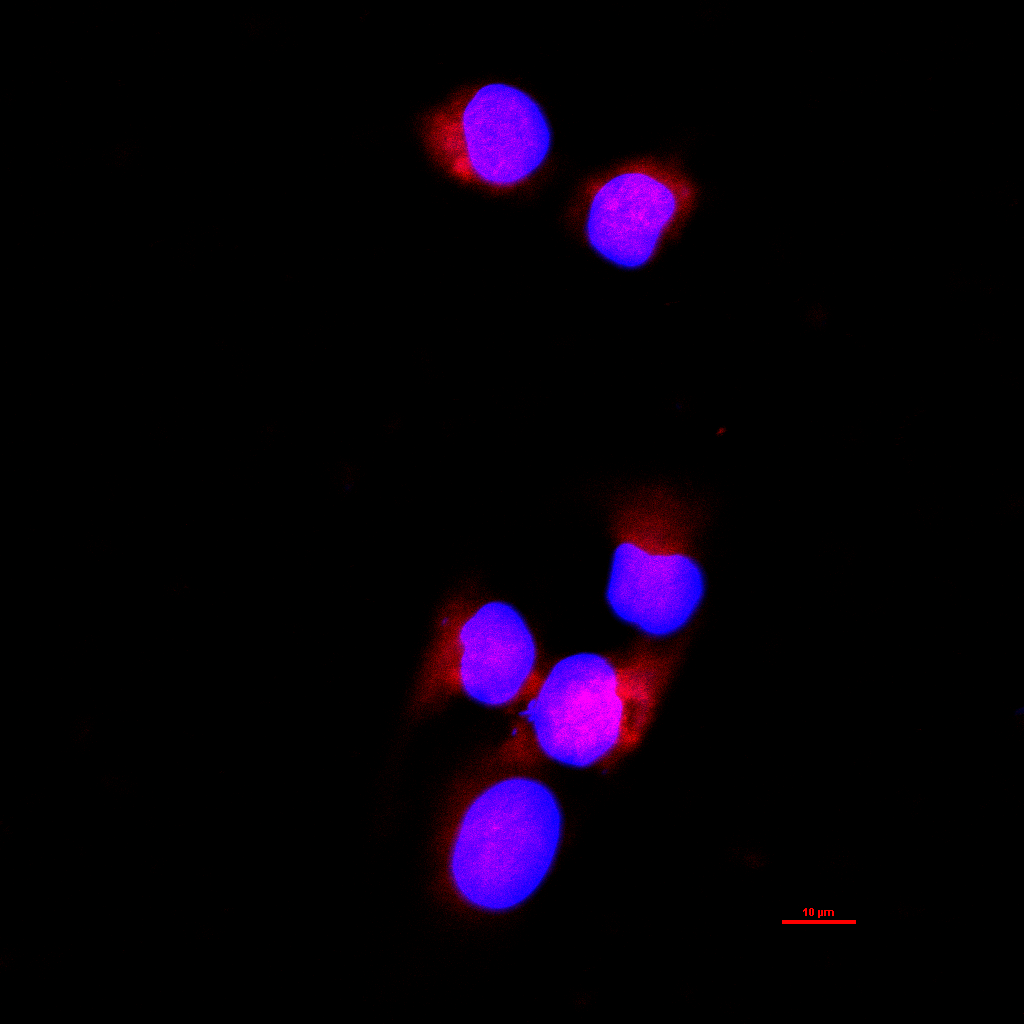

Supplement: Supplementary file 1 [file jox-15-00137-s001.zip › File S1/Figure 2 D) 20组 Merge.tif]

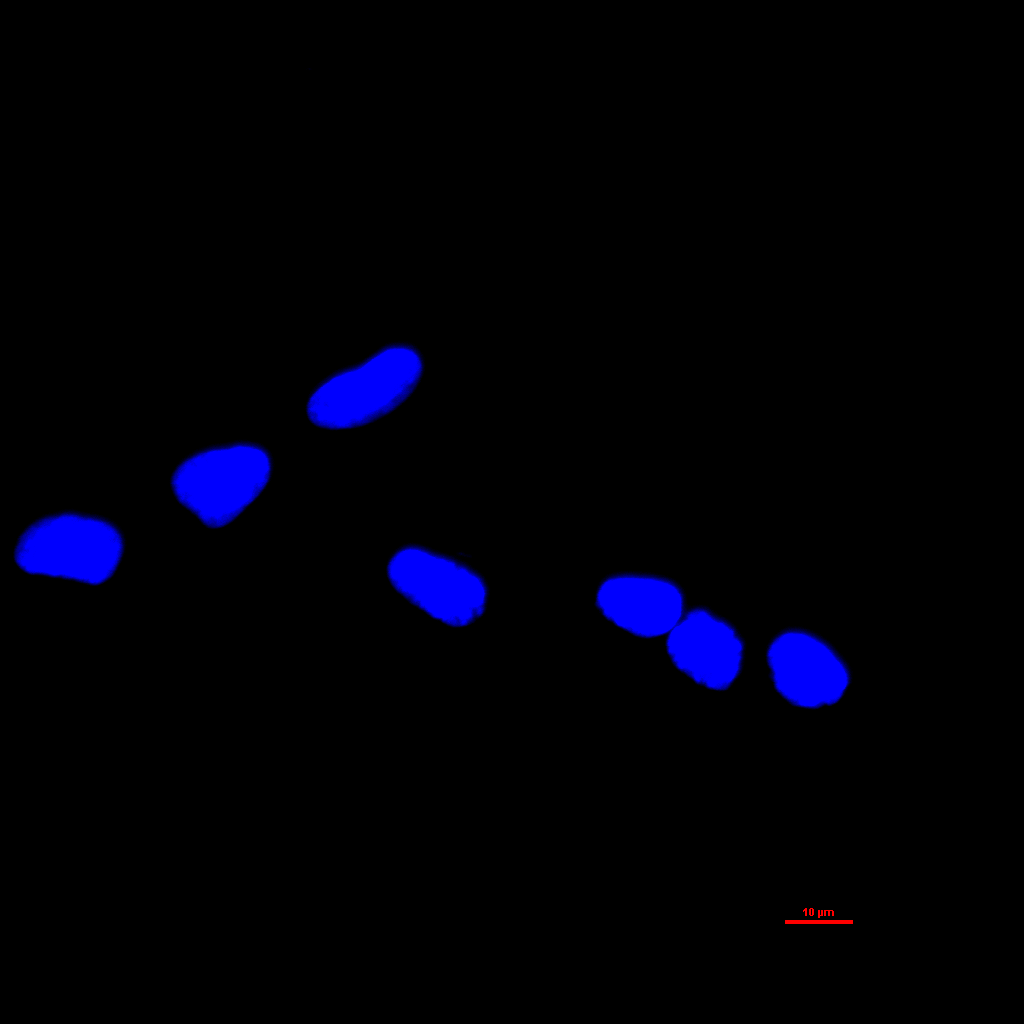

Supplement: Supplementary file 1 [file jox-15-00137-s001.zip › File S1/Figure 2 D) 5组 DAPI.tif]

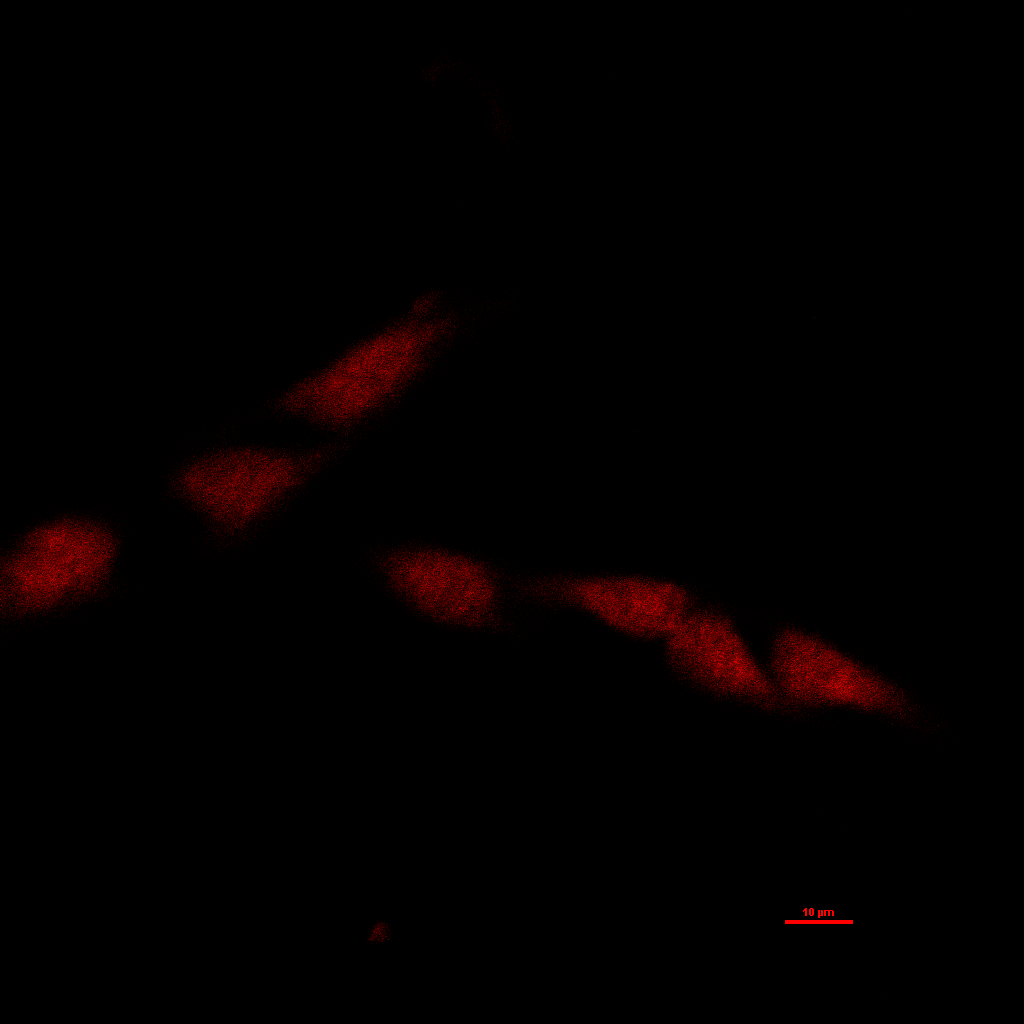

Supplement: Supplementary file 1 [file jox-15-00137-s001.zip › File S1/Figure 2 D) 5组 HSF1.tif]

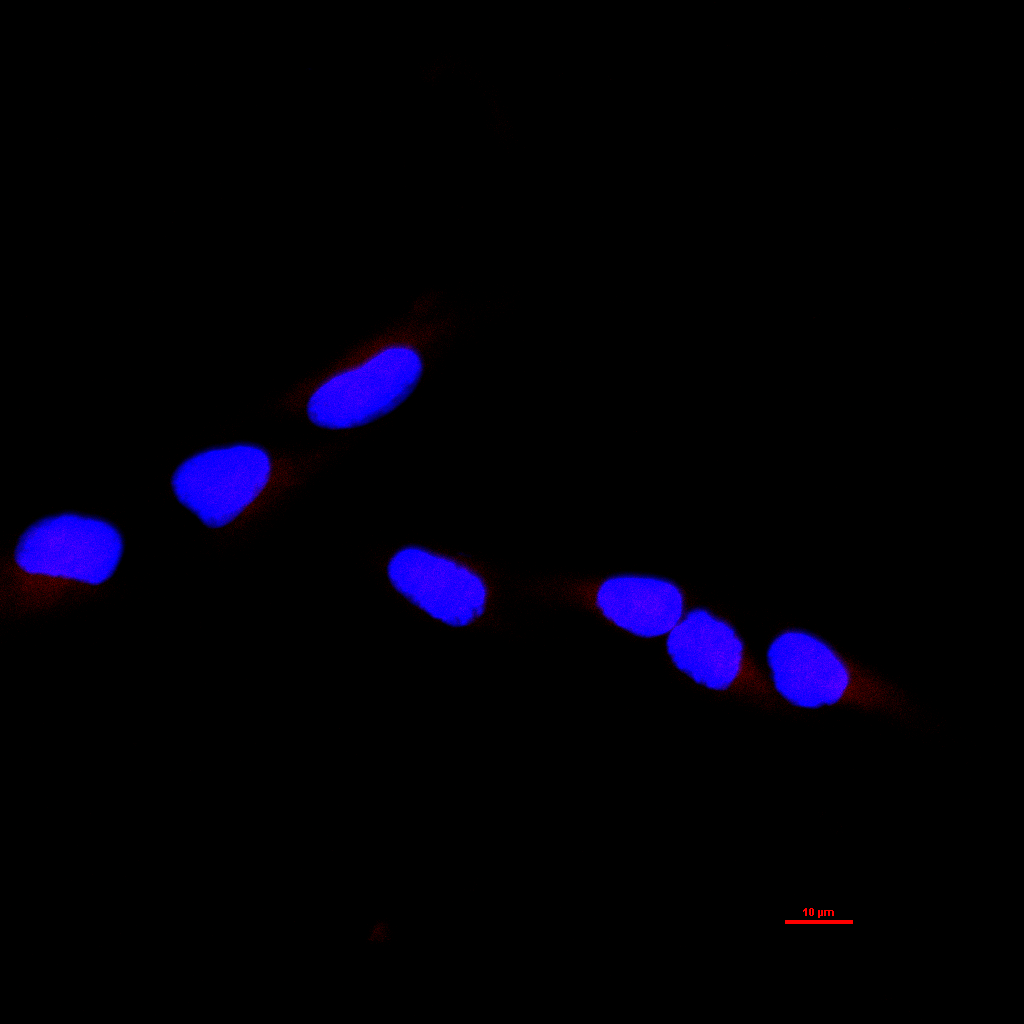

Supplement: Supplementary file 1 [file jox-15-00137-s001.zip › File S1/Figure 2 D) 5组 Merge.tif]

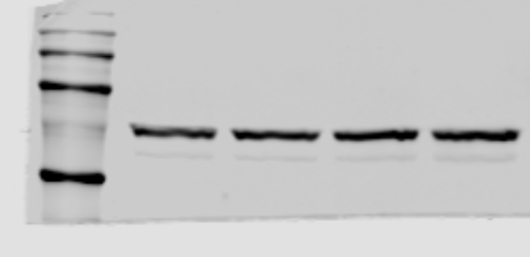

Supplement: Supplementary file 1 [file jox-15-00137-s001.zip › File S1/Figure 2. B)BIP.tif]

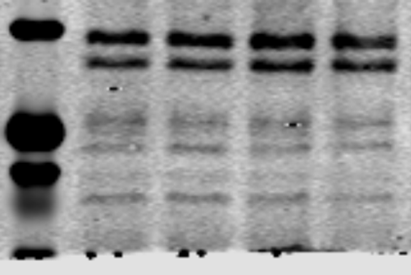

Supplement: Supplementary file 1 [file jox-15-00137-s001.zip › File S1/Figure 2. B)CHOP.tif]

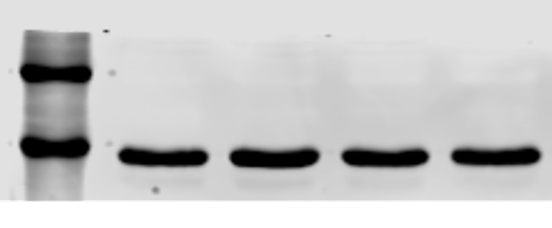

Supplement: Supplementary file 1 [file jox-15-00137-s001.zip › File S1/Figure 2. B)GAPDH.tif]

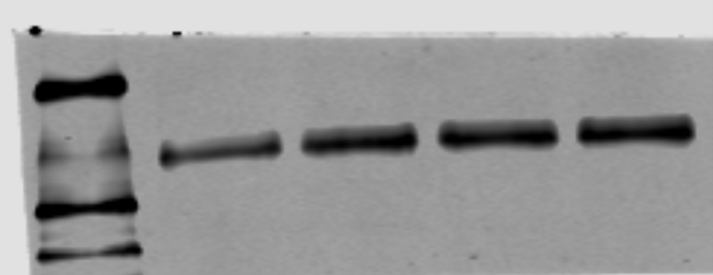

Supplement: Supplementary file 1 [file jox-15-00137-s001.zip › File S1/Figure 2. B)HSF1.tif]

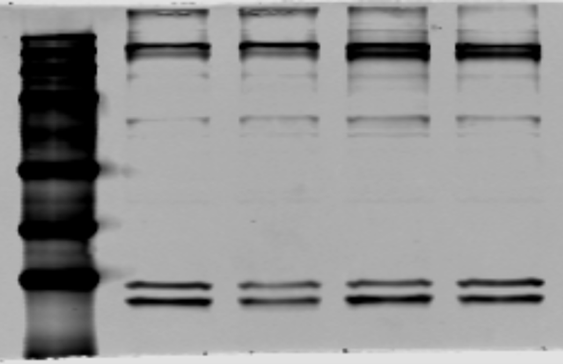

Supplement: Supplementary file 1 [file jox-15-00137-s001.zip › File S1/Figure 2. B)SIRT1(ser27).tif]

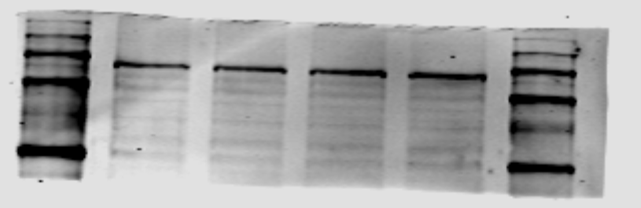

Supplement: Supplementary file 1 [file jox-15-00137-s001.zip › File S1/Figure 2. B)SIRT1(ser47).tif]

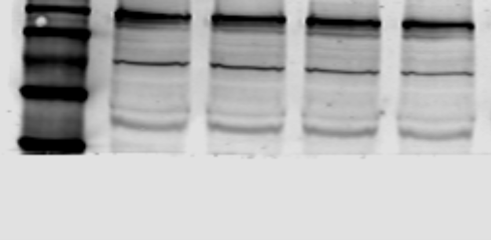

Supplement: Supplementary file 1 [file jox-15-00137-s001.zip › File S1/Figure 2. B)SIRT1.tif]

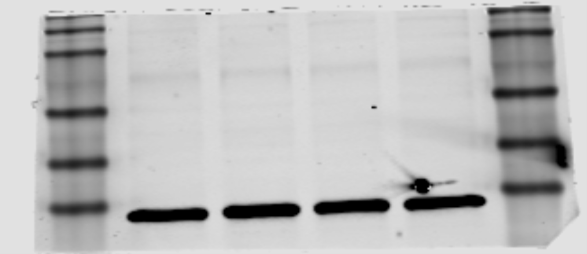

Supplement: Supplementary file 1 [file jox-15-00137-s001.zip › File S1/Figure 2. C)Input GAPDH.tif]

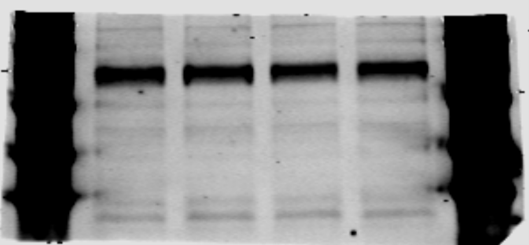

Supplement: Supplementary file 1 [file jox-15-00137-s001.zip › File S1/Figure 2. C)Input HSF1.tif]

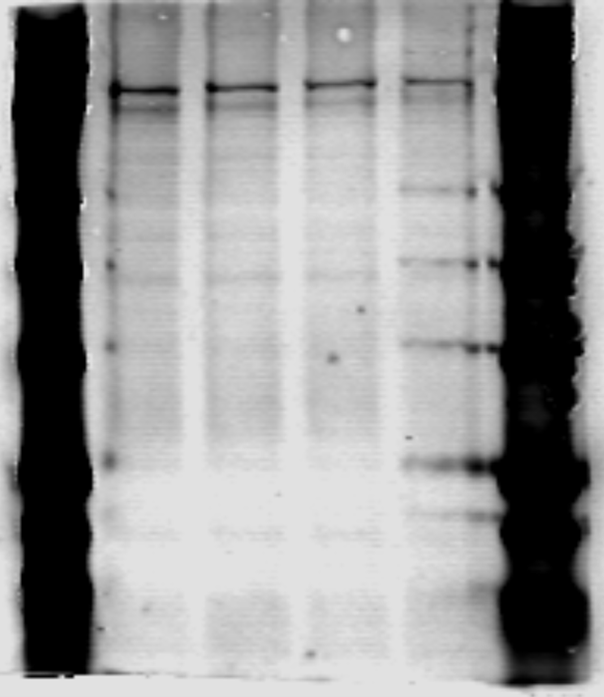

Supplement: Supplementary file 1 [file jox-15-00137-s001.zip › File S1/Figure 2. C)IP Acetylated lysine.tif]

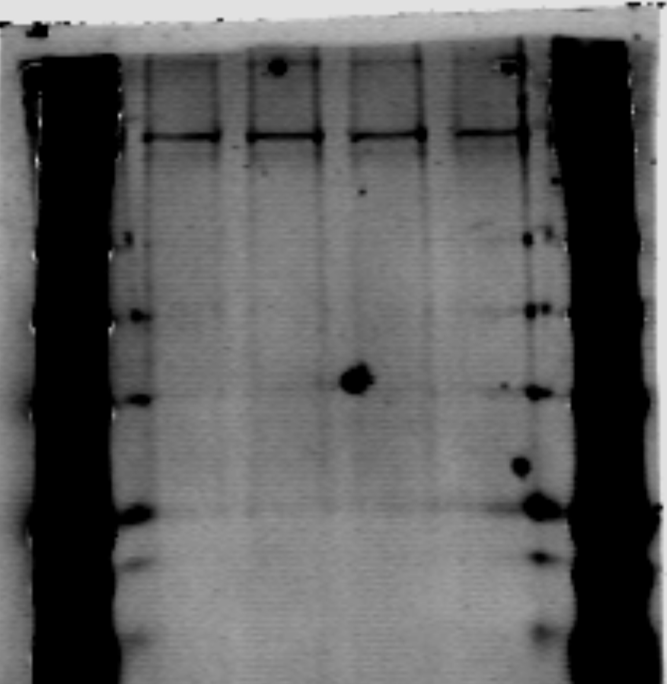

Supplement: Supplementary file 1 [file jox-15-00137-s001.zip › File S1/Figure 2. C)IP HSF1.tif]

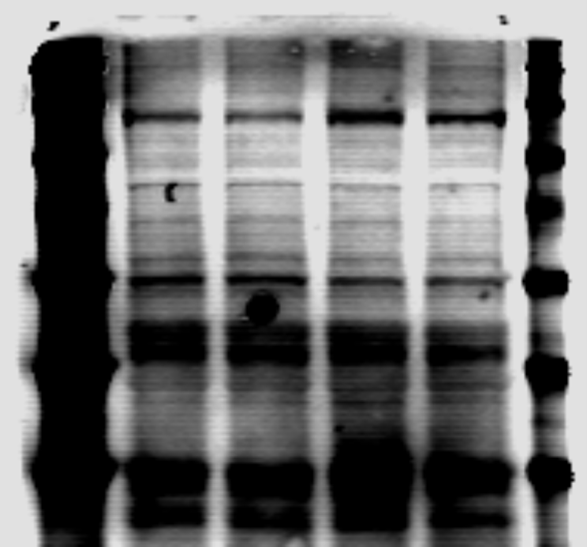

Supplement: Supplementary file 1 [file jox-15-00137-s001.zip › File S1/Figure 3 A) SIRT1 (ser47).tif]

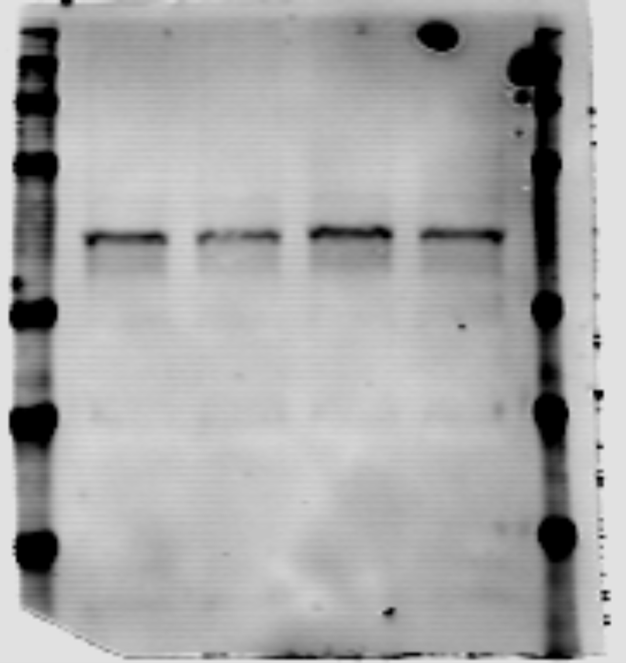

Supplement: Supplementary file 1 [file jox-15-00137-s001.zip › File S1/Figure 3. A) BIP.tif]

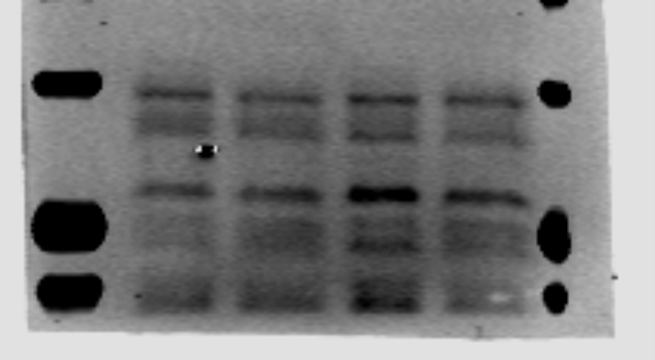

Supplement: Supplementary file 1 [file jox-15-00137-s001.zip › File S1/Figure 3. A) CHOP.tif]

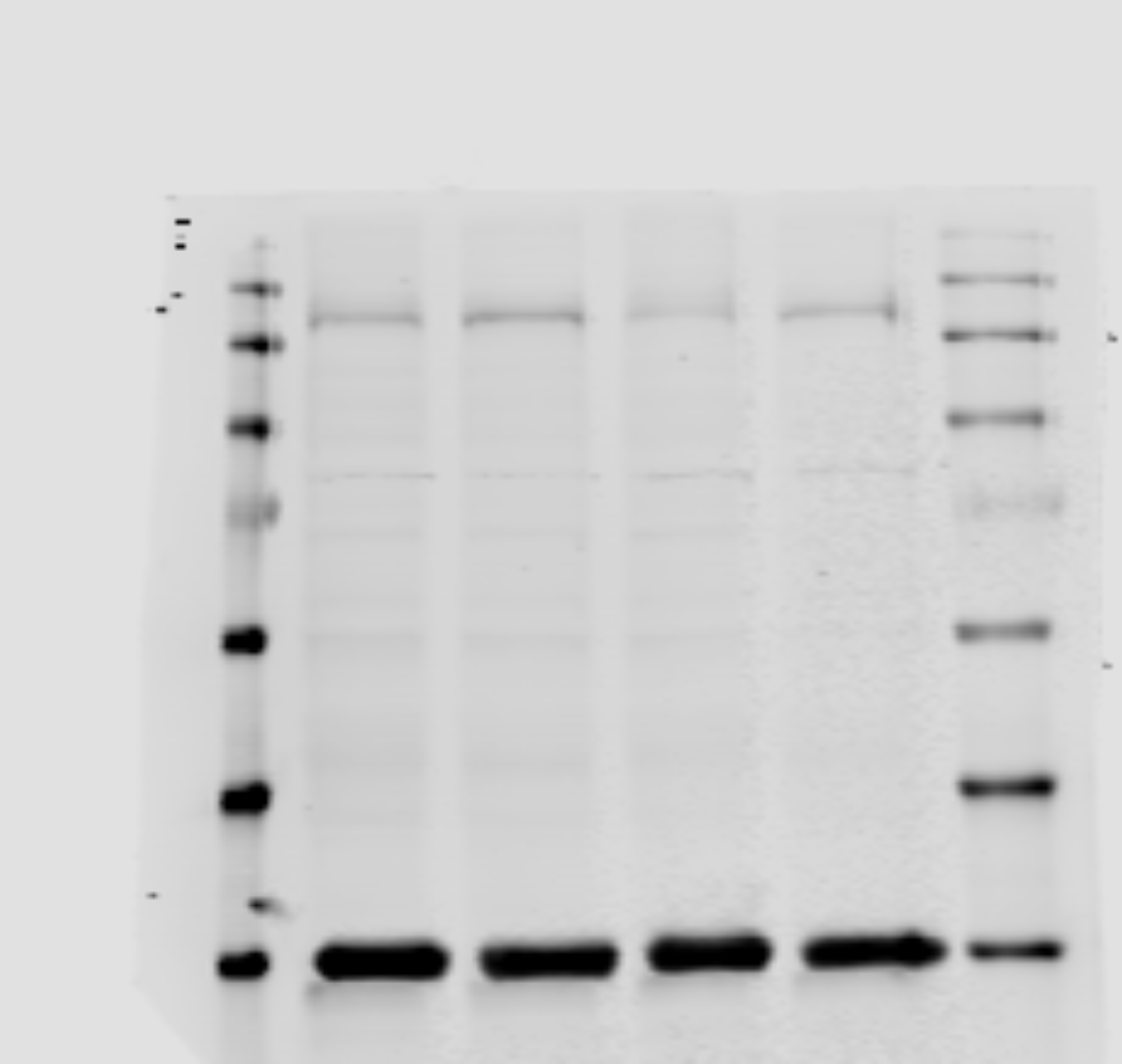

Supplement: Supplementary file 1 [file jox-15-00137-s001.zip › File S1/Figure 3. A) GAPDH.tif]

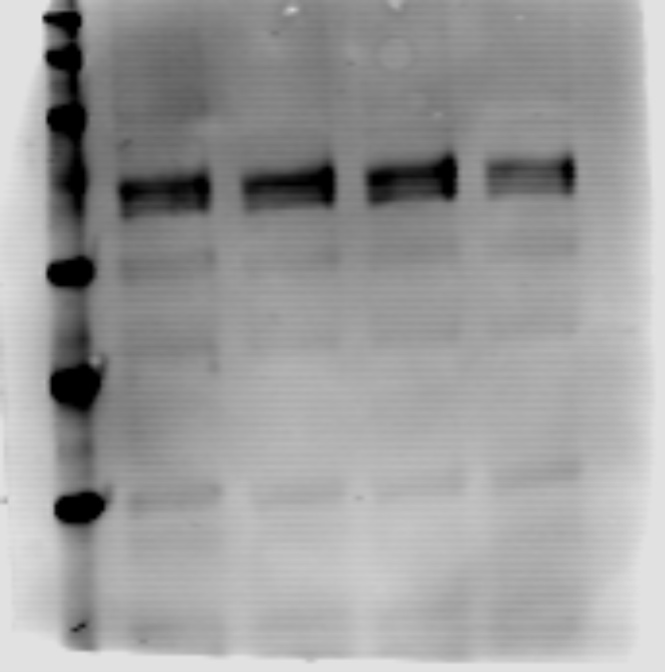

Supplement: Supplementary file 1 [file jox-15-00137-s001.zip › File S1/Figure 3. A) HSF1.tif]

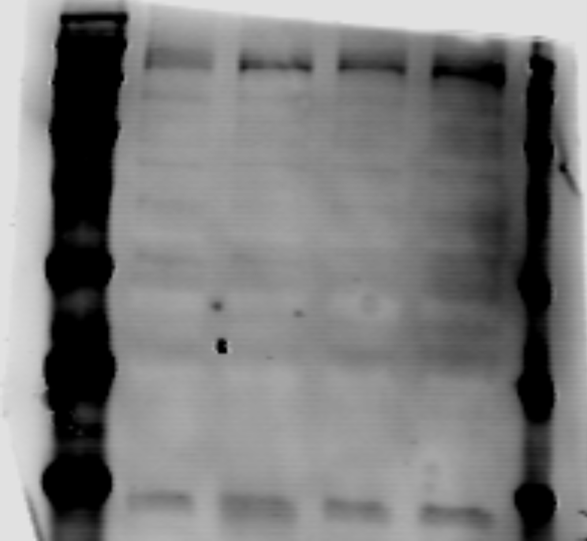

Supplement: Supplementary file 1 [file jox-15-00137-s001.zip › File S1/Figure 3. A) SIRT1 (ser27).tif]

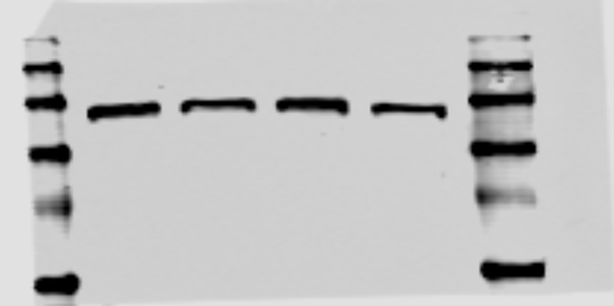

Supplement: Supplementary file 1 [file jox-15-00137-s001.zip › File S1/Figure 3. A) SIRT1.tif]

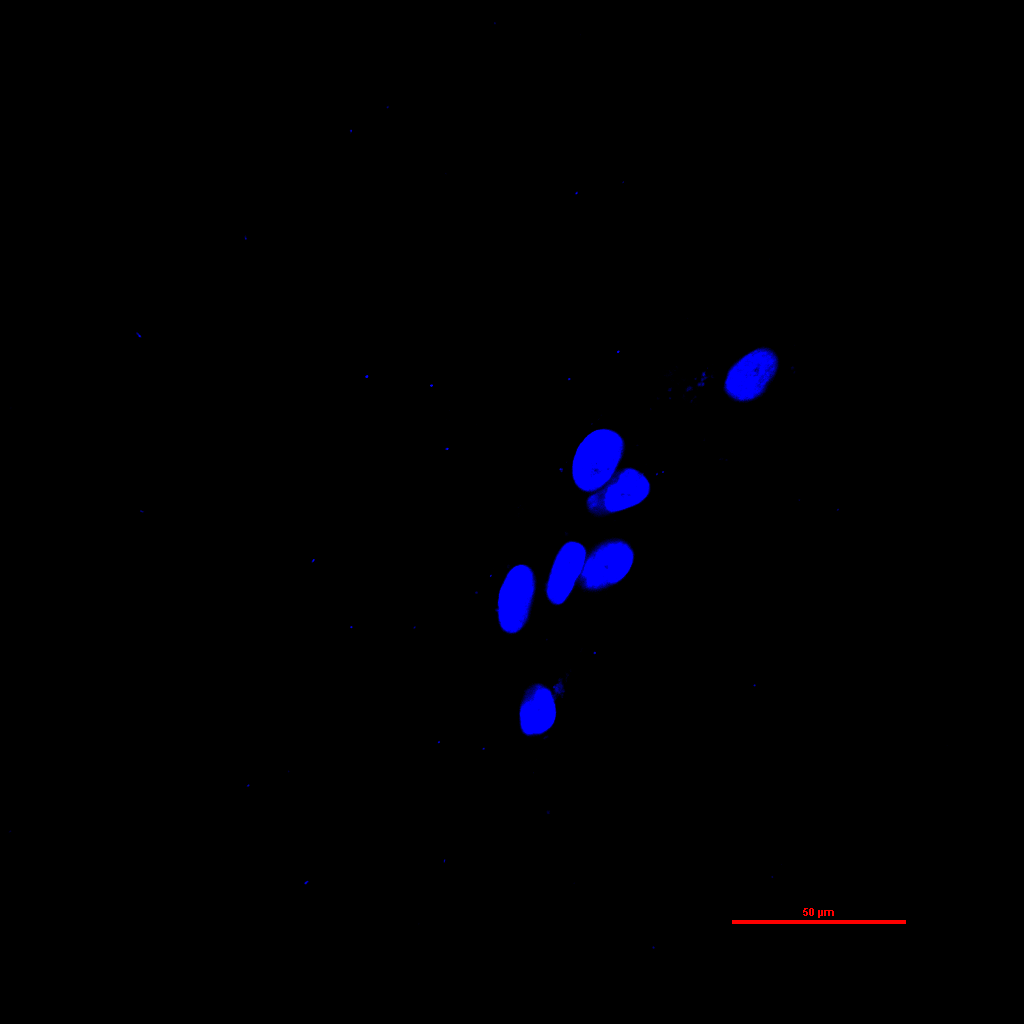

Supplement: Supplementary file 1 [file jox-15-00137-s001.zip › File S1/Figure 3. B) 4-PBA+ SiNPs- DAPI.tif]

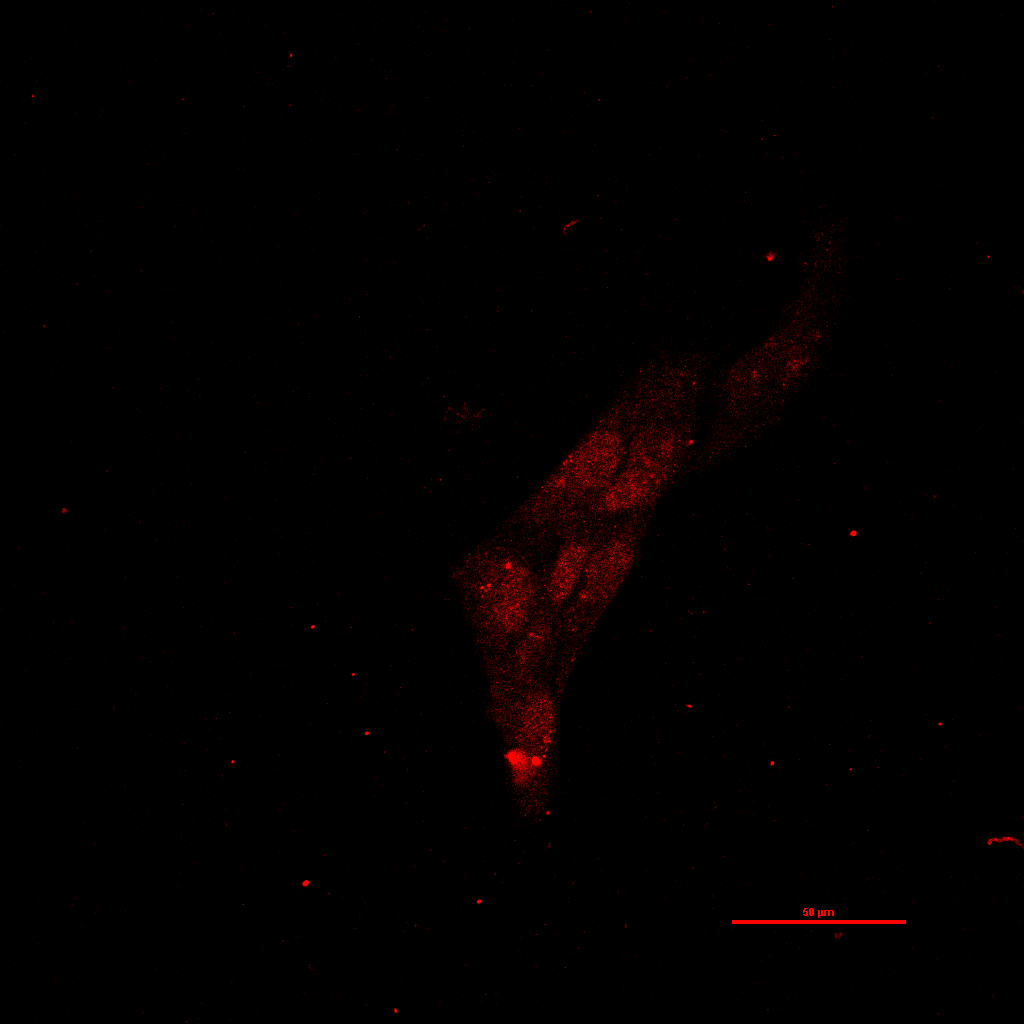

Supplement: Supplementary file 1 [file jox-15-00137-s001.zip › File S1/Figure 3. B) 4-PBA+ SiNPs- HSF1.tif]

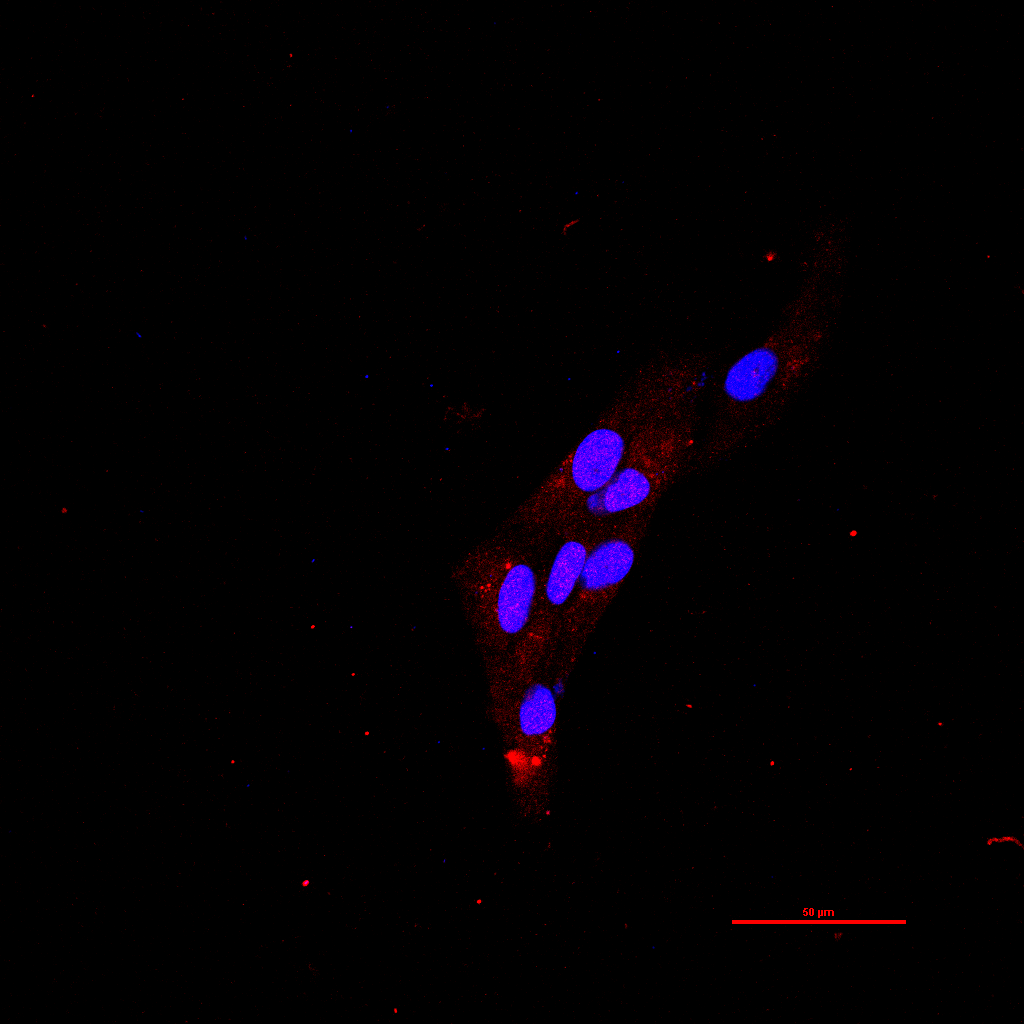

Supplement: Supplementary file 1 [file jox-15-00137-s001.zip › File S1/Figure 3. B) 4-PBA+ SiNPs- Merge.tif]

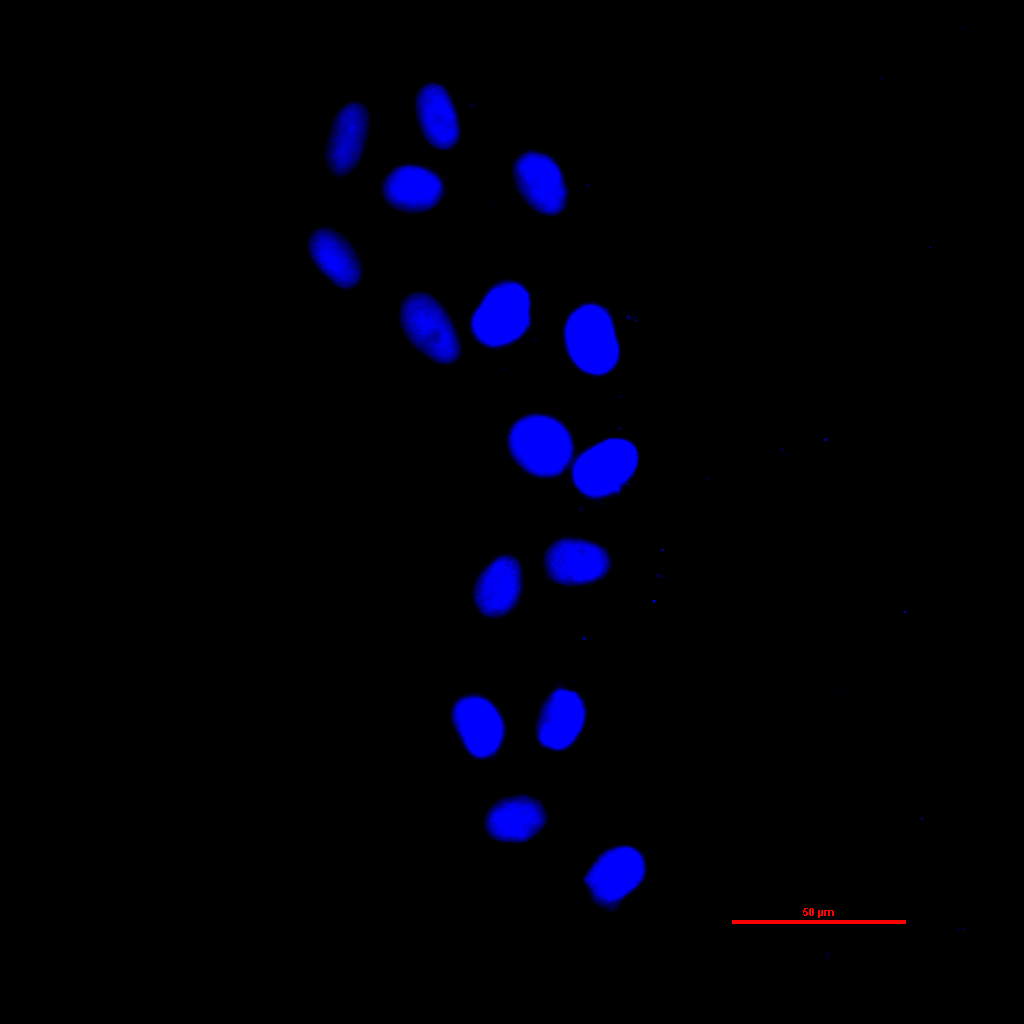

Supplement: Supplementary file 1 [file jox-15-00137-s001.zip › File S1/Figure 3. B) 4-PBA+ SiNPs+ DAPI.tif]

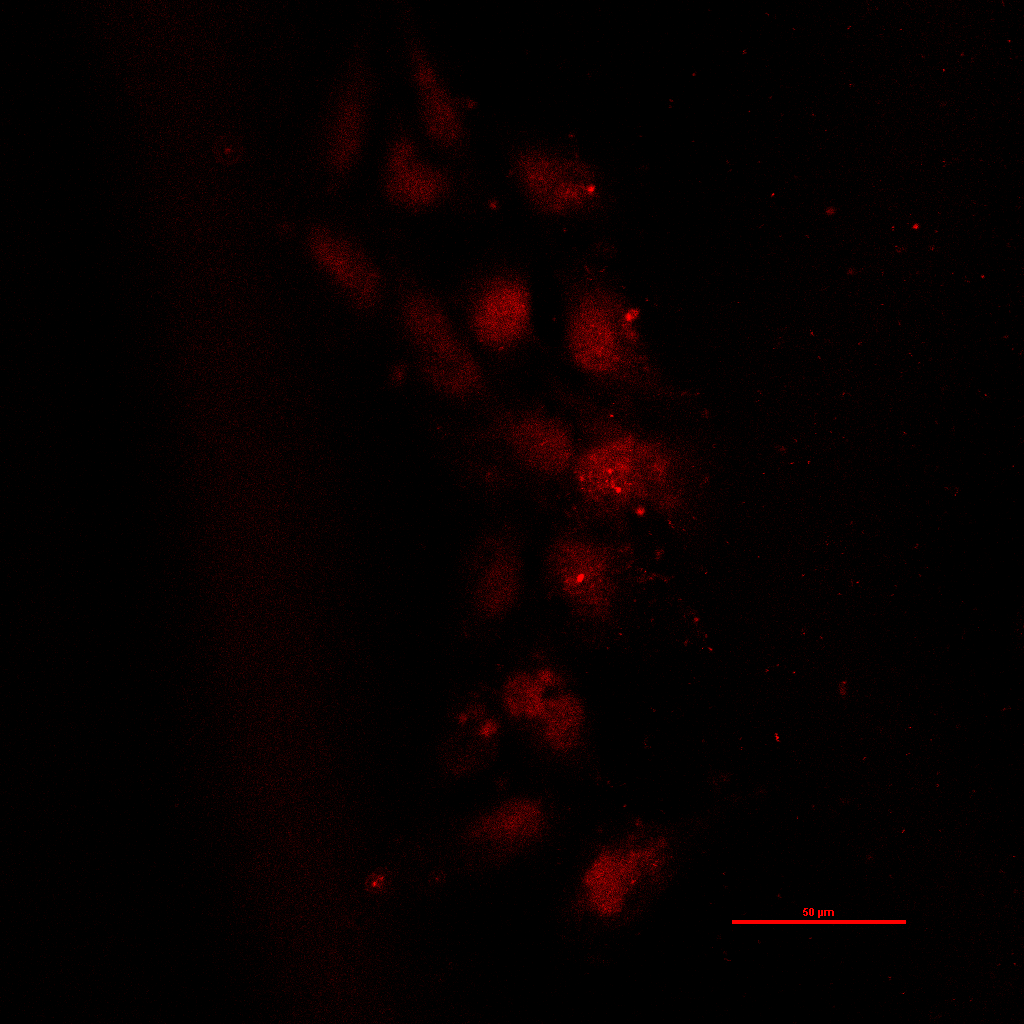

Supplement: Supplementary file 1 [file jox-15-00137-s001.zip › File S1/Figure 3. B) 4-PBA+ SiNPs+ HSF1.tif]

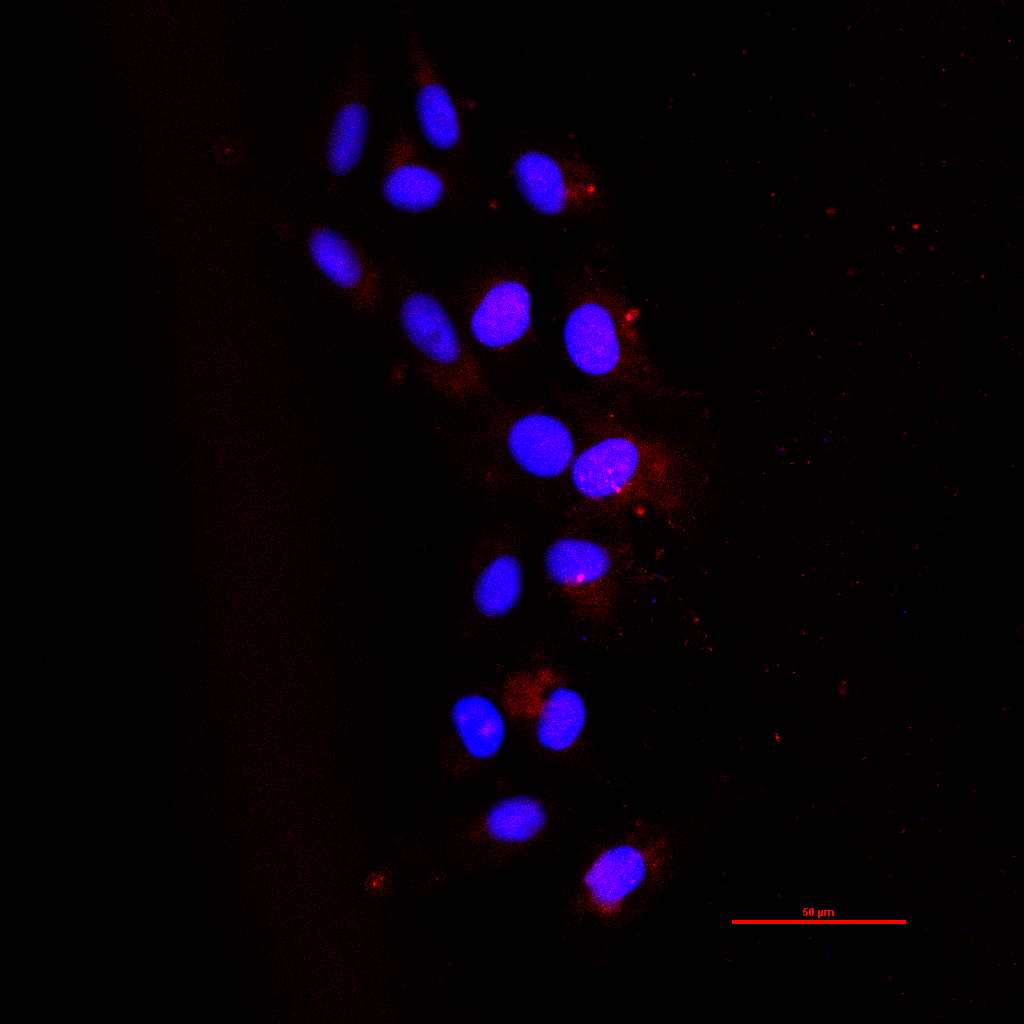

Supplement: Supplementary file 1 [file jox-15-00137-s001.zip › File S1/Figure 3. B) 4-PBA+ SiNPs+ Merge.tif]

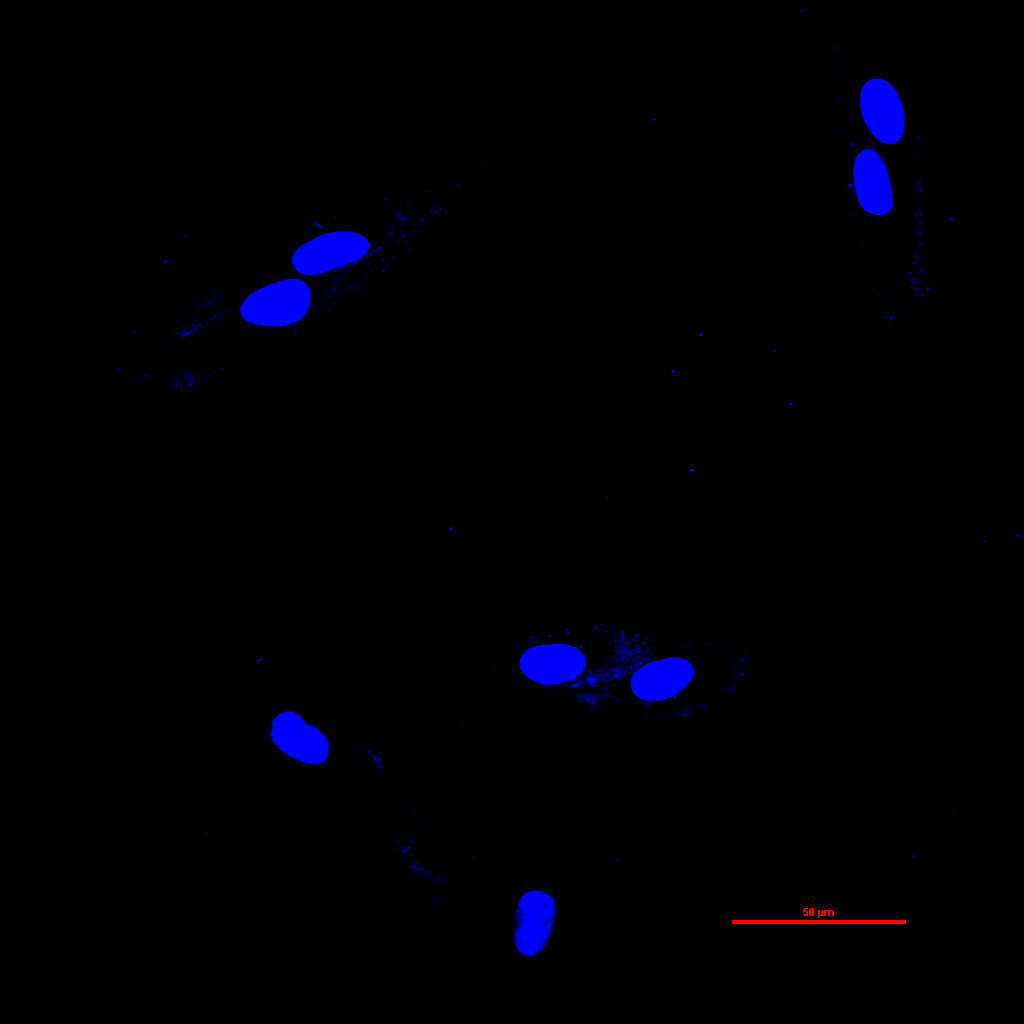

Supplement: Supplementary file 1 [file jox-15-00137-s001.zip › File S1/Figure 3. B) 4-PBA- SiNPs- DAPI.tif]

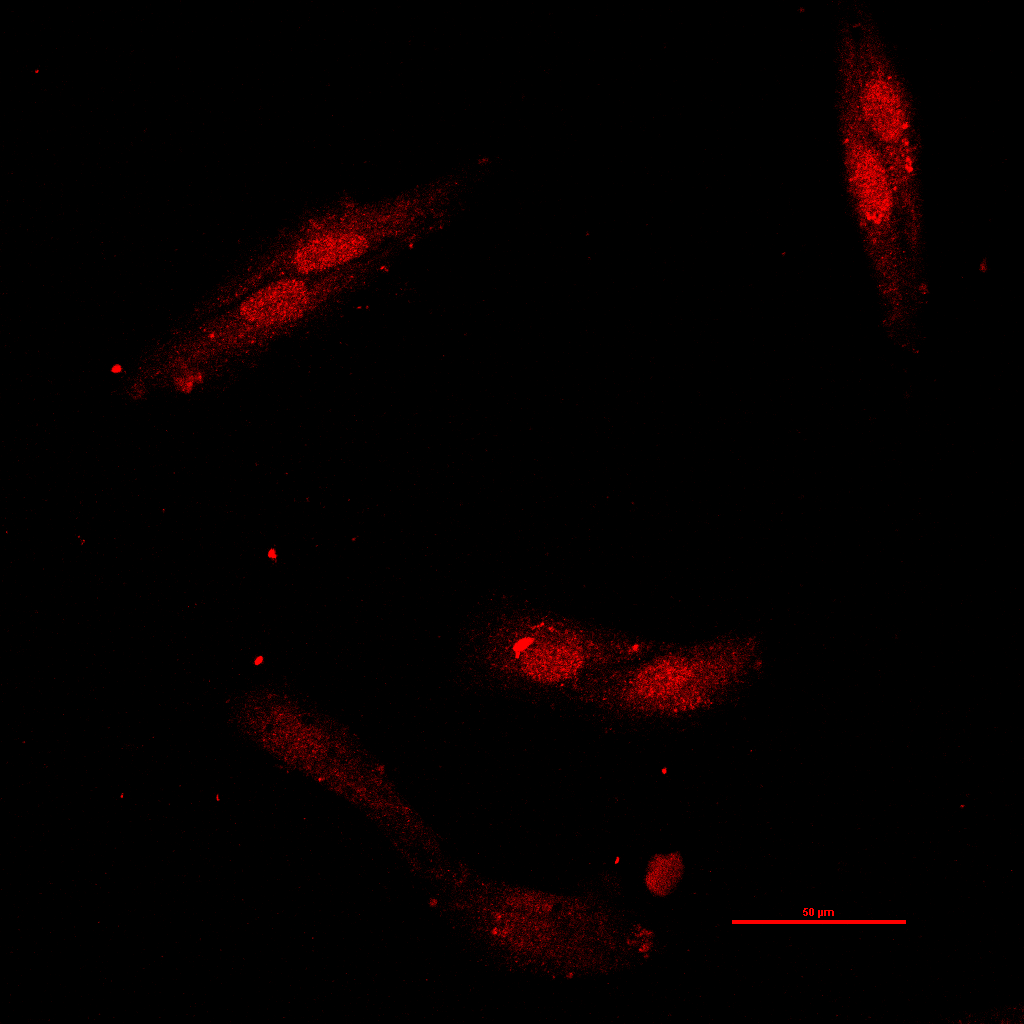

Supplement: Supplementary file 1 [file jox-15-00137-s001.zip › File S1/Figure 3. B) 4-PBA- SiNPs- HSF1.tif]

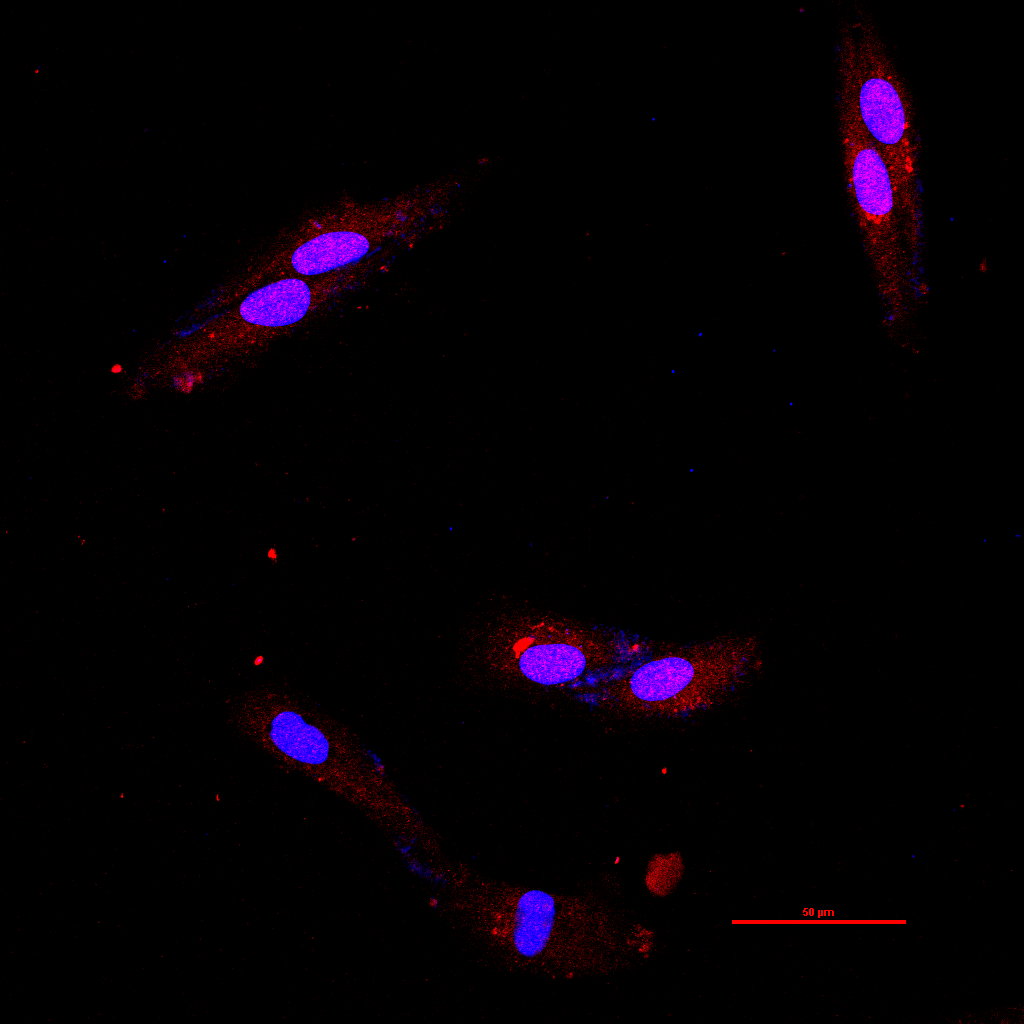

Supplement: Supplementary file 1 [file jox-15-00137-s001.zip › File S1/Figure 3. B) 4-PBA- SiNPs- Merge.tif]

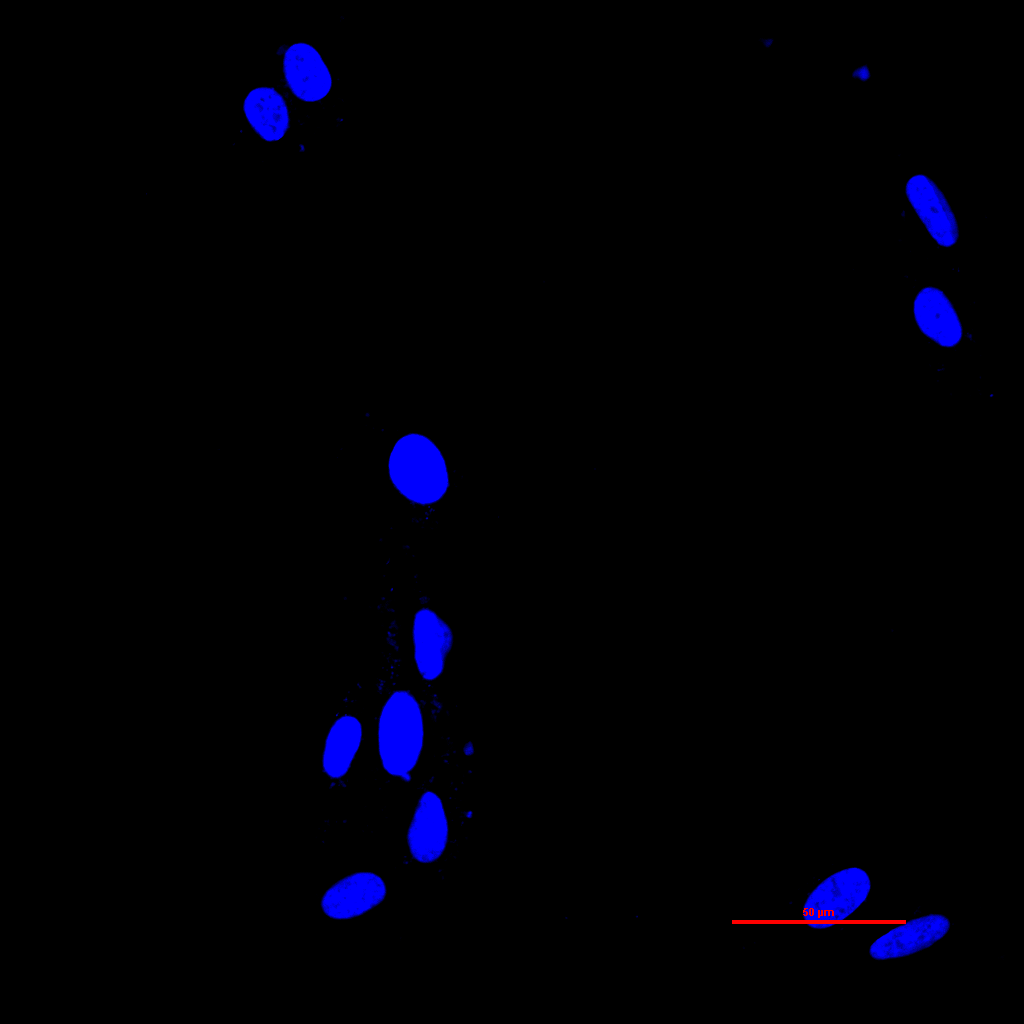

Supplement: Supplementary file 1 [file jox-15-00137-s001.zip › File S1/Figure 3. B) 4-PBA- SiNPs+ DAPI.tif]

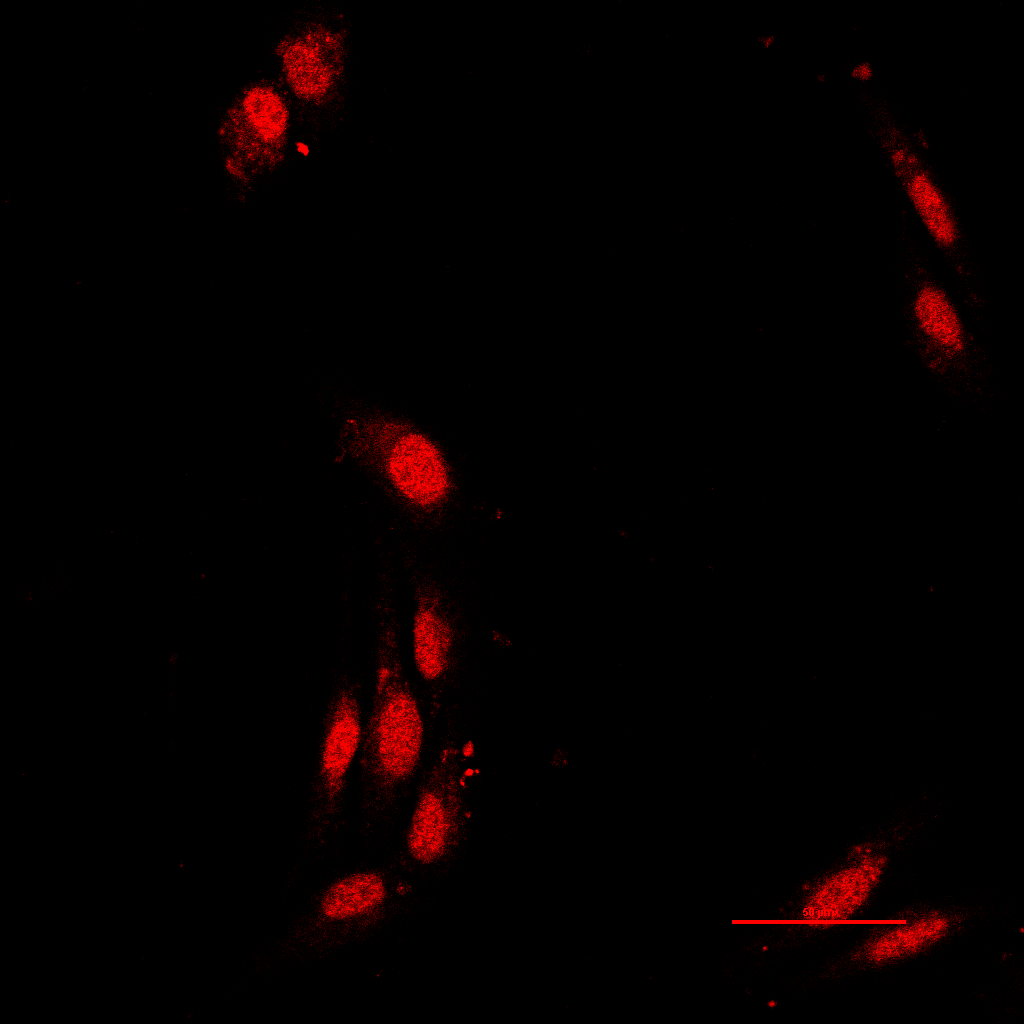

Supplement: Supplementary file 1 [file jox-15-00137-s001.zip › File S1/Figure 3. B) 4-PBA- SiNPs+ HSF1.tif]

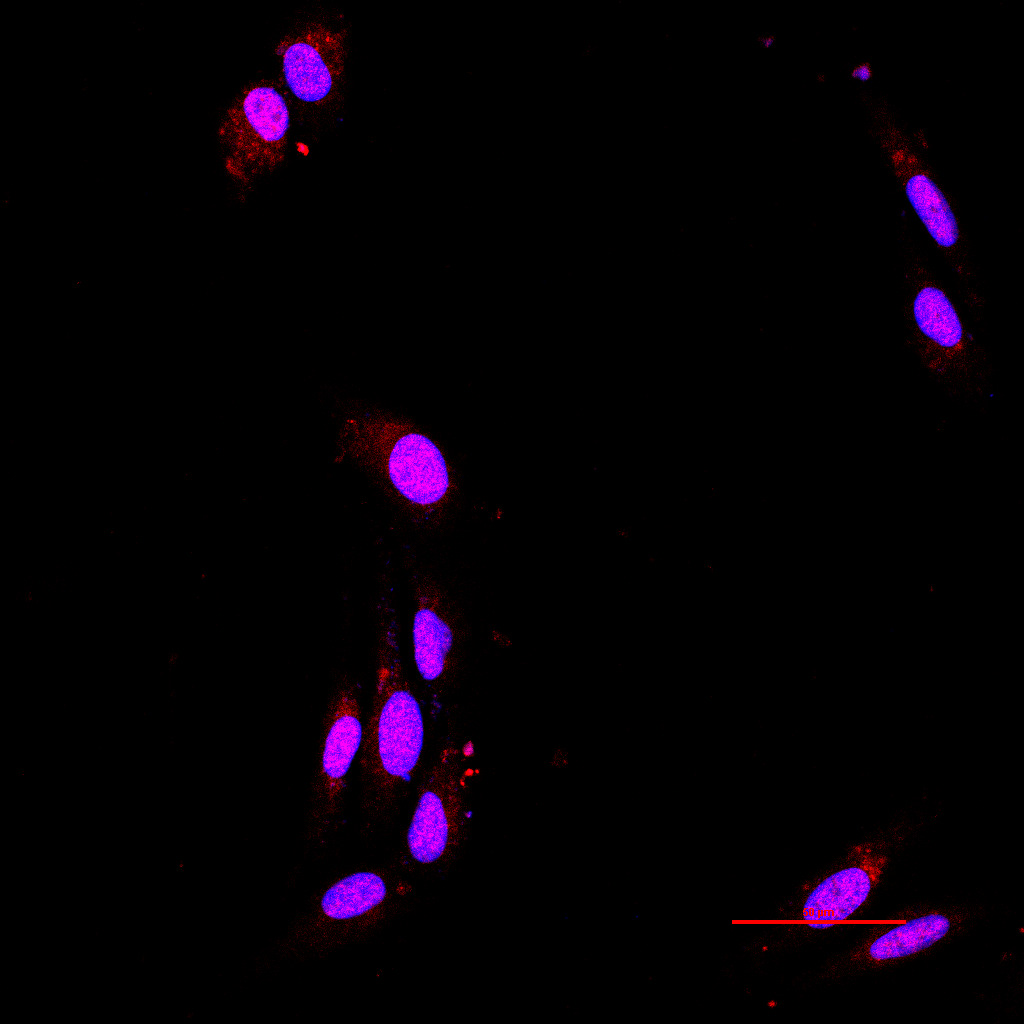

Supplement: Supplementary file 1 [file jox-15-00137-s001.zip › File S1/Figure 3. B) 4-PBA- SiNPs+ Merge.tif]

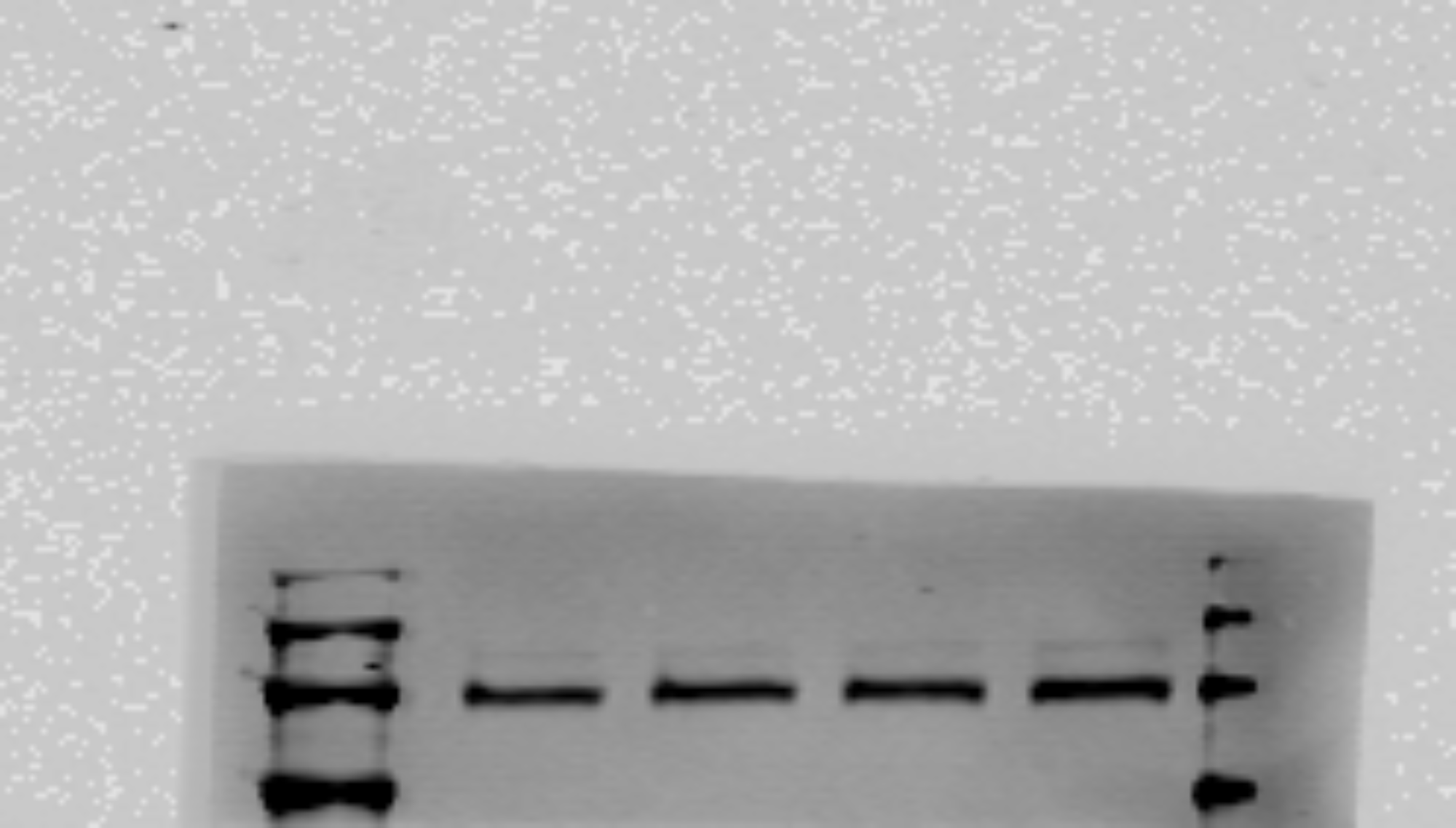

Supplement: Supplementary file 1 [file jox-15-00137-s001.zip › File S1/Figure 4 A) N-cadherin.tif]

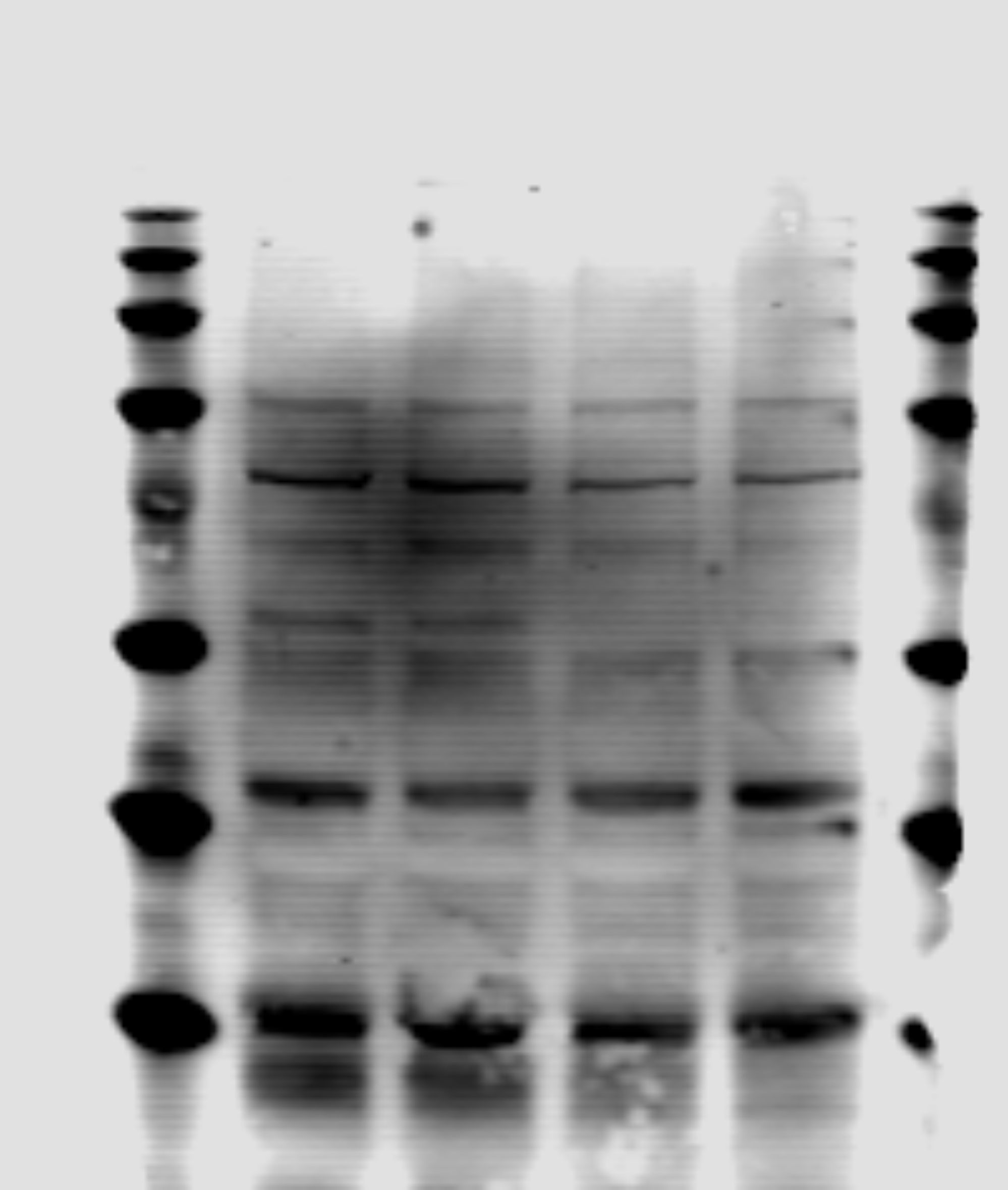

Supplement: Supplementary file 1 [file jox-15-00137-s001.zip › File S1/Figure 4. A) E-cadherin.tif]

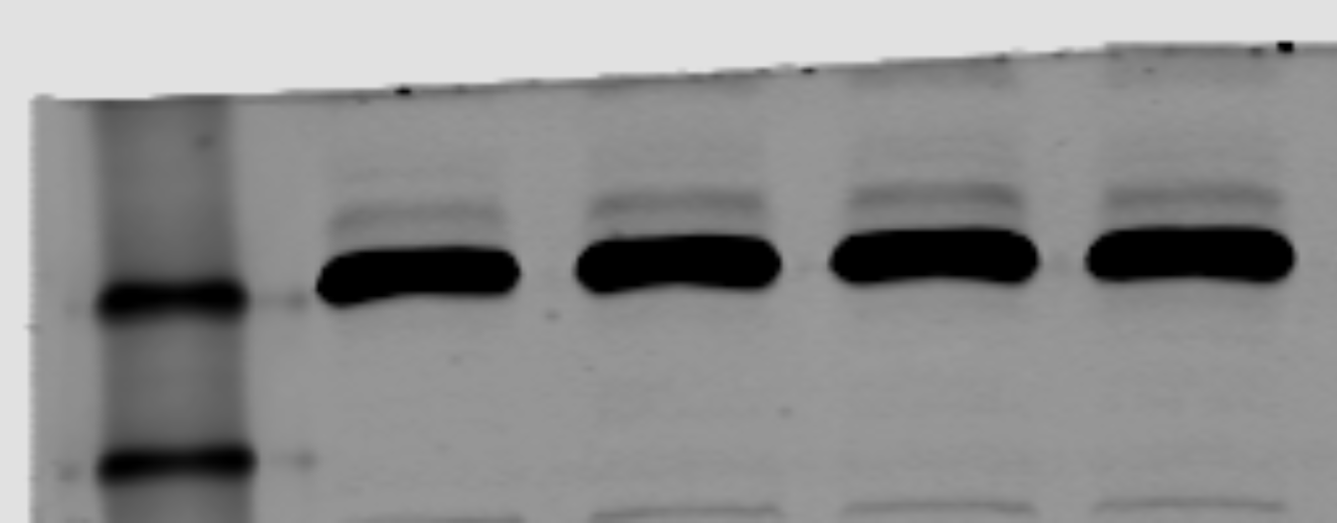

Supplement: Supplementary file 1 [file jox-15-00137-s001.zip › File S1/Figure 4. A) GAPDH.tif]

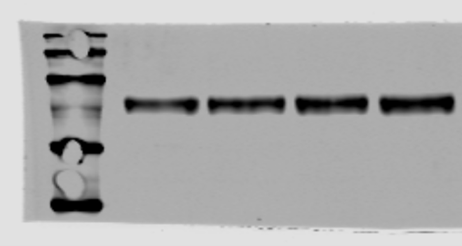

Supplement: Supplementary file 1 [file jox-15-00137-s001.zip › File S1/Figure 4. A)HSF1.tif]

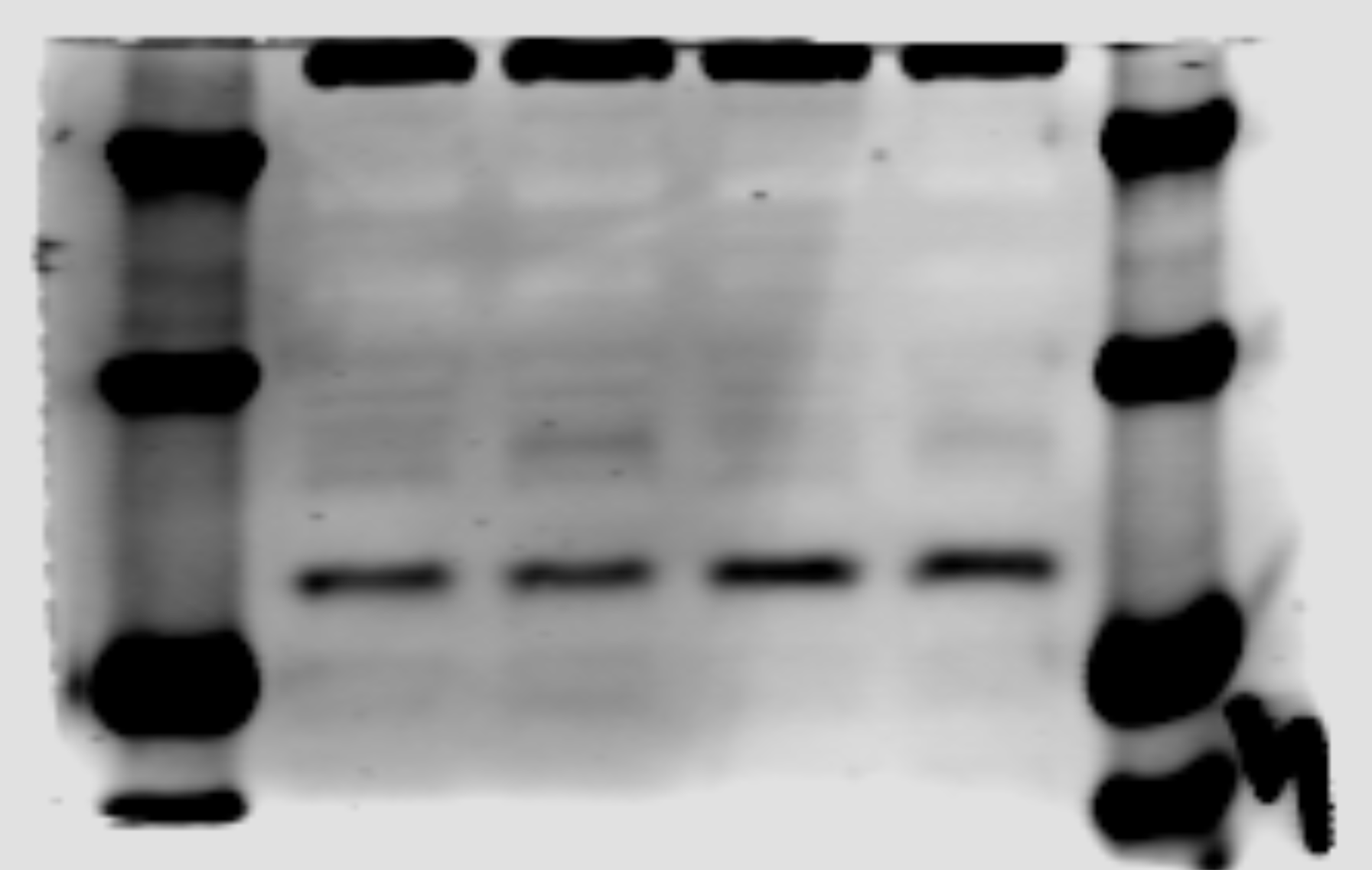

Supplement: Supplementary file 1 [file jox-15-00137-s001.zip › File S1/Figure 4. A)HSP27.tif]

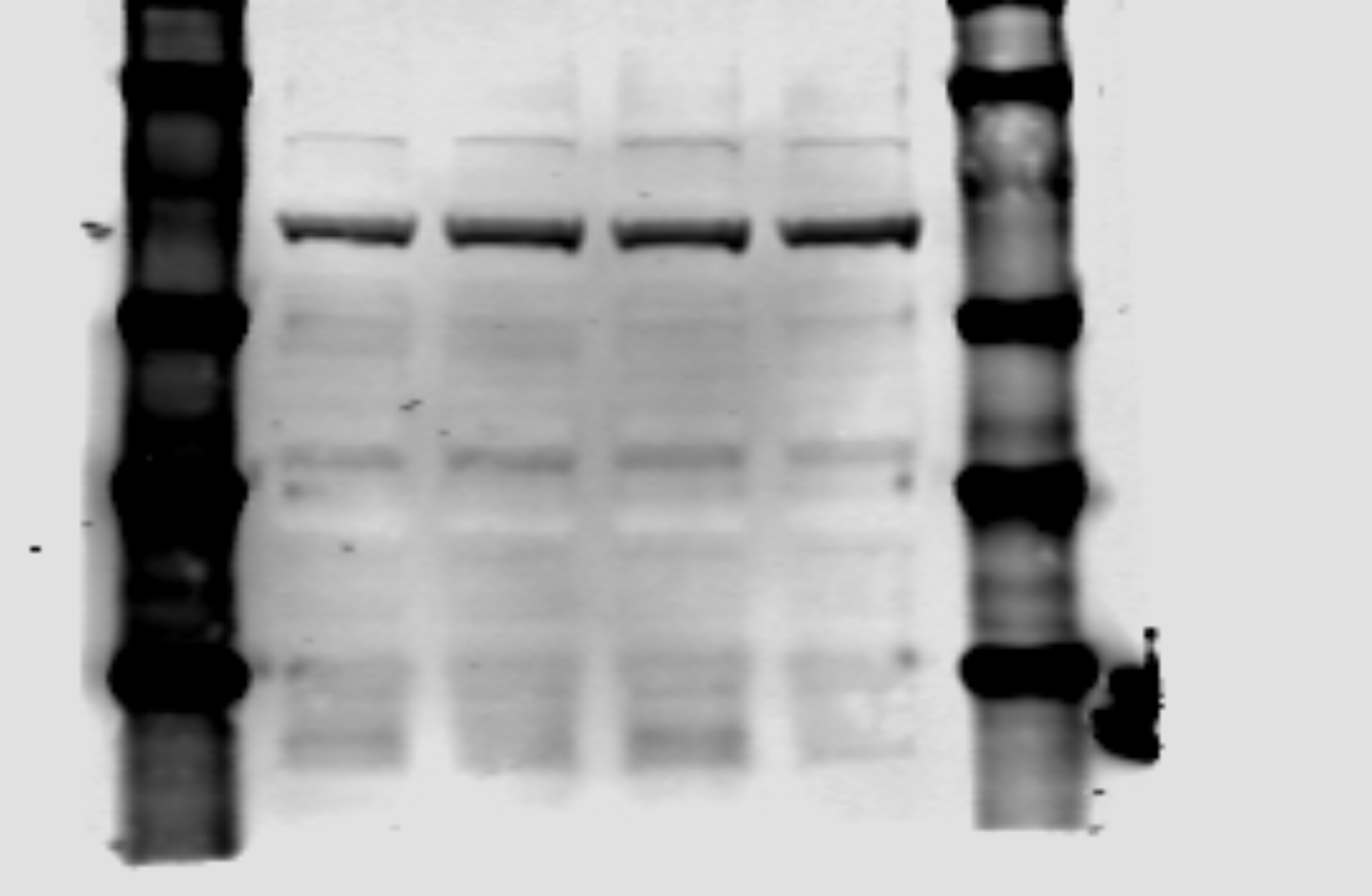

Supplement: Supplementary file 1 [file jox-15-00137-s001.zip › File S1/Figure 4. A)hsp70.tif]

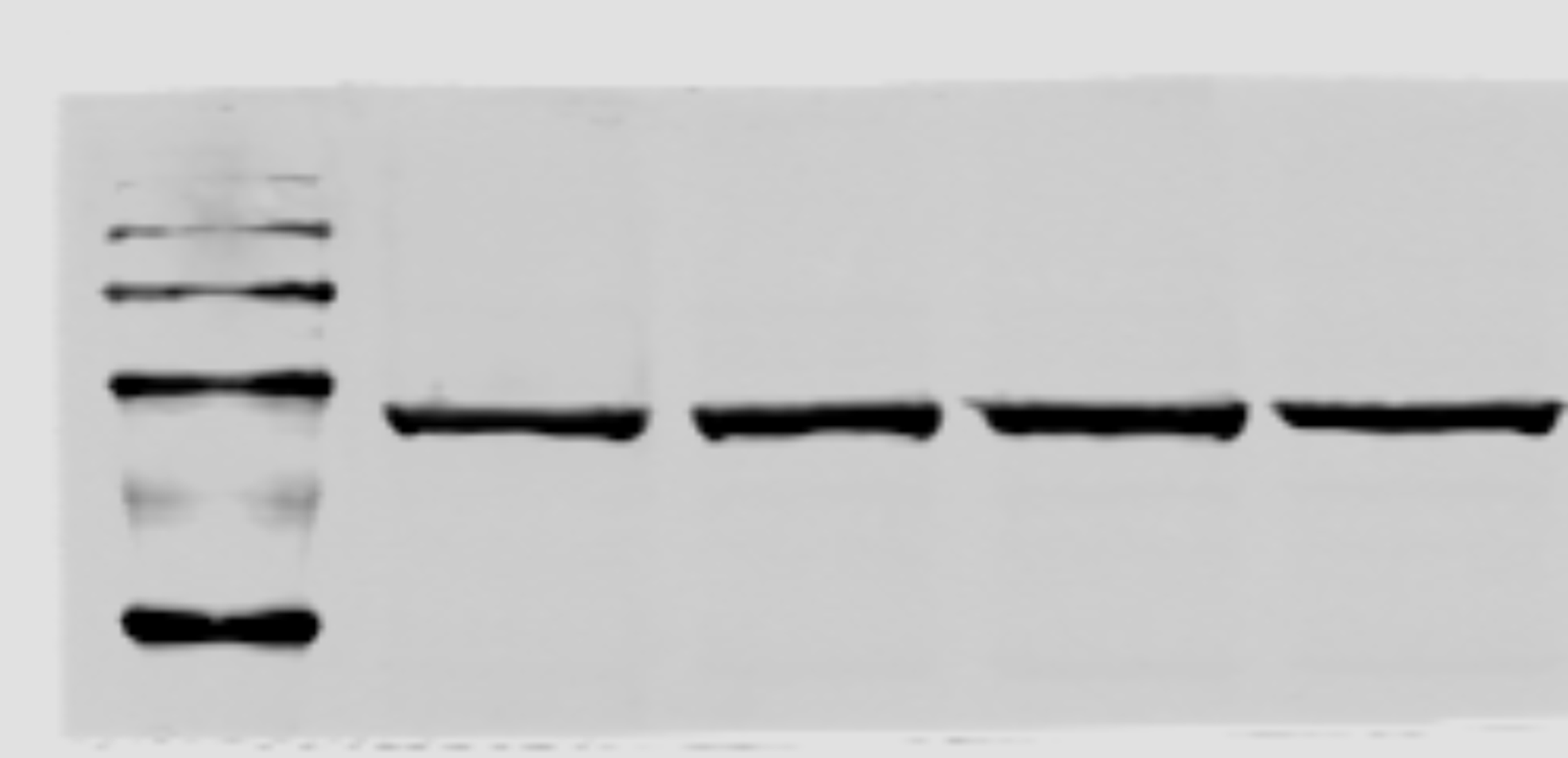

Supplement: Supplementary file 1 [file jox-15-00137-s001.zip › File S1/Figure 4. A)HSP90.tif]

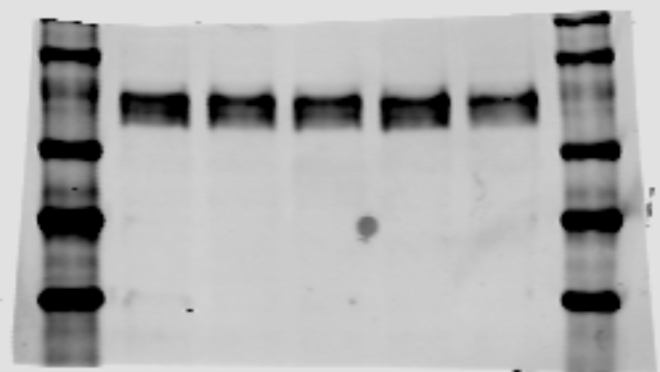

Supplement: Supplementary file 1 [file jox-15-00137-s001.zip › File S1/Figure 5. A) HSF1.tif]

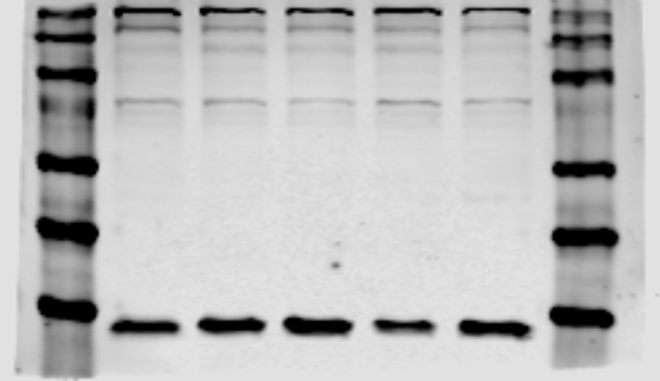

Supplement: Supplementary file 1 [file jox-15-00137-s001.zip › File S1/Figure 5. A) P-SIRT1(ser27).tif]

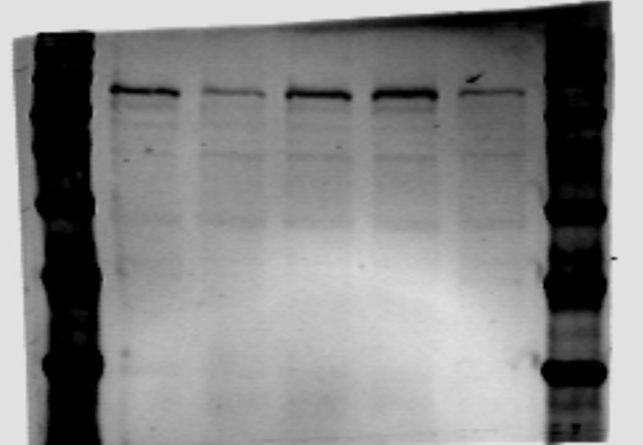

Supplement: Supplementary file 1 [file jox-15-00137-s001.zip › File S1/Figure 5. A) P-SIRT1(ser47).tif]

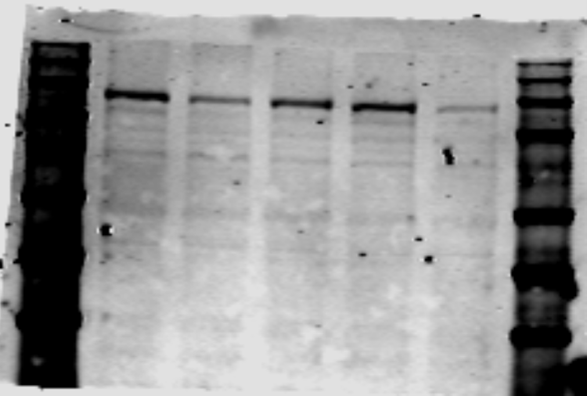

Supplement: Supplementary file 1 [file jox-15-00137-s001.zip › File S1/Figure 5. A) SIRT1.tif]

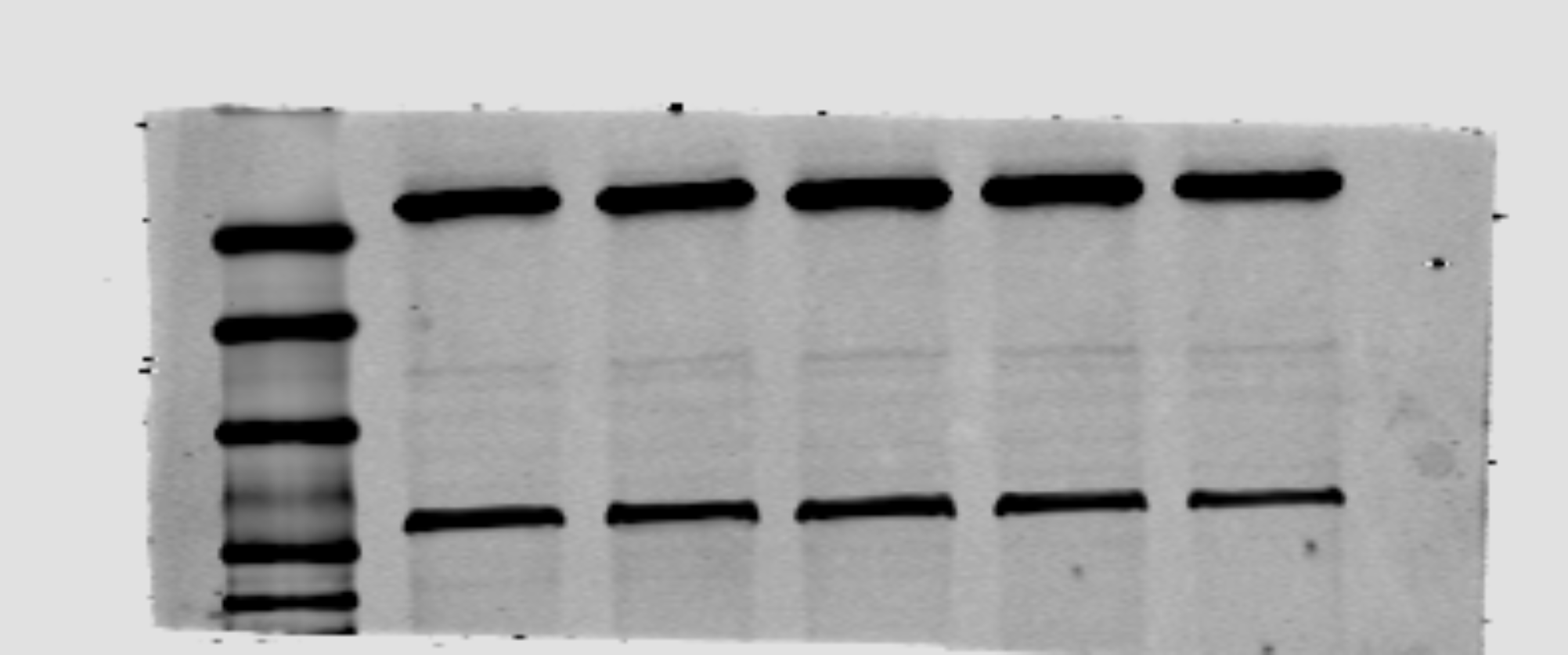

Supplement: Supplementary file 1 [file jox-15-00137-s001.zip › File S1/Figure 5. A)GAPDH.tif]

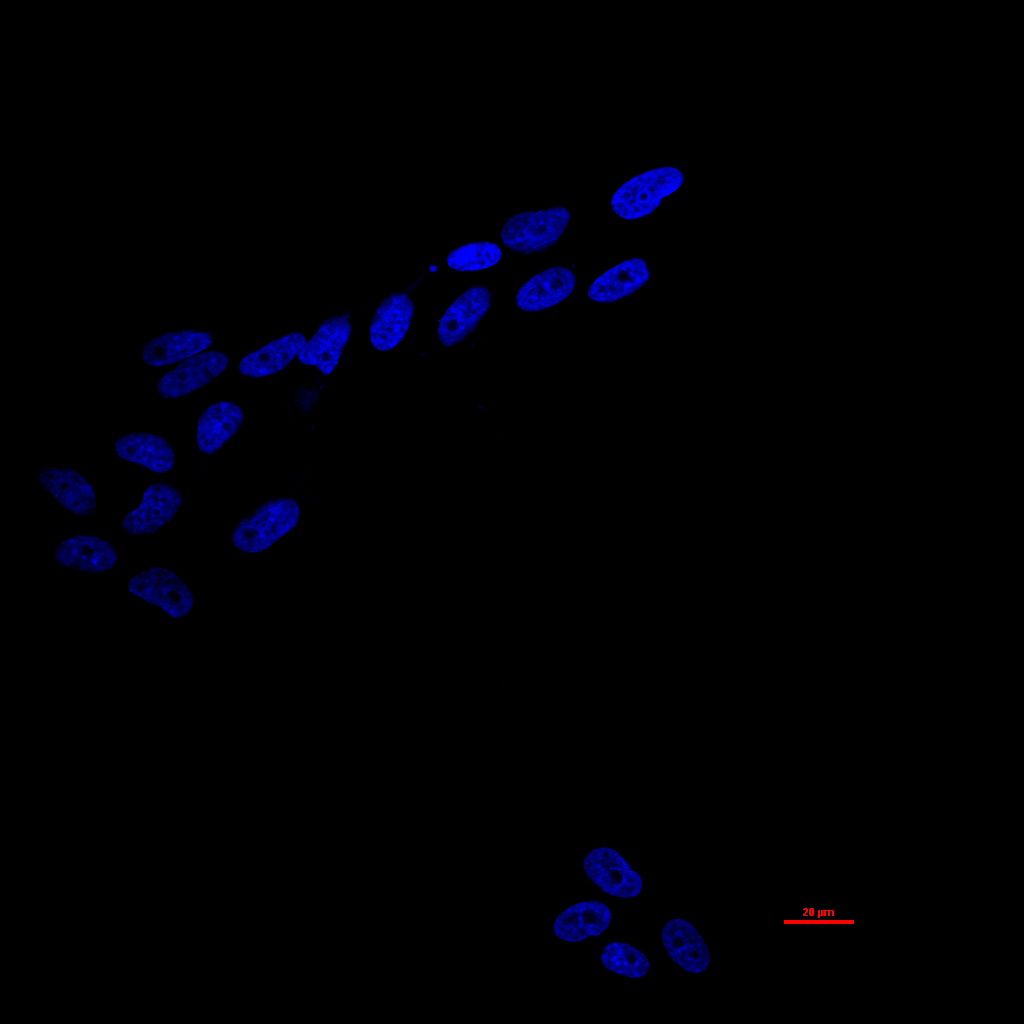

Supplement: Supplementary file 1 [file jox-15-00137-s001.zip › File S1/Figure 5. SIRT1 siRNA + SiNPs+ DAPI.tif]

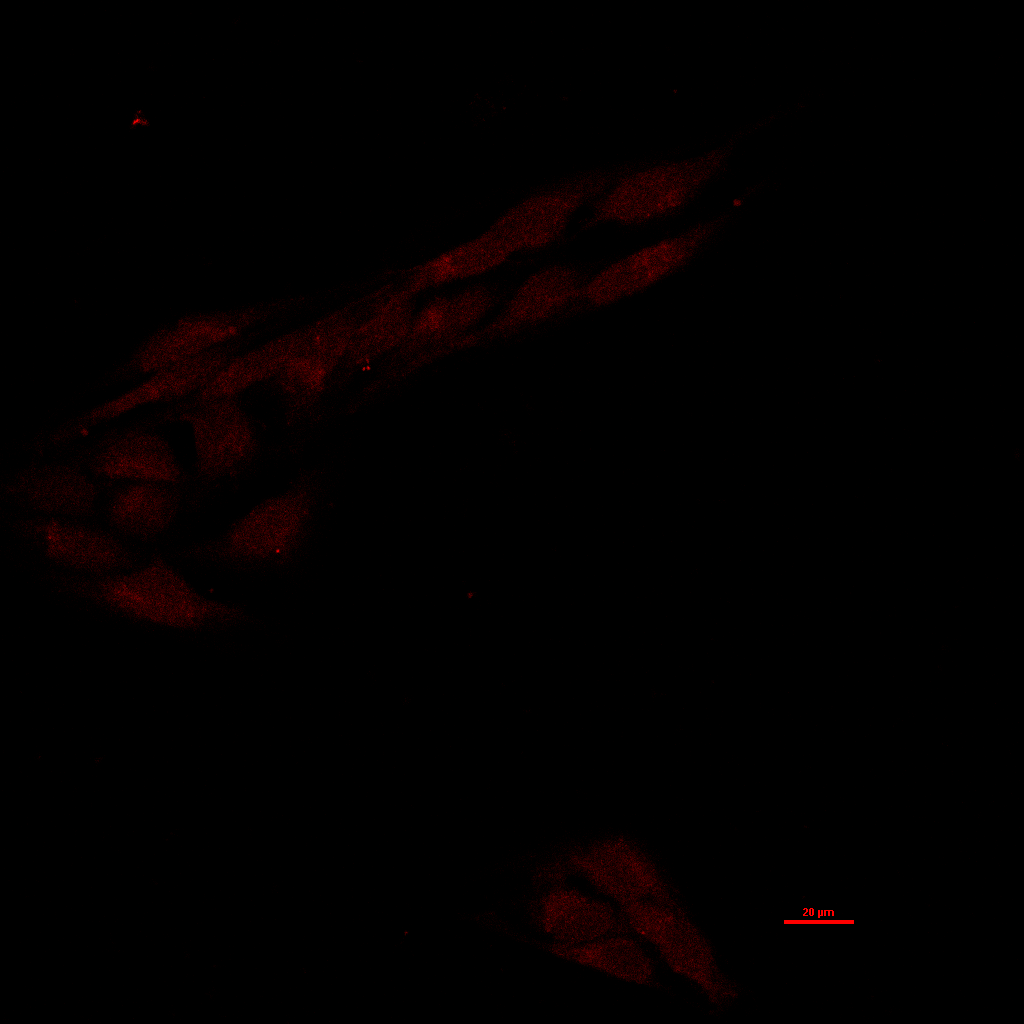

Supplement: Supplementary file 1 [file jox-15-00137-s001.zip › File S1/Figure 5. SIRT1 siRNA + SiNPs+ HSF1.tif]

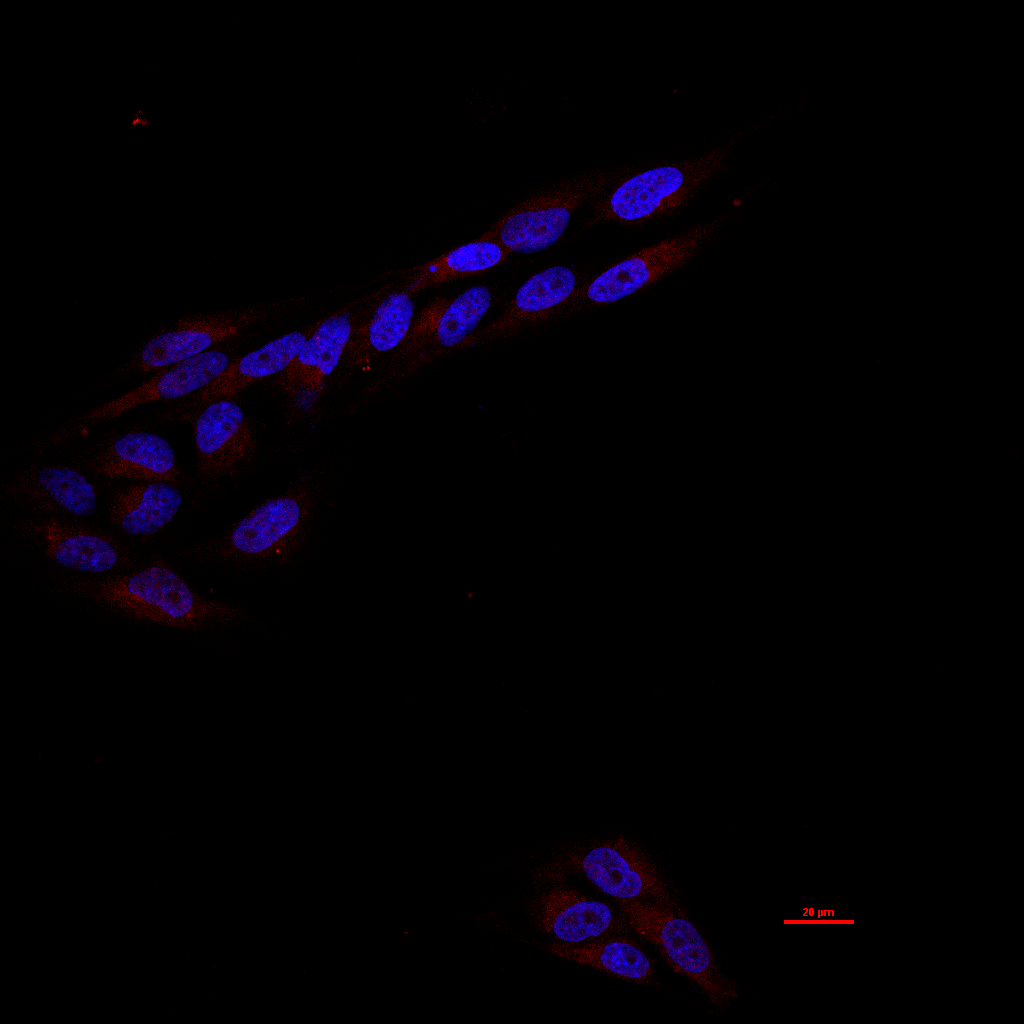

Supplement: Supplementary file 1 [file jox-15-00137-s001.zip › File S1/Figure 5. SIRT1 siRNA + SiNPs+ Merge.tif]

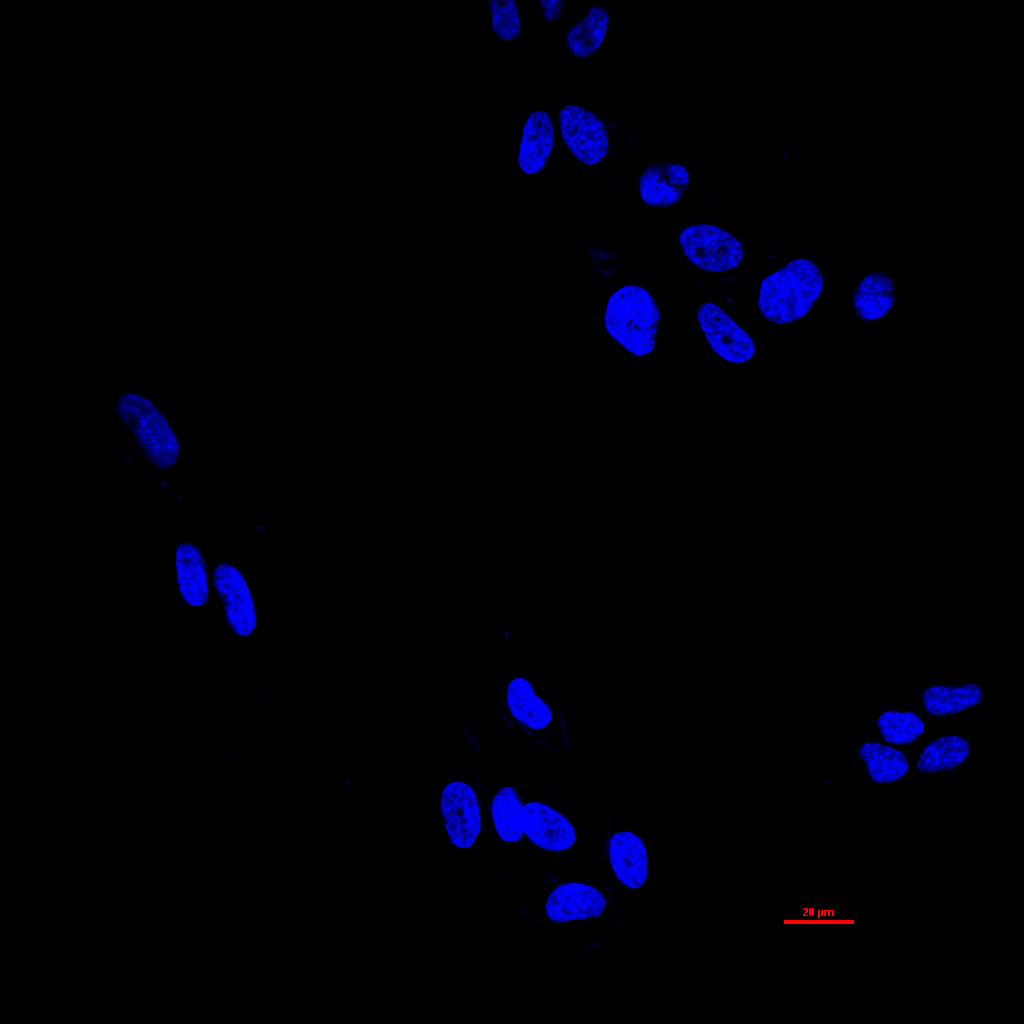

Supplement: Supplementary file 1 [file jox-15-00137-s001.zip › File S1/Figure 5. SIRT1 siRNA+ SiNPs- DAPI.tif]

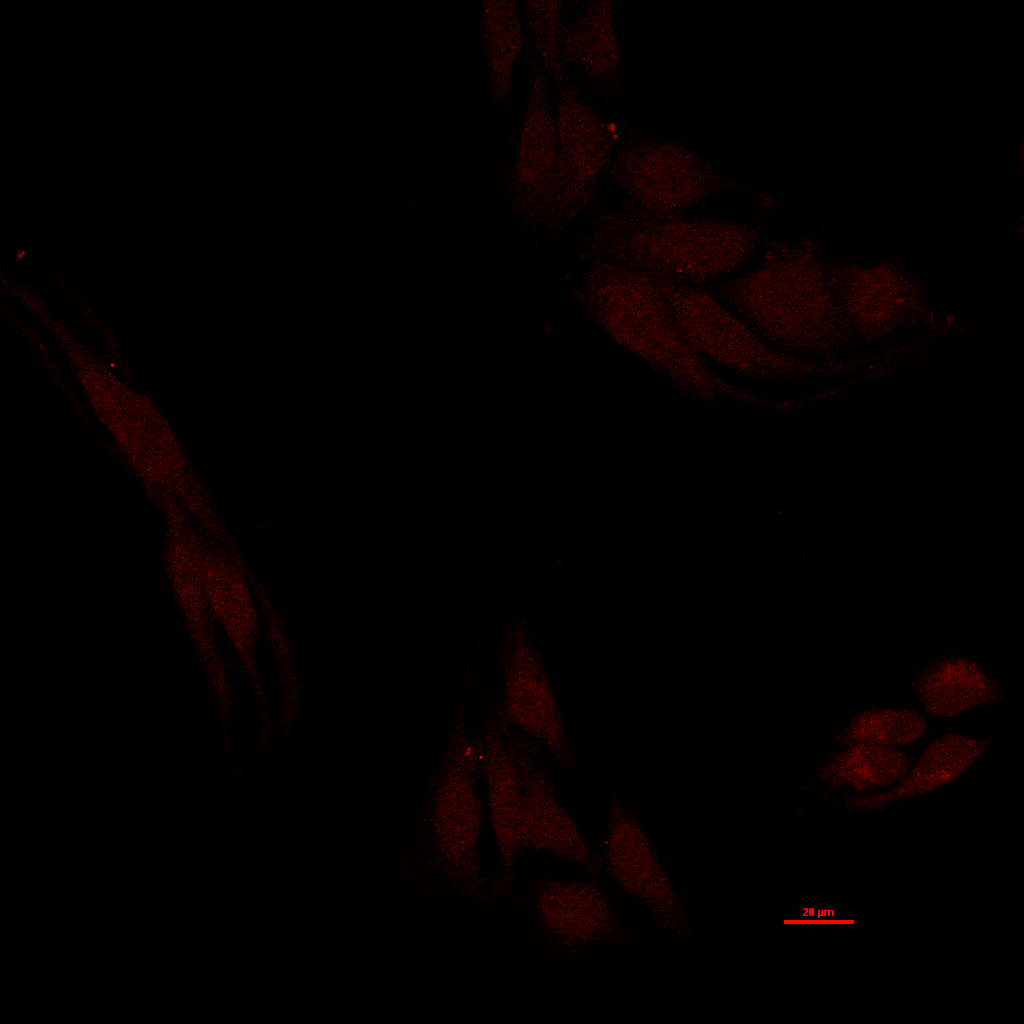

Supplement: Supplementary file 1 [file jox-15-00137-s001.zip › File S1/Figure 5. SIRT1 siRNA+ SiNPs- HSF1.tif]

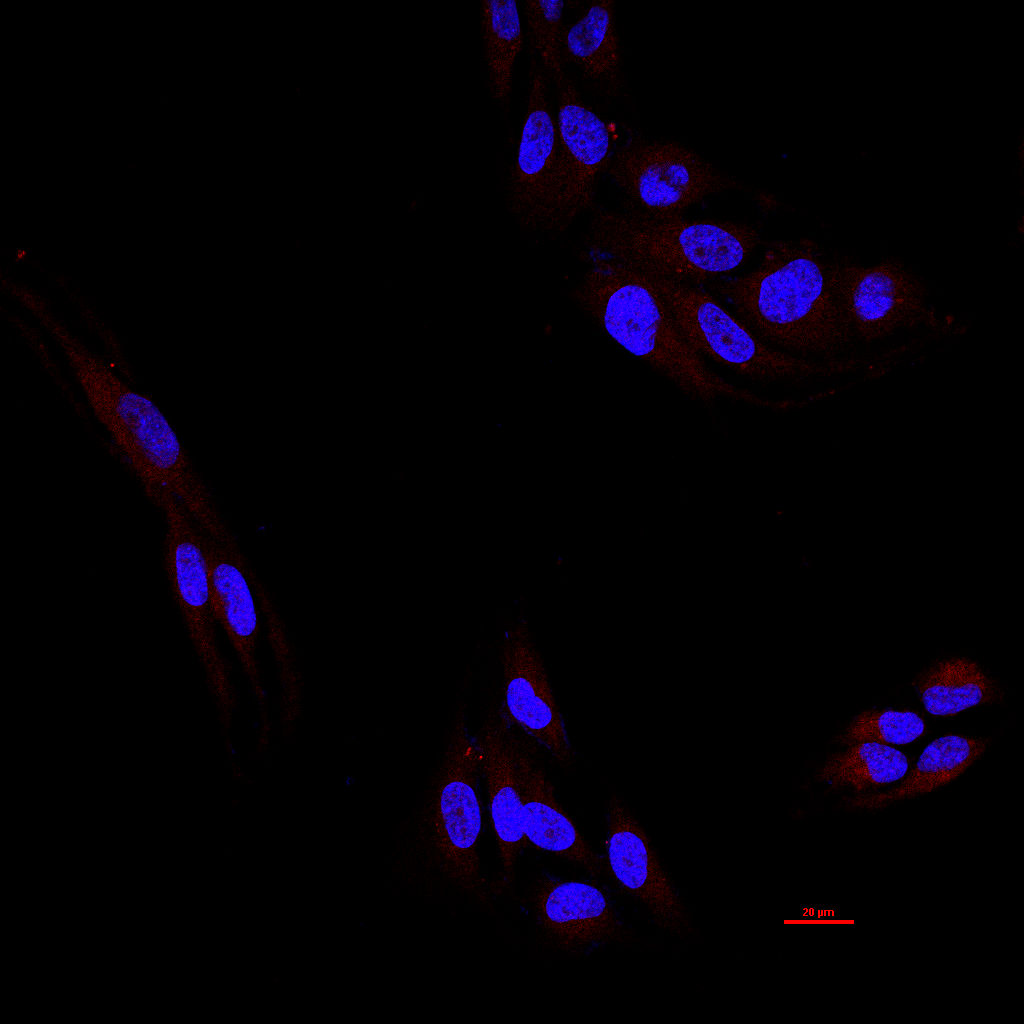

Supplement: Supplementary file 1 [file jox-15-00137-s001.zip › File S1/Figure 5. SIRT1 siRNA+ SiNPs- Merge.tif]

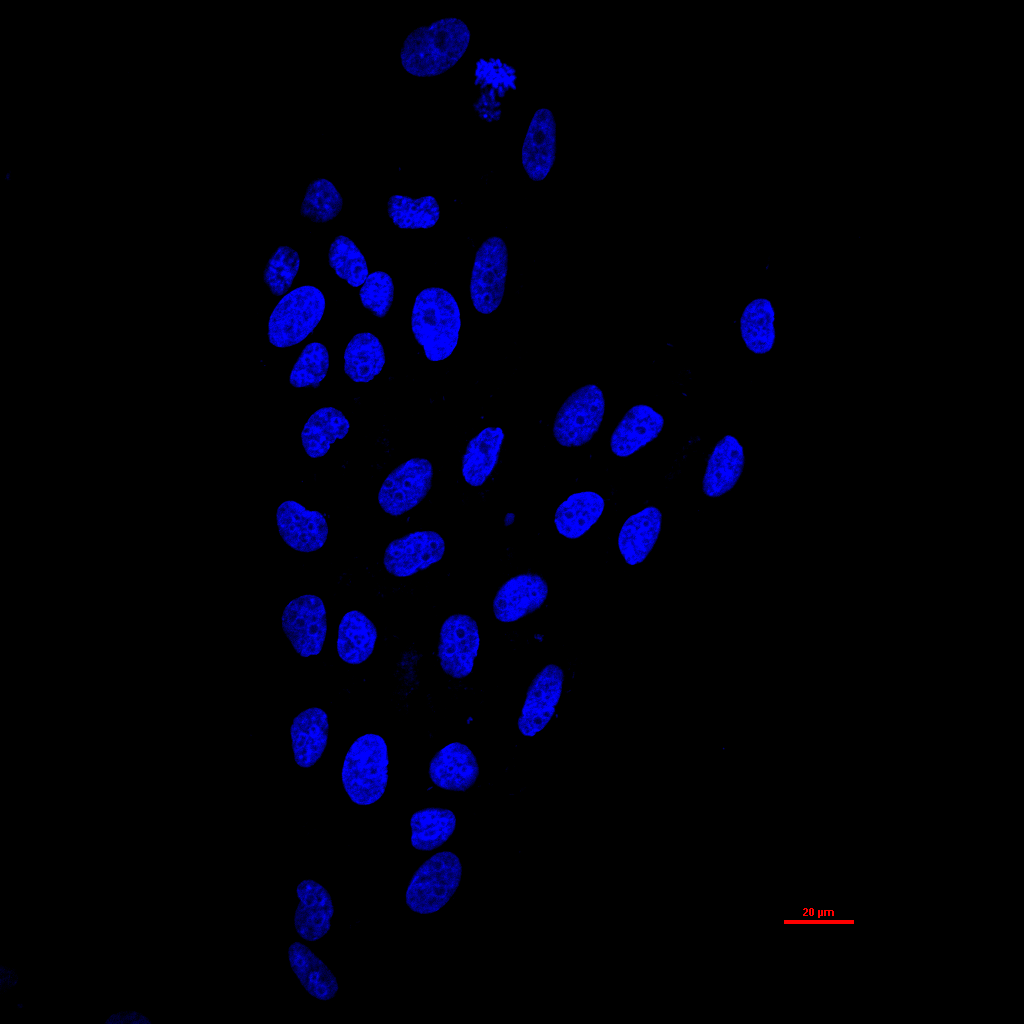

Supplement: Supplementary file 1 [file jox-15-00137-s001.zip › File S1/Figure 5. SIRT1 siRNA- SiNPs+ DAPI.tif]

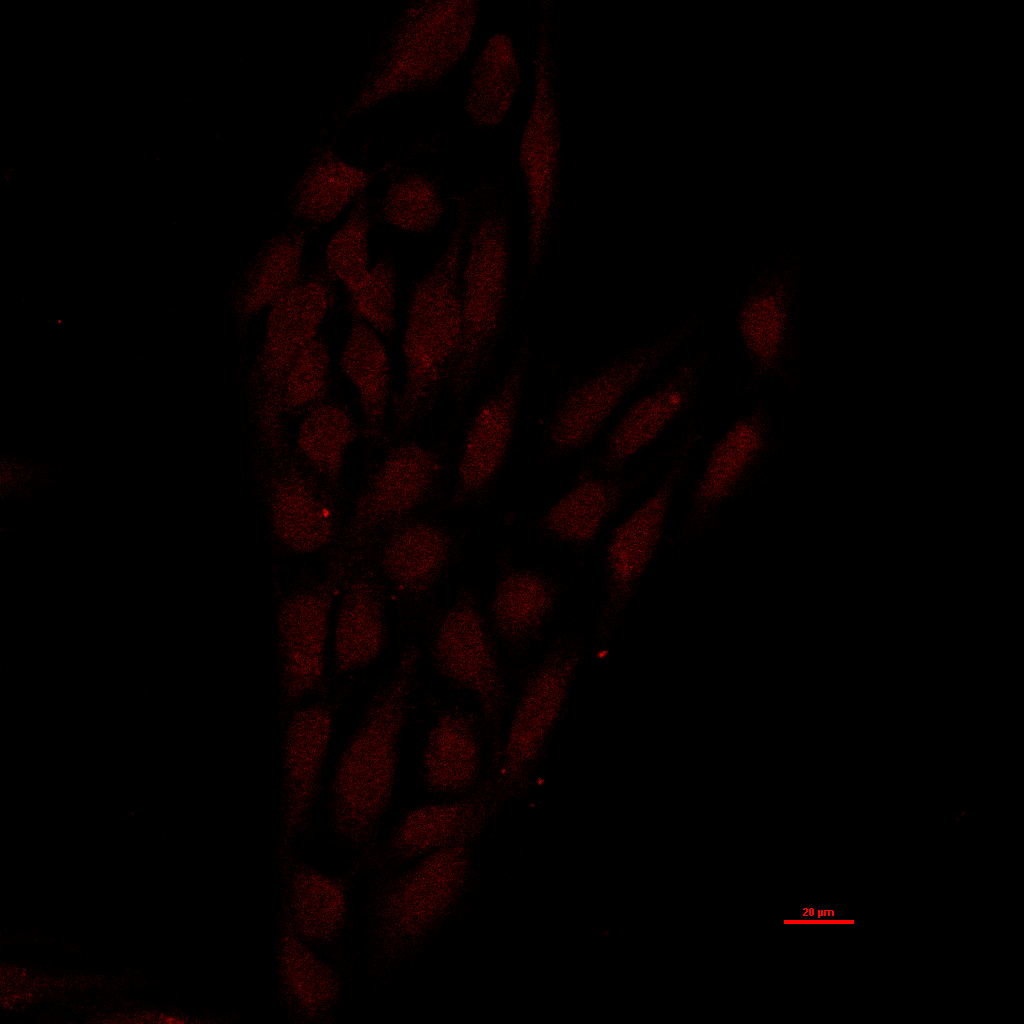

Supplement: Supplementary file 1 [file jox-15-00137-s001.zip › File S1/Figure 5. SIRT1 siRNA- SiNPs+ HSF1.tif]

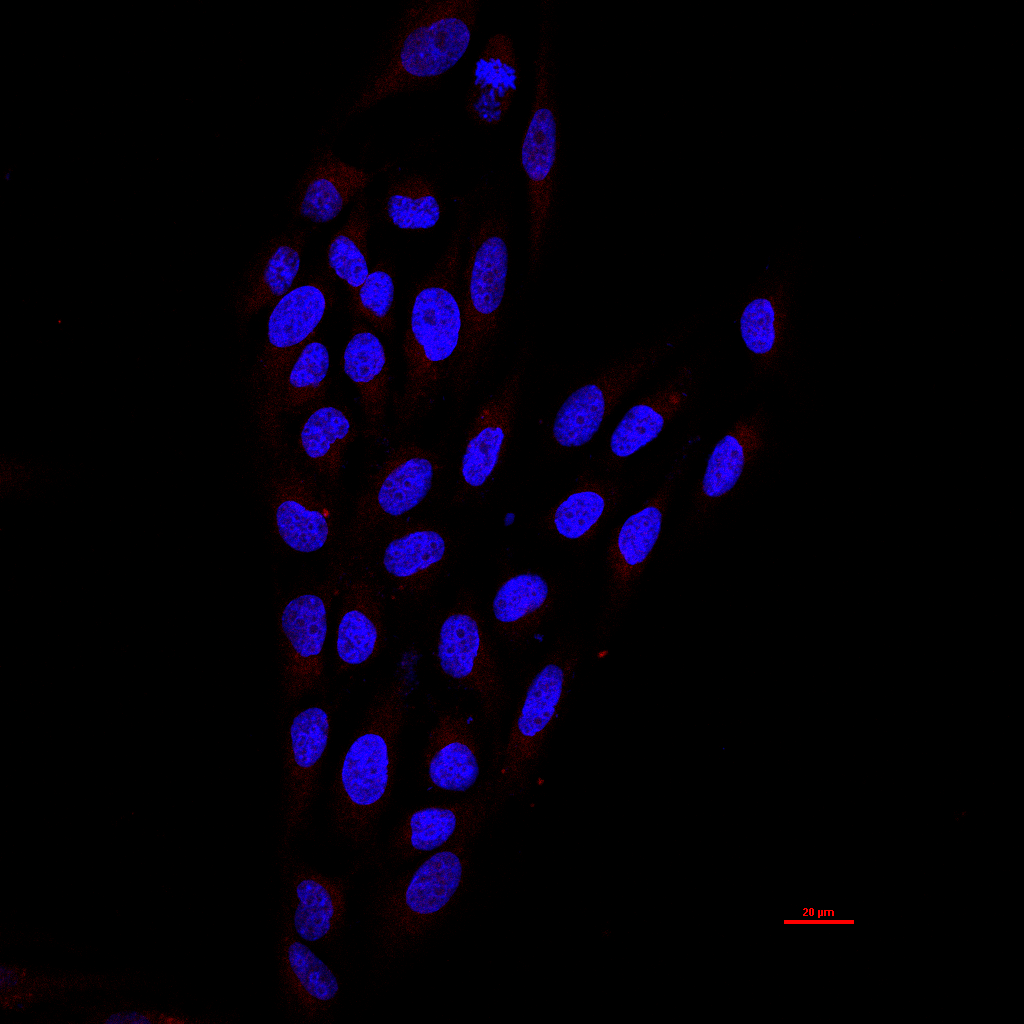

Supplement: Supplementary file 1 [file jox-15-00137-s001.zip › File S1/Figure 5. SIRT1 siRNA- SiNPs+ Merge.tif]

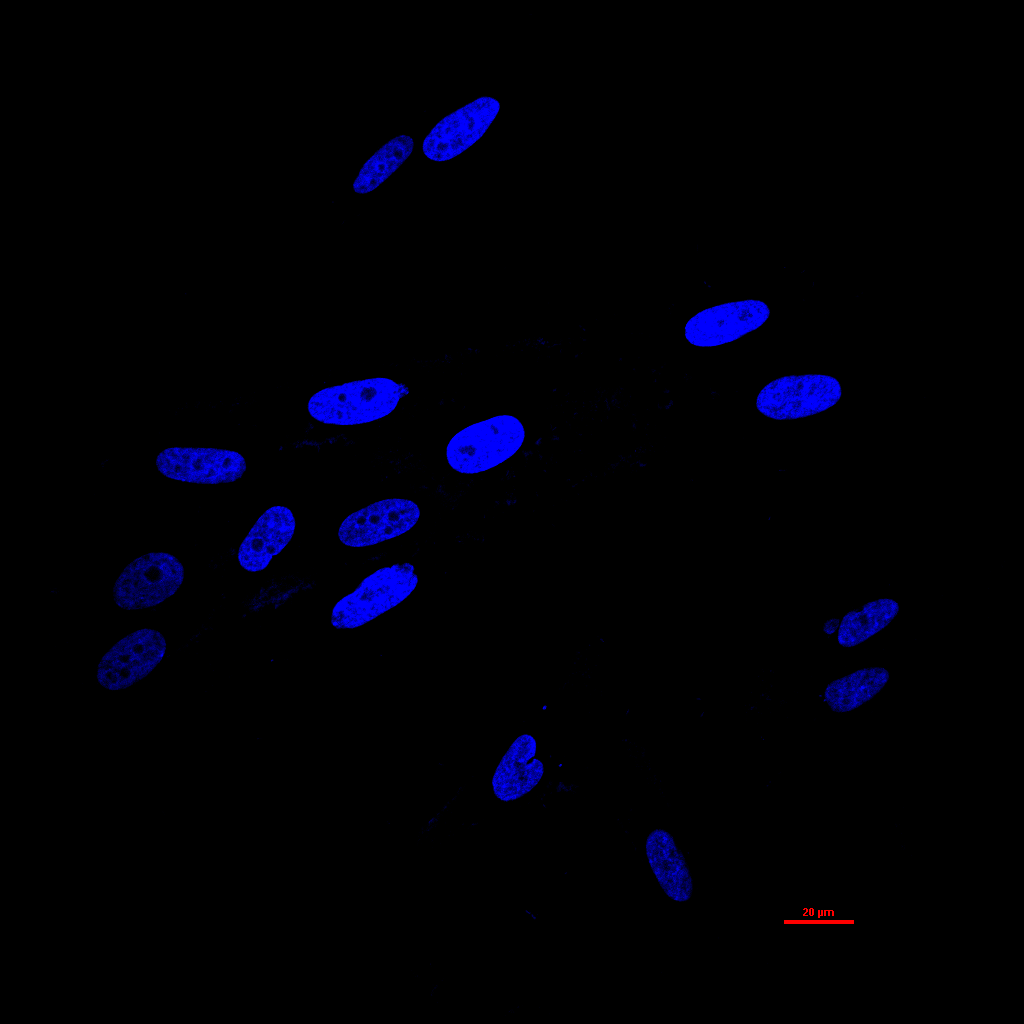

Supplement: Supplementary file 1 [file jox-15-00137-s001.zip › File S1/Figure 5. SIRT1 siRNA- SiNPs- DAPI.tif]

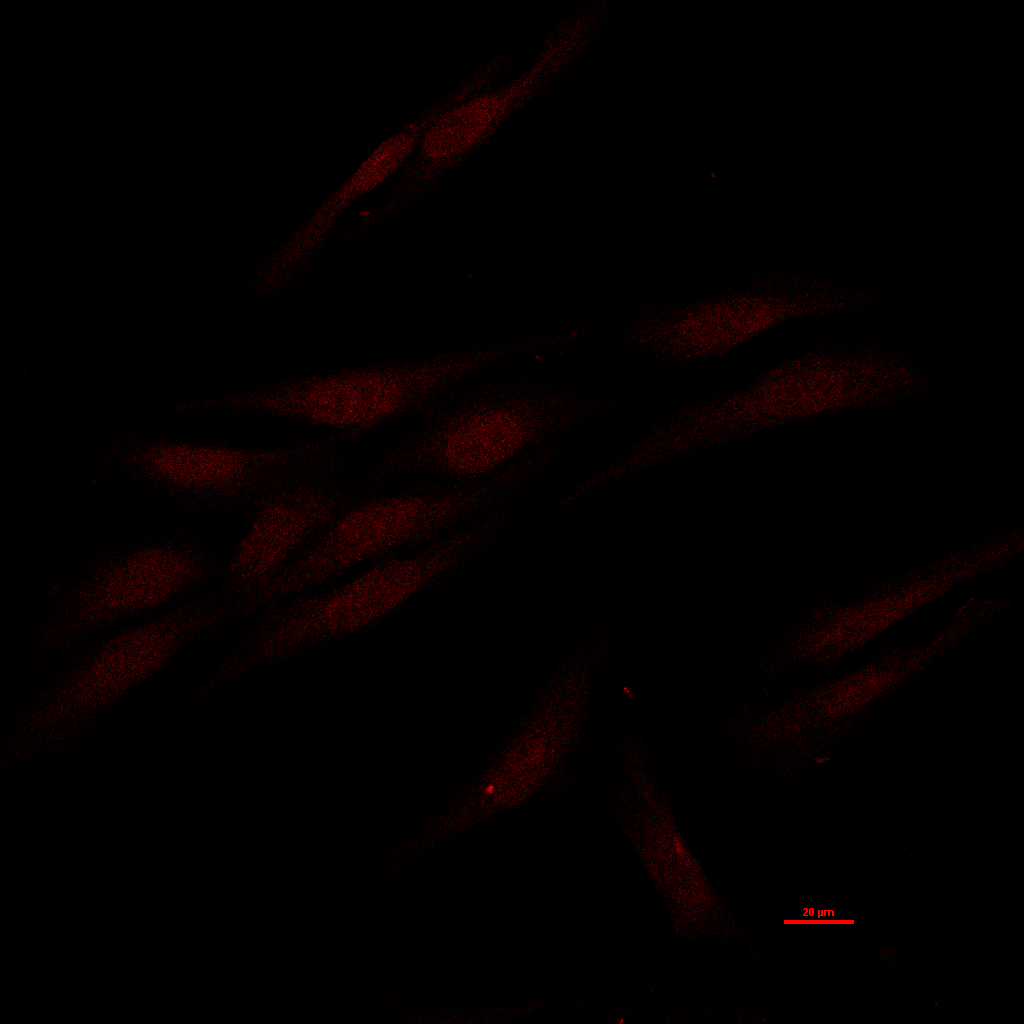

Supplement: Supplementary file 1 [file jox-15-00137-s001.zip › File S1/Figure 5. SIRT1 siRNA- SiNPs- HSF1.tif]

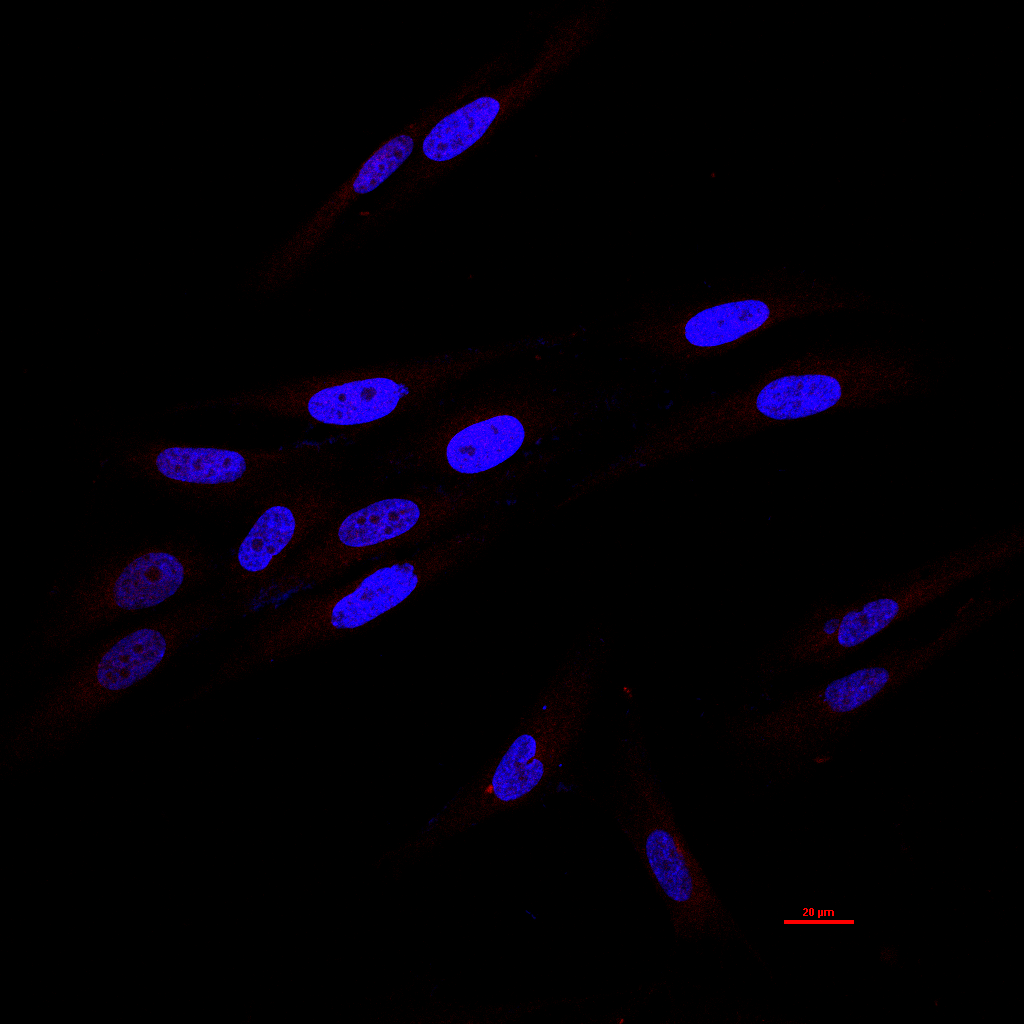

Supplement: Supplementary file 1 [file jox-15-00137-s001.zip › File S1/Figure 5. SIRT1 siRNA- SiNPs- Merge.tif]

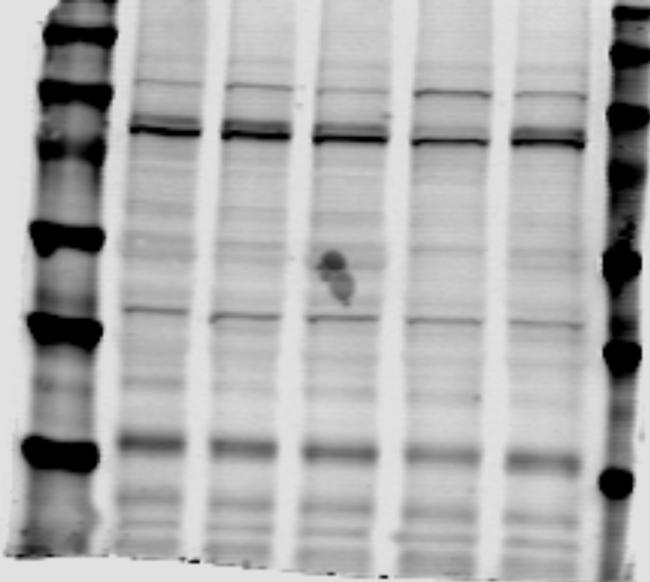

Supplement: Supplementary file 1 [file jox-15-00137-s001.zip › File S1/Figure 6. A) E-cadherin.tif]

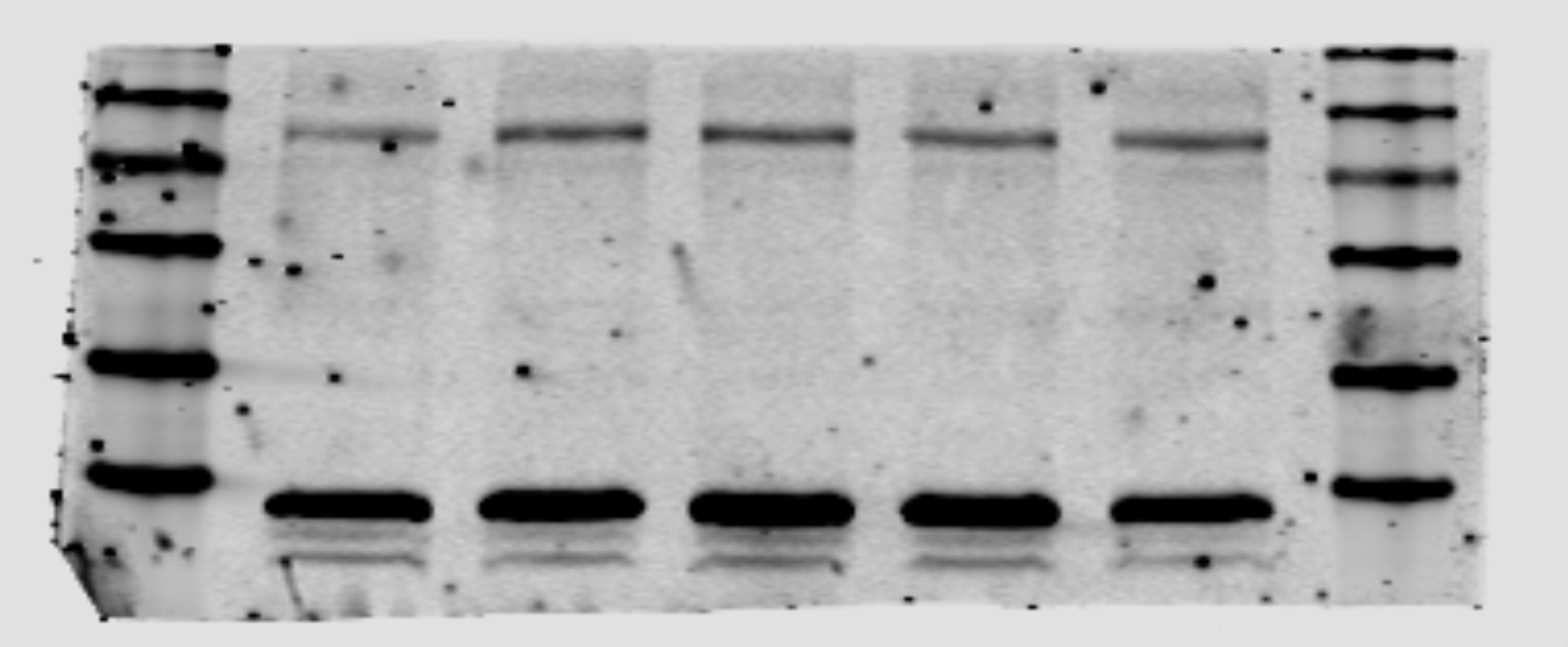

Supplement: Supplementary file 1 [file jox-15-00137-s001.zip › File S1/Figure 6. A) GAPDH.tif]

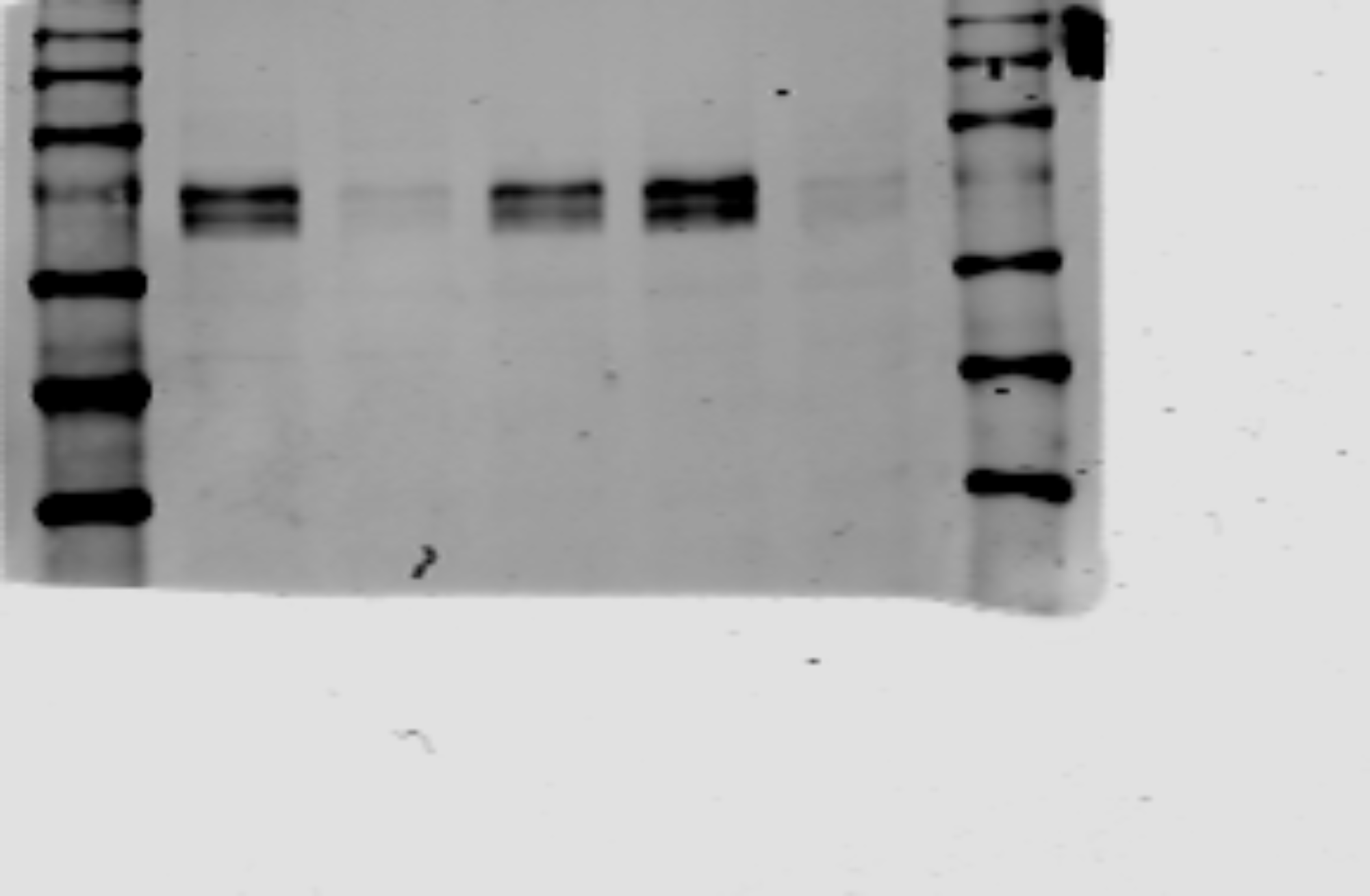

Supplement: Supplementary file 1 [file jox-15-00137-s001.zip › File S1/Figure 6. A) HSF1.tif]

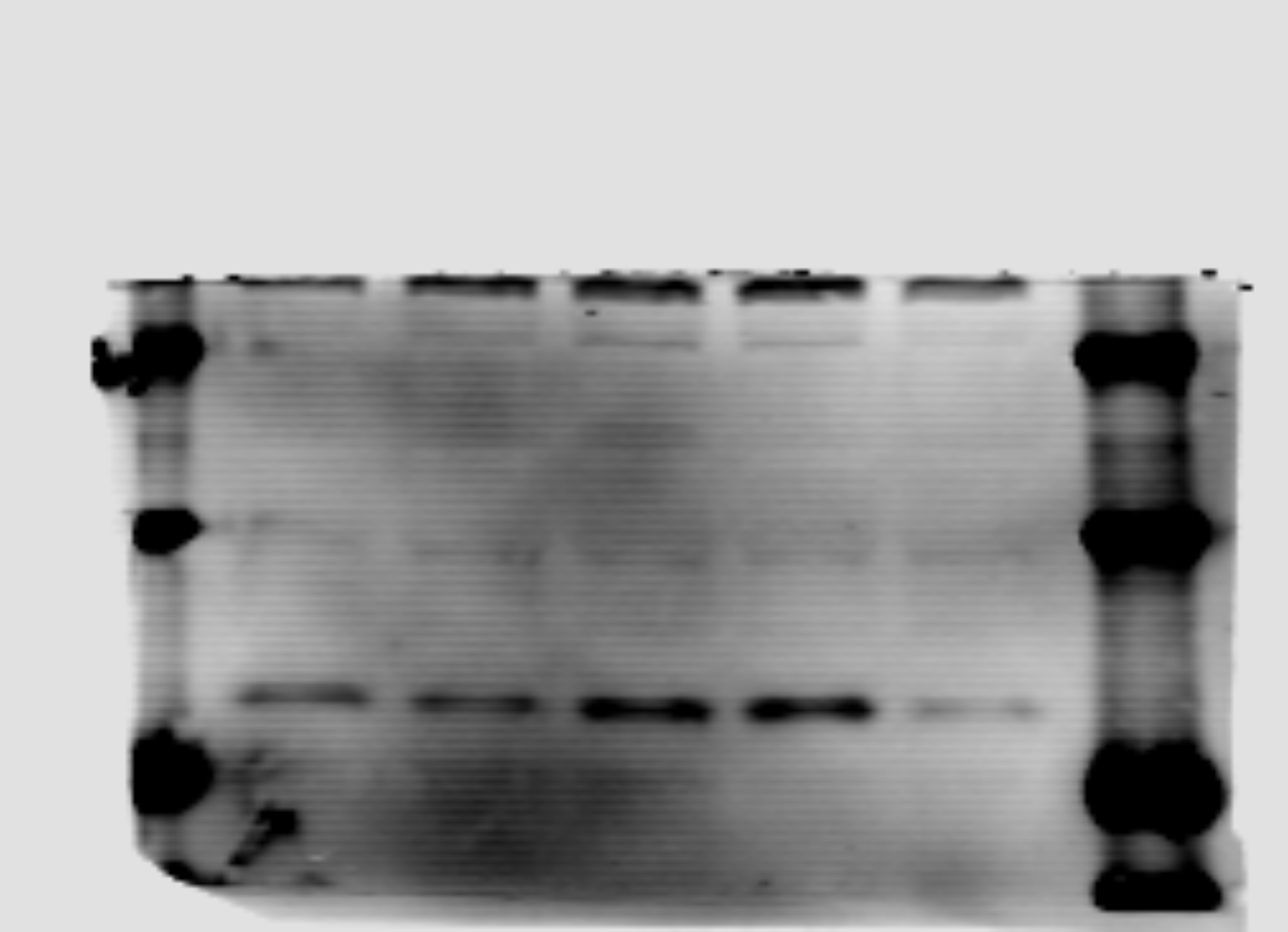

Supplement: Supplementary file 1 [file jox-15-00137-s001.zip › File S1/Figure 6. A) HSP27.tif]

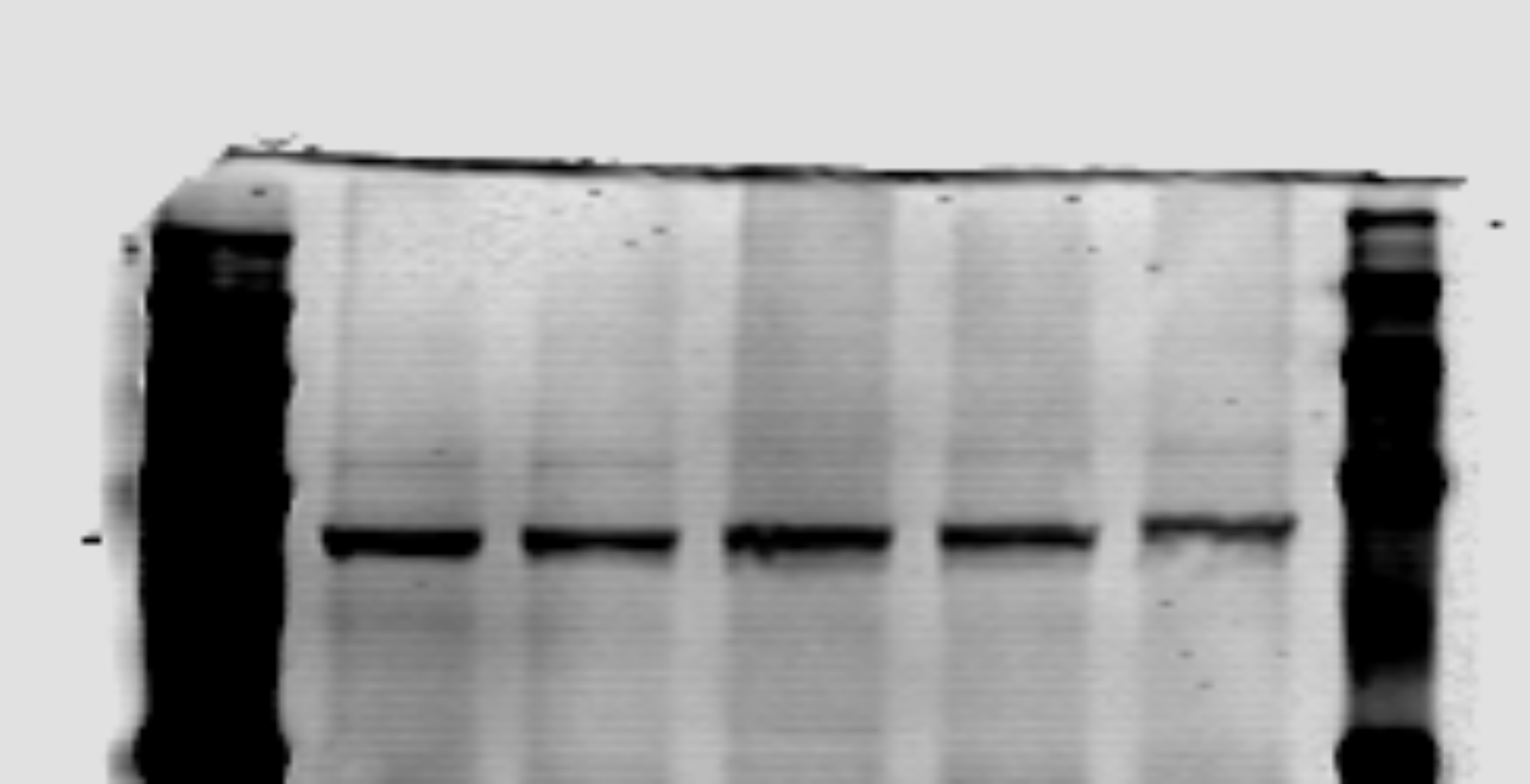

Supplement: Supplementary file 1 [file jox-15-00137-s001.zip › File S1/Figure 6. A) HSP90.tif]

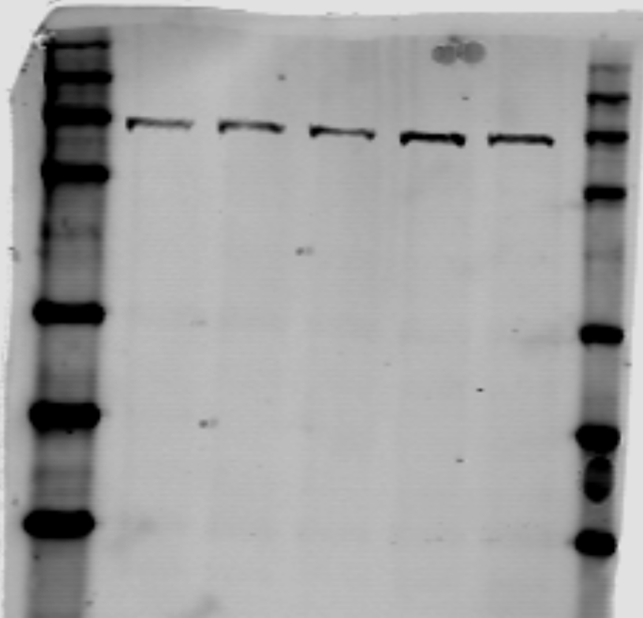

Supplement: Supplementary file 1 [file jox-15-00137-s001.zip › File S1/Figure 6. A) N-cadherin.tif]

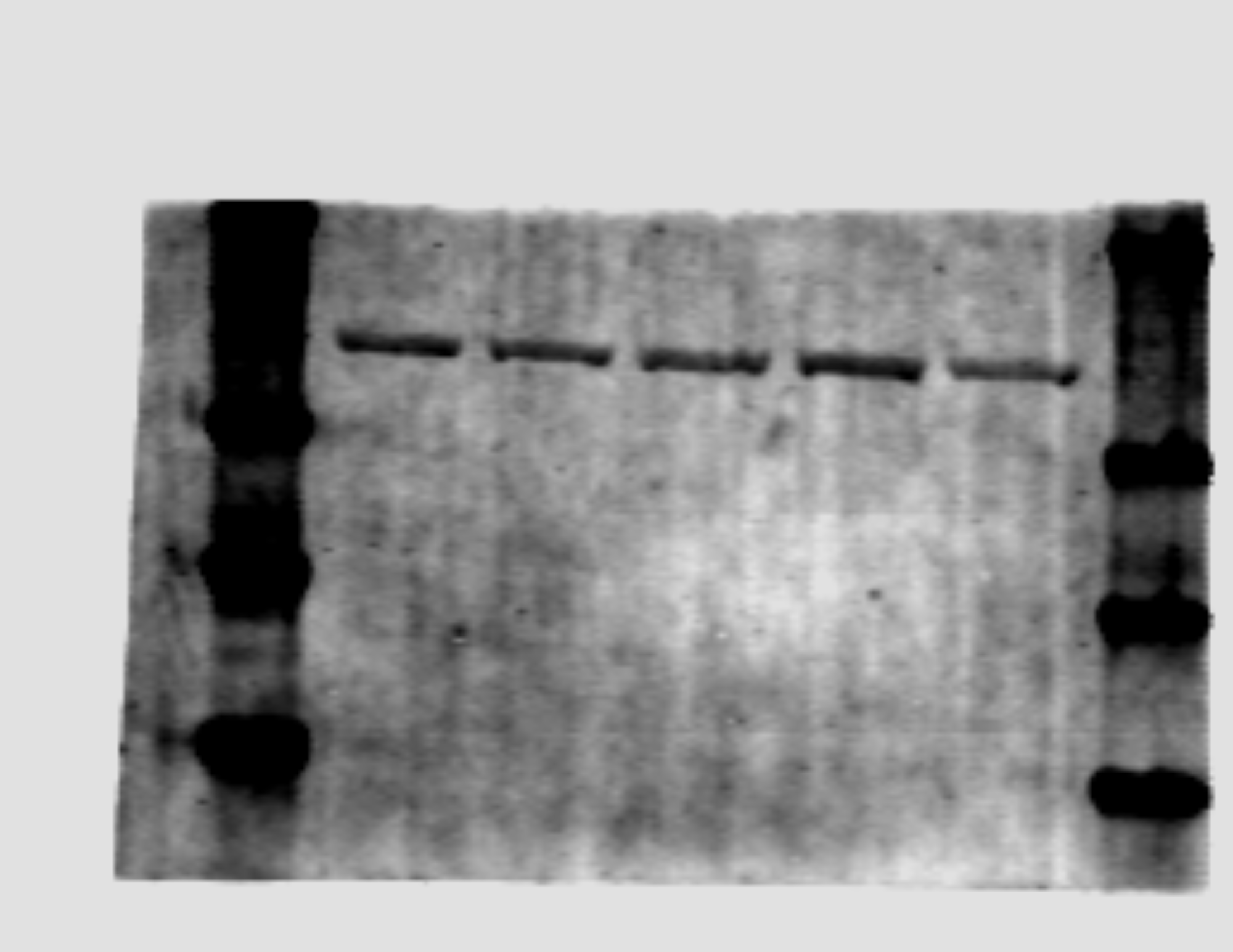

Supplement: Supplementary file 1 [file jox-15-00137-s001.zip › File S1/Figure 6. A)HSP70.tif]

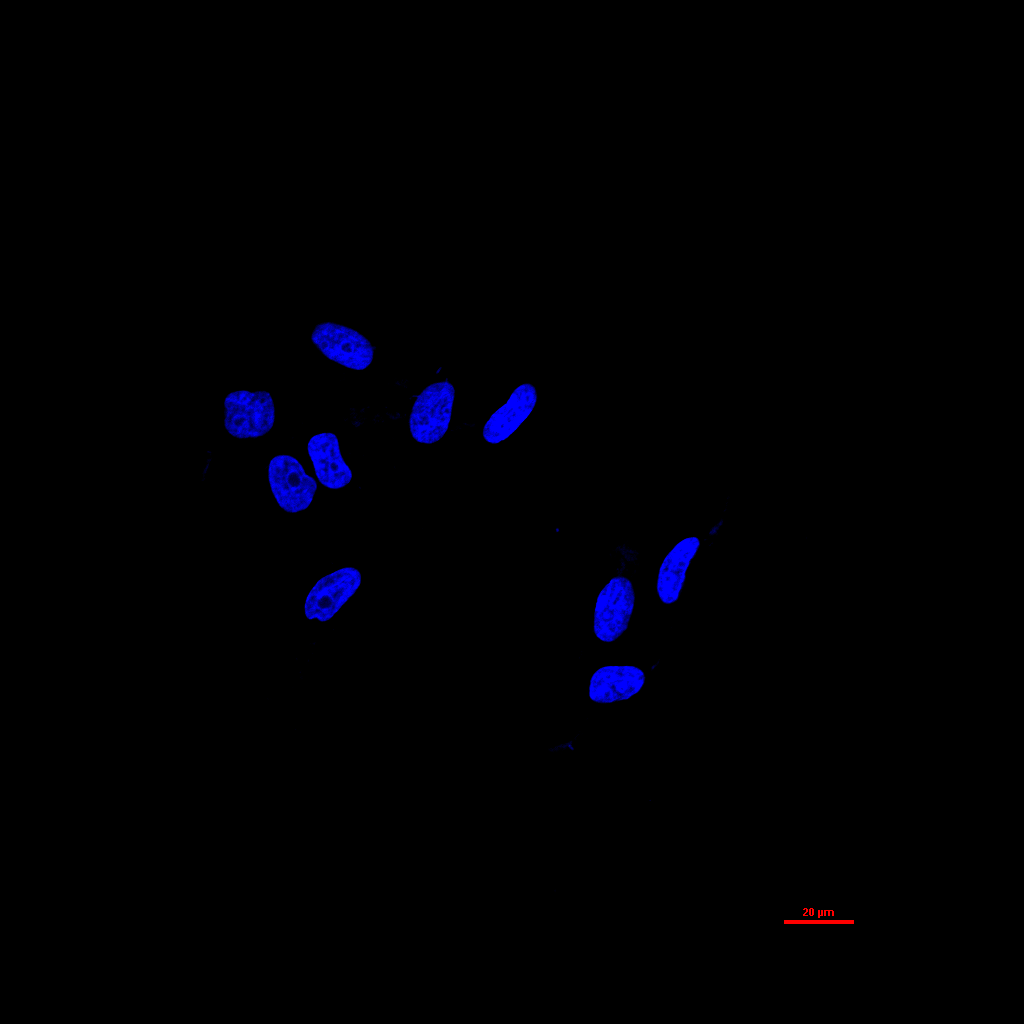

Supplement: Supplementary file 1 [file jox-15-00137-s001.zip › File S1/Figure 6. B) HSF1 siRNA+ SiNPs+ DAPI.tif]

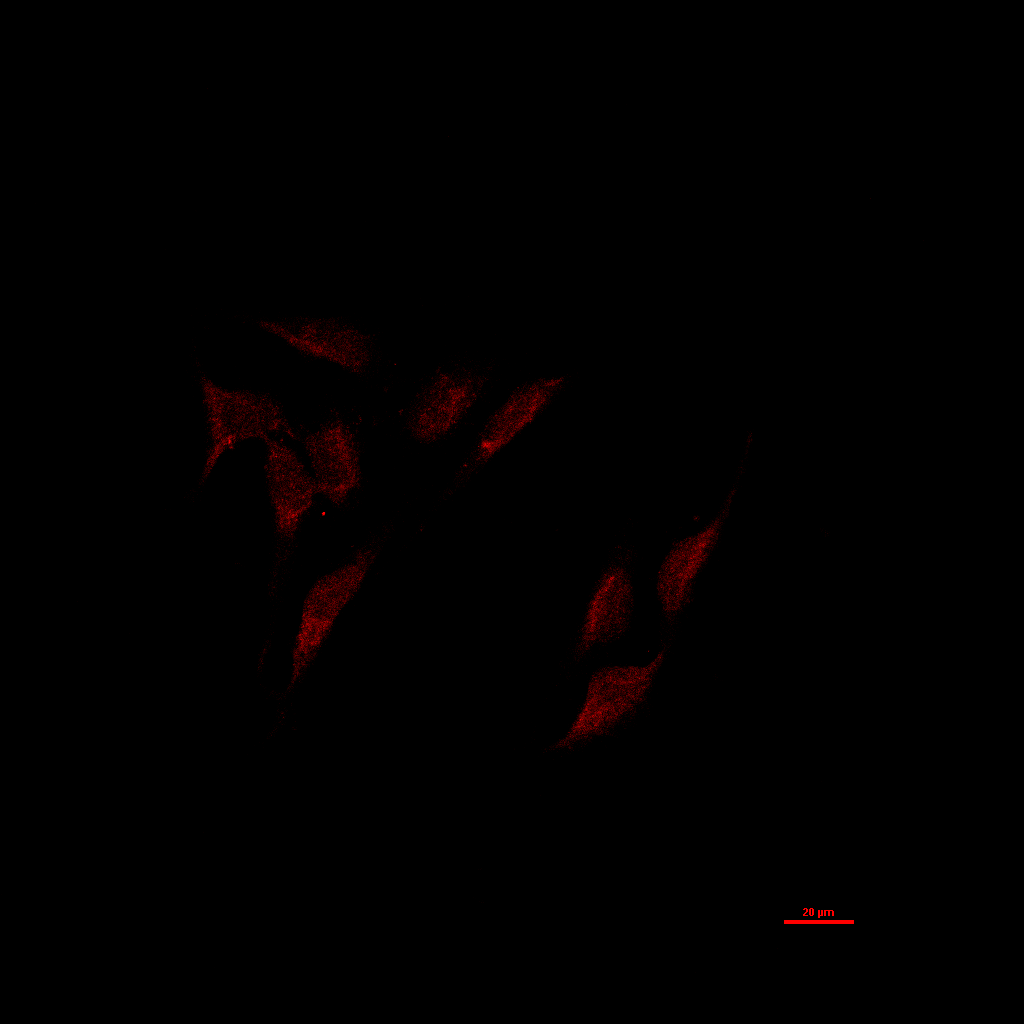

Supplement: Supplementary file 1 [file jox-15-00137-s001.zip › File S1/Figure 6. B) HSF1 siRNA+ SiNPs+ HSF1.tif]

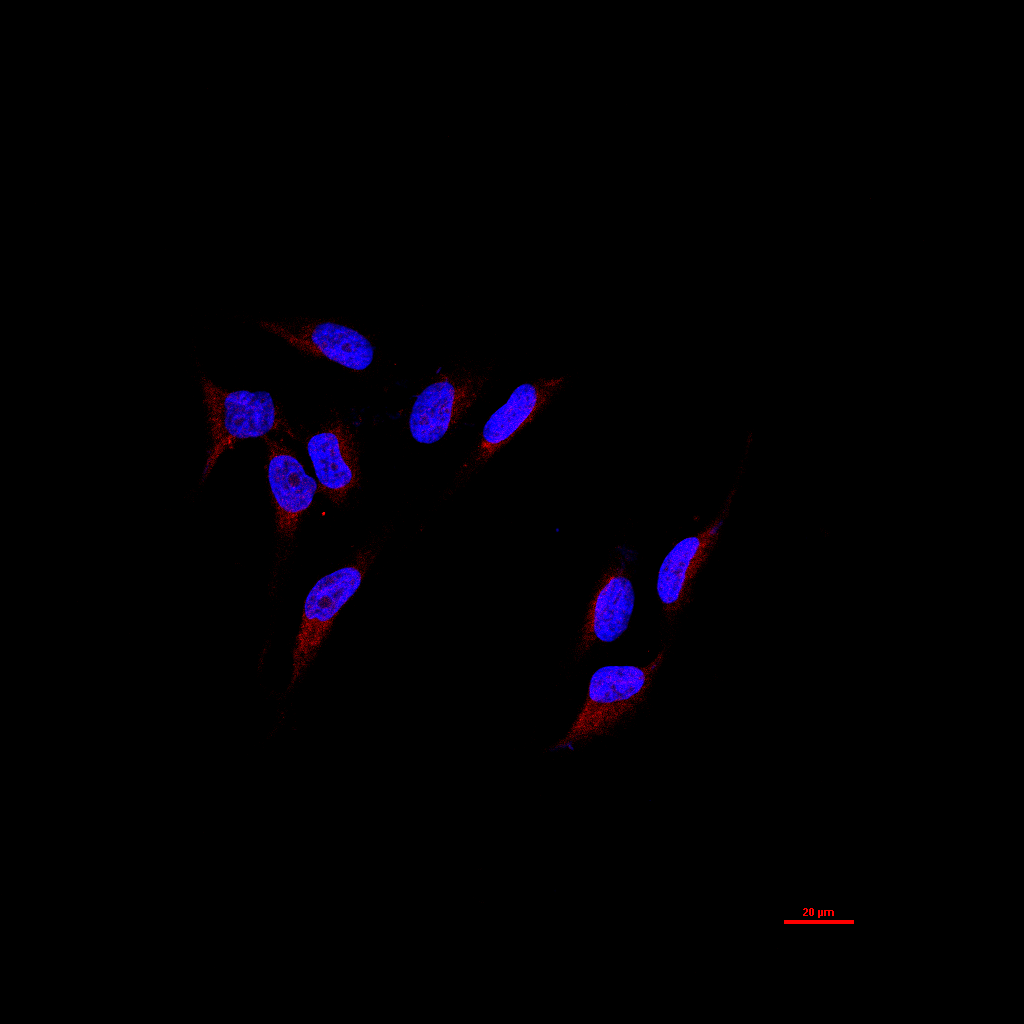

Supplement: Supplementary file 1 [file jox-15-00137-s001.zip › File S1/Figure 6. B) HSF1 siRNA+ SiNPs+ Merge.tif]

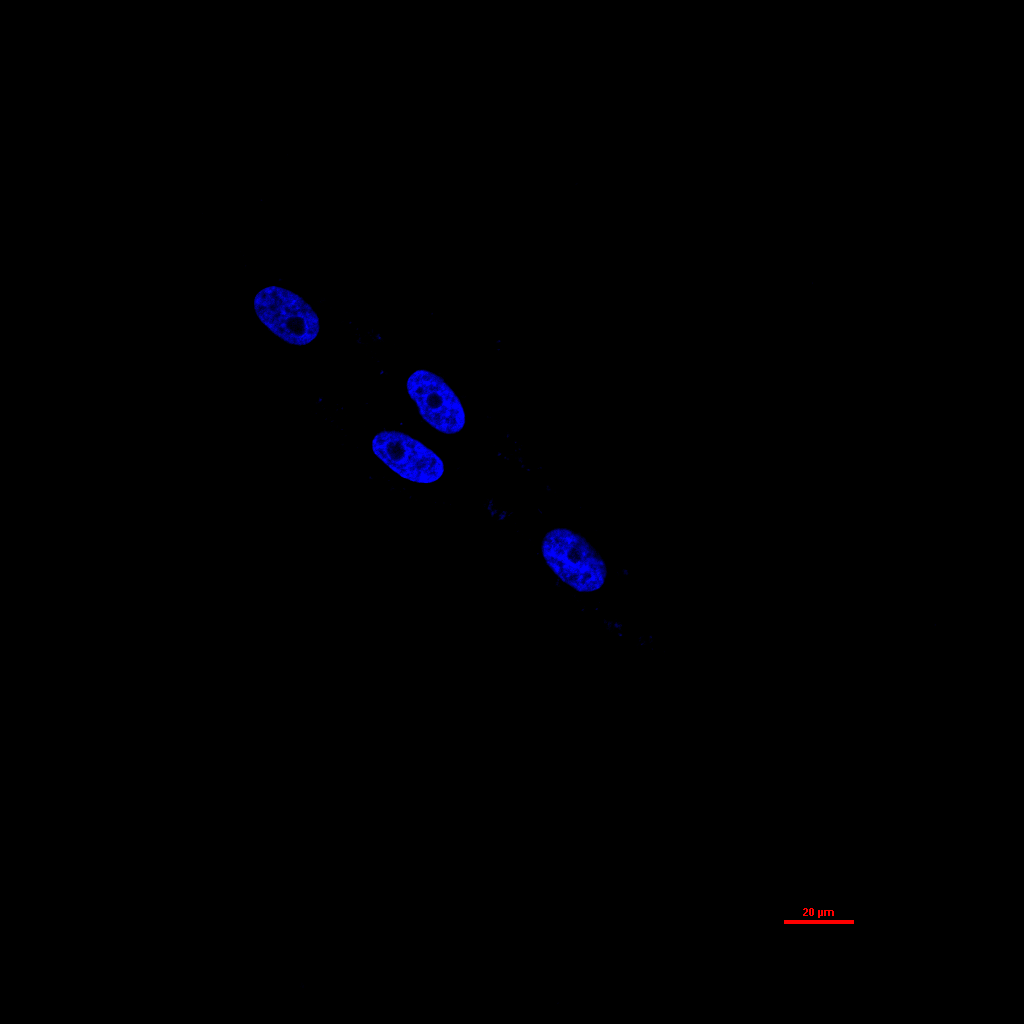

Supplement: Supplementary file 1 [file jox-15-00137-s001.zip › File S1/Figure 6. B) HSF1 siRNA+ SiNPs- DAPI.tif]

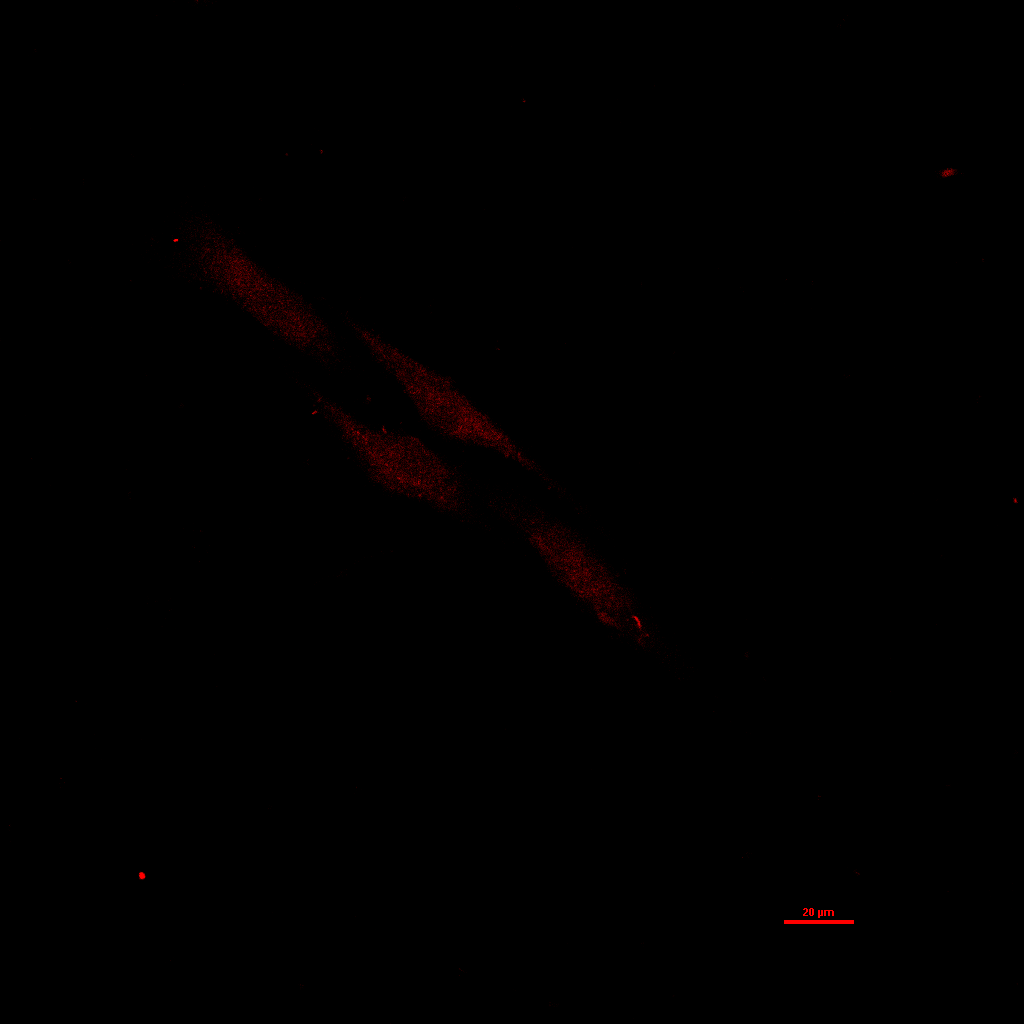

Supplement: Supplementary file 1 [file jox-15-00137-s001.zip › File S1/Figure 6. B) HSF1 siRNA+ SiNPs- HSF1.tif]

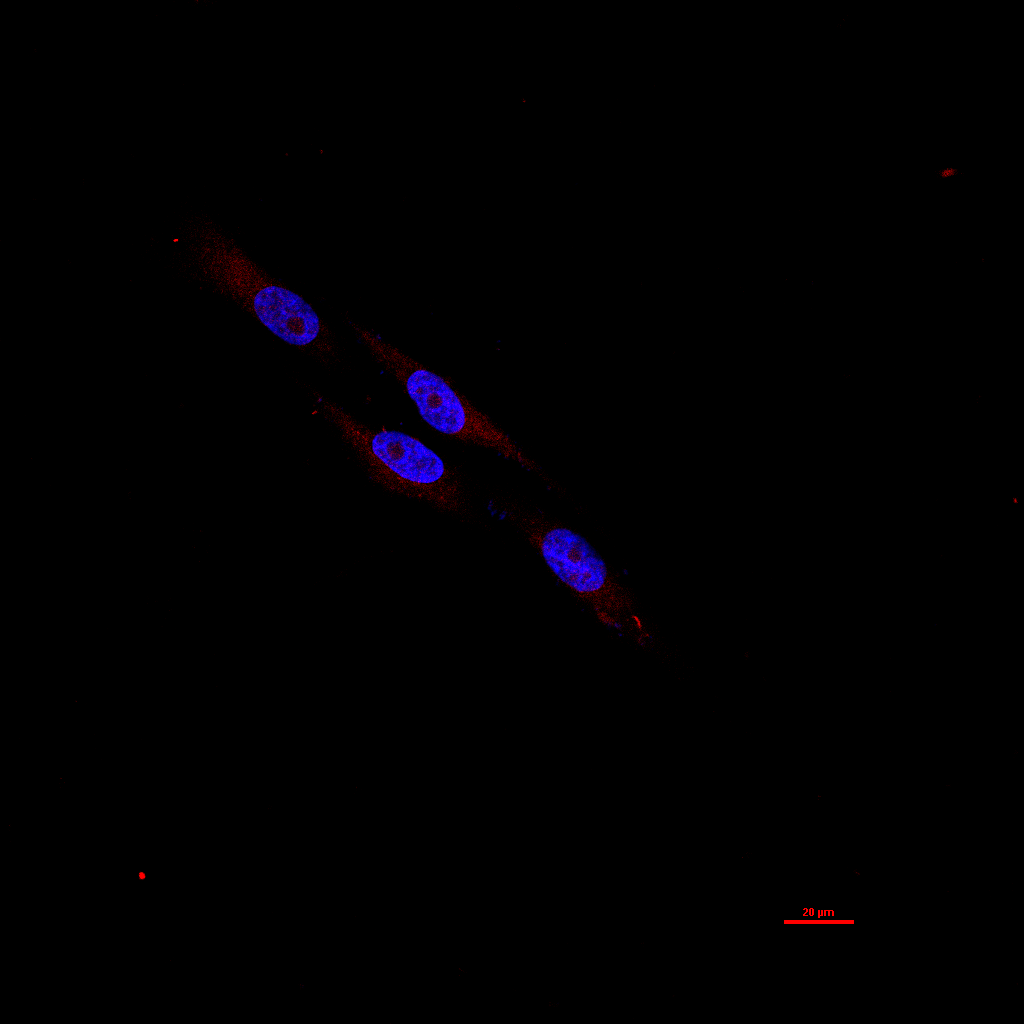

Supplement: Supplementary file 1 [file jox-15-00137-s001.zip › File S1/Figure 6. B) HSF1 siRNA+ SiNPs- Merge.tif]

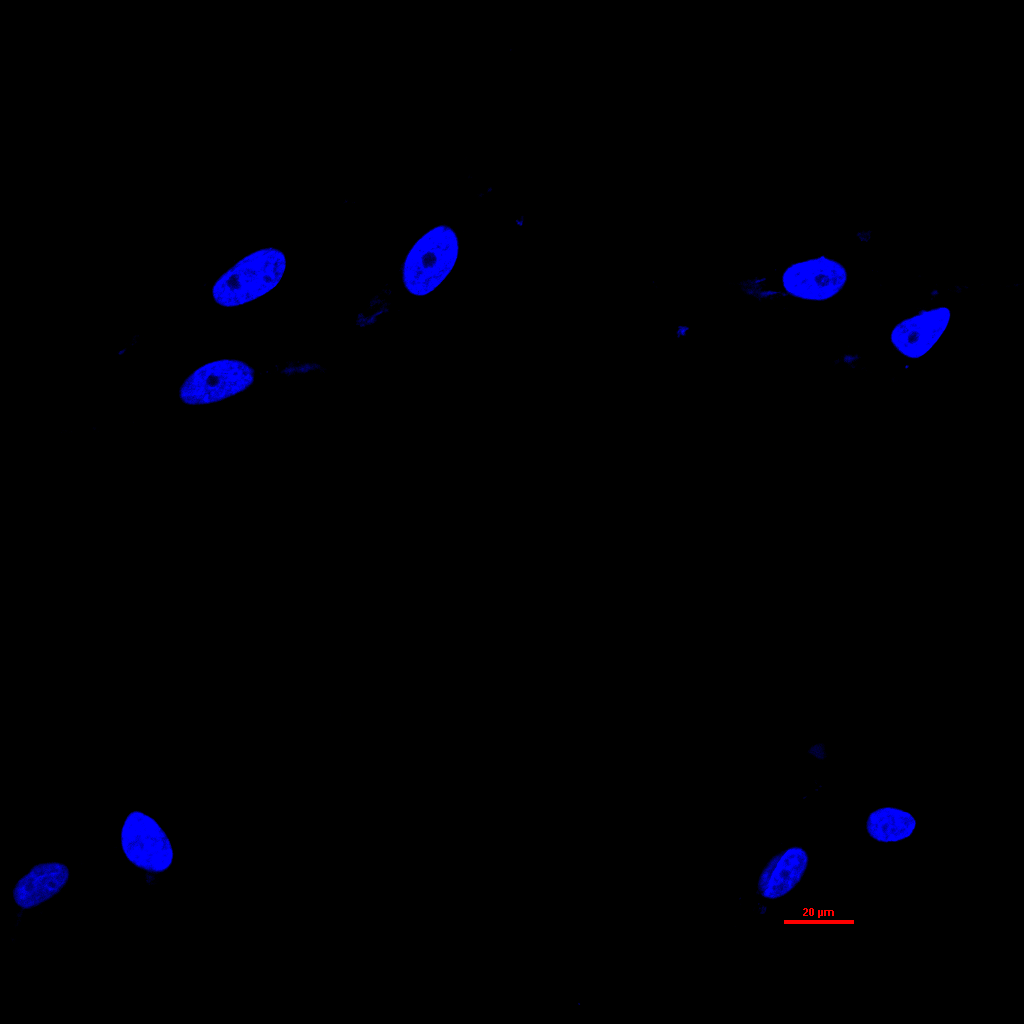

Supplement: Supplementary file 1 [file jox-15-00137-s001.zip › File S1/Figure 6. B) HSF1 siRNA- SiNPs- DAPI.tif]

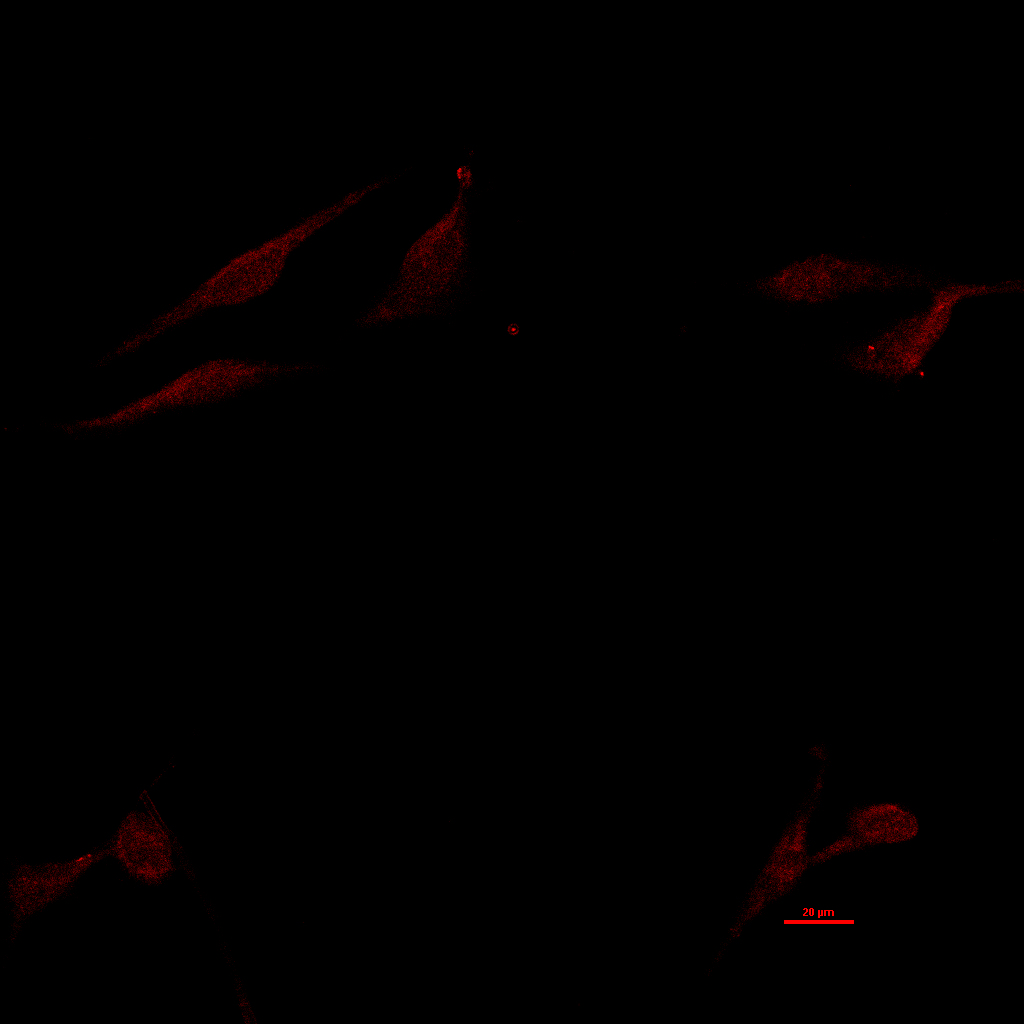

Supplement: Supplementary file 1 [file jox-15-00137-s001.zip › File S1/Figure 6. B) HSF1 siRNA- SiNPs- HSF1.tif]

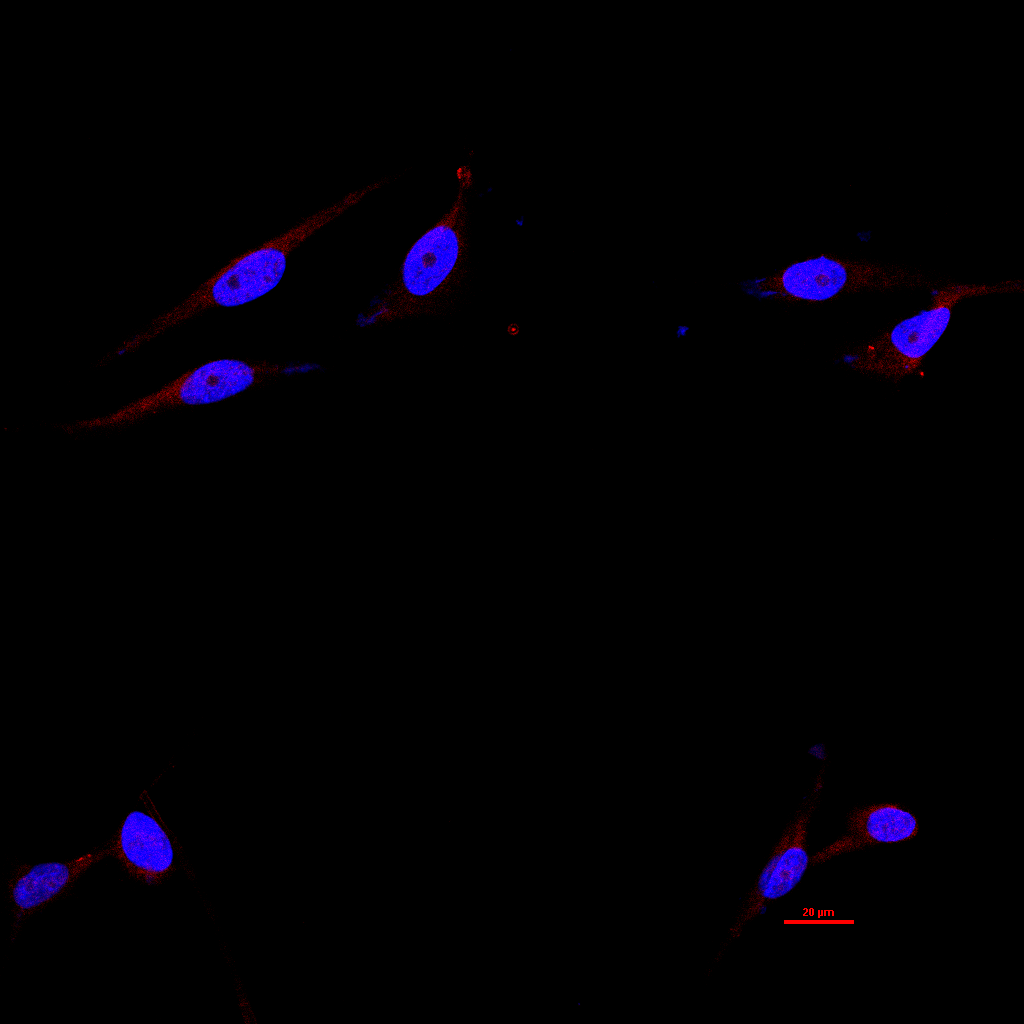

Supplement: Supplementary file 1 [file jox-15-00137-s001.zip › File S1/Figure 6. B) HSF1 siRNA- SiNPs- Merge.tif]

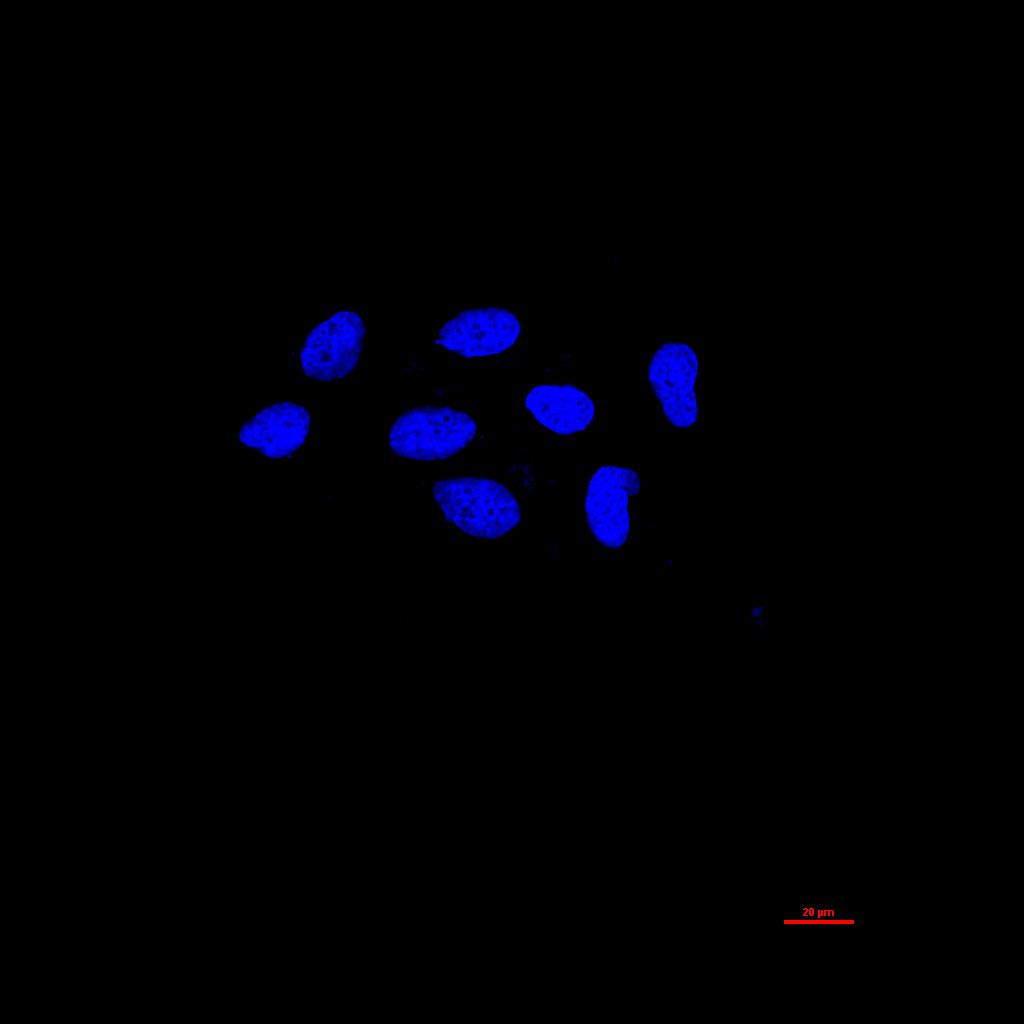

Supplement: Supplementary file 1 [file jox-15-00137-s001.zip › File S1/Figure 6.B) HSF1 siRNA- SiNPs+ DAPI.tif]

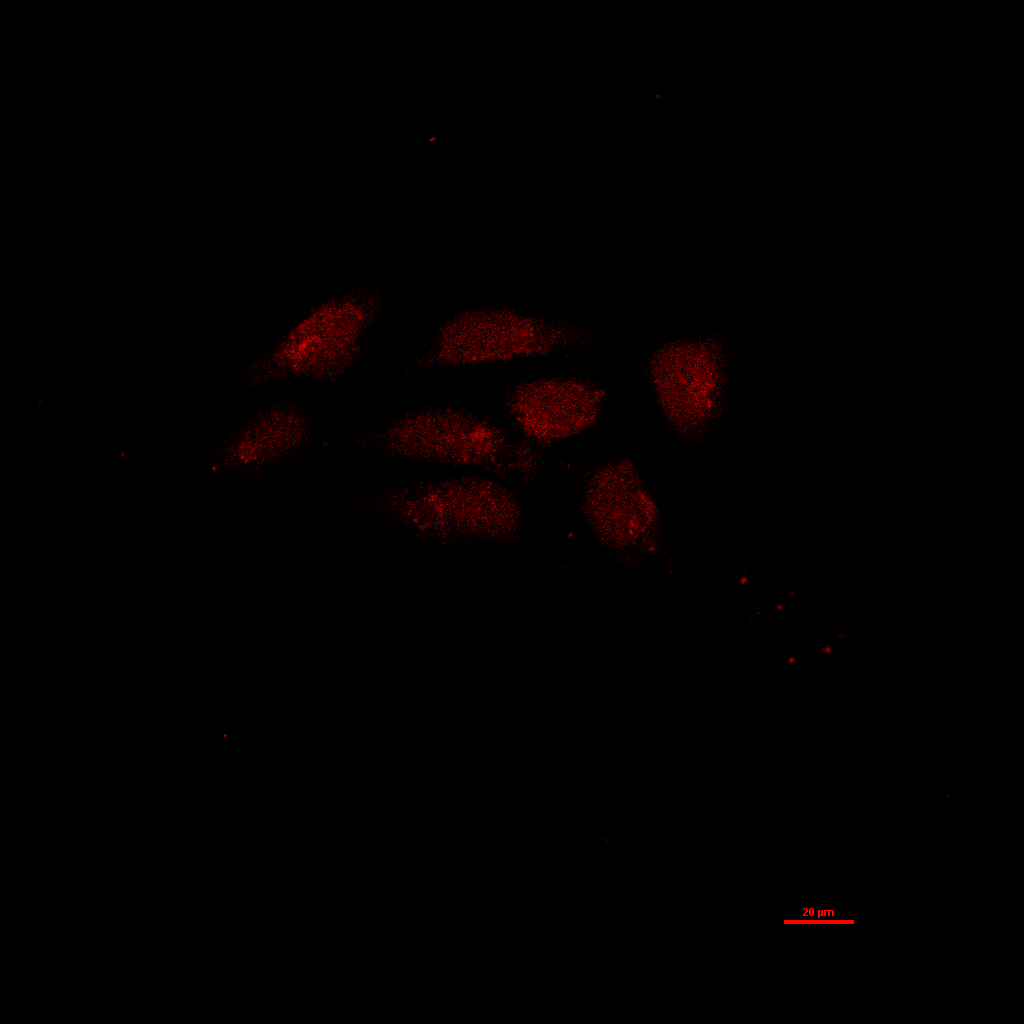

Supplement: Supplementary file 1 [file jox-15-00137-s001.zip › File S1/Figure 6.B) HSF1 siRNA- SiNPs+ HSF1.tif]

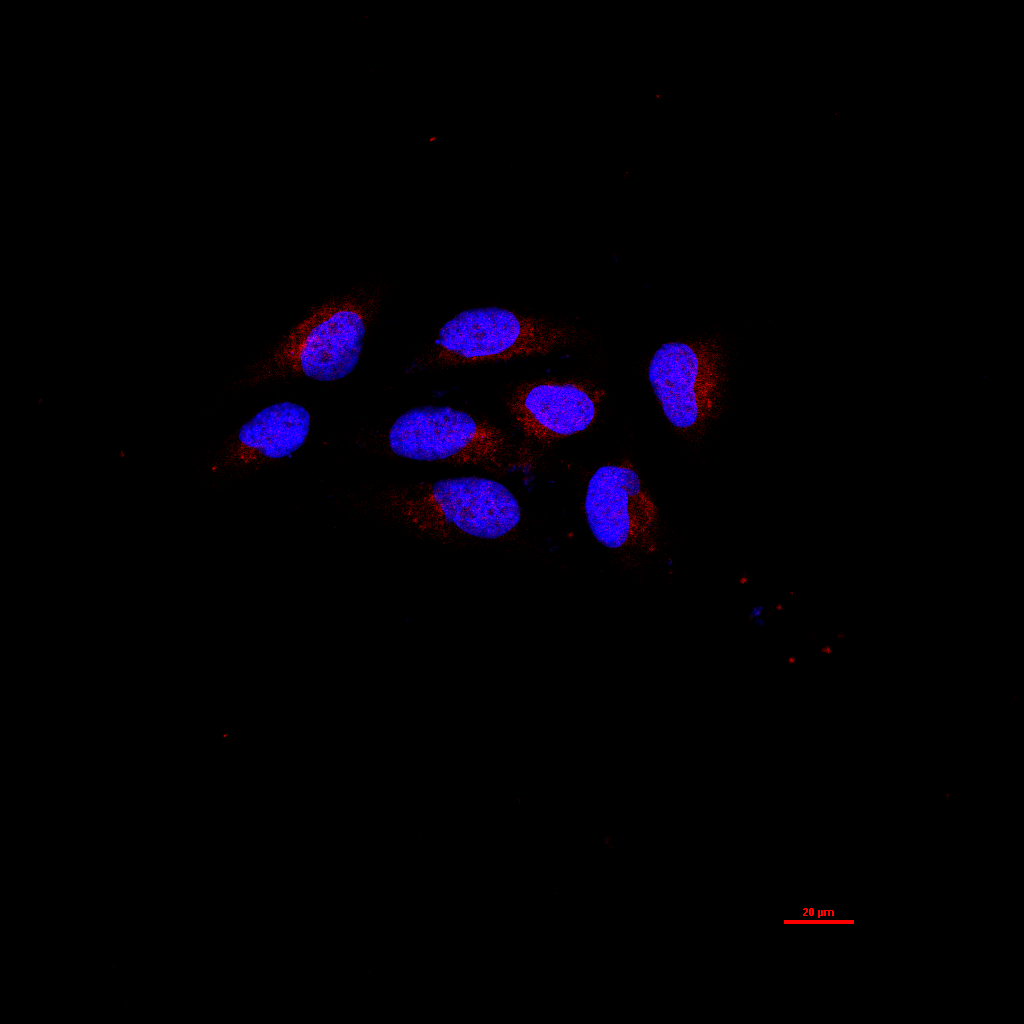

Supplement: Supplementary file 1 [file jox-15-00137-s001.zip › File S1/Figure 6.B) HSF1 siRNA- SiNPs+ Merge.tif]
